# From Bis(borylene)-Substituted Xanthenes as Reactive Intermediates to Diboraoxirane Complexes

**DOI:** 10.1021/jacs.4c17463

**Published:** 2025-02-13

**Authors:** Jun Fan, Sudip Pan, Shenglai Yao, Chengxiang Ding, Gernot Frenking, Matthias Driess

**Affiliations:** † Department of Chemistry: Metalorganics and Inorganic Materials, Technische Universität Berlin, Strasse des 17. Juni 115, Sekr. C2, Berlin 10623, Germany; ‡ Institute of Atomic and Molecular Physics, Jilin University, Changchun 130023, China; § State Key Laboratory of Materials-Oriented Chemical Engineering, School of Chemistry and Molecular Engineering, Nanjing Tech University, Nanjing 211816, China; ∥ Fachbereich Chemie, Philipps-Universität Marburg, Marburg 35032, Germany

## Abstract

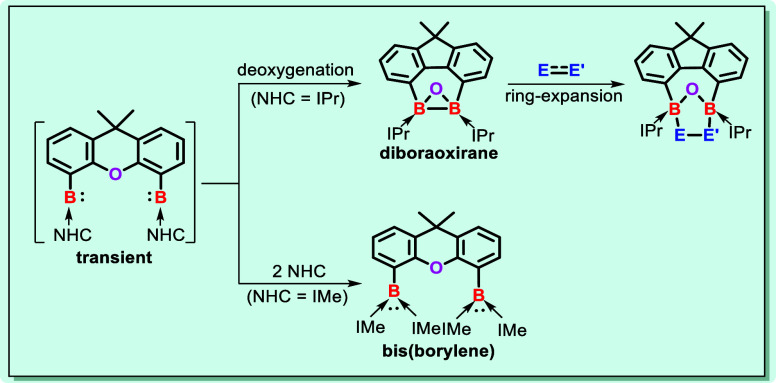

The first *N*-heterocyclic carbene (NHC)-stabilized diboraoxirane complex **4** [NHC = IPr = C{N(iPr)CMe}_2_] was synthesized through the reduction of the corresponding bis(dichloroboryl-IPr)xanthene **3** with potassium graphite. Intriguingly, its formation stems from a diboron(I)-mediated C–O–C deoxygenation of the xanthene spacer via a bis(borylene)xanthene as a reactive intermediate. Consistent with the proposed pathway, bis(borylene)xanthene **6** with three-coordinate B(I) atoms could be isolated when the sterically less demanding NHC ligand IMe [IMe = C{N(Me)CMe}_2_] was employed. Due to its ring strain, the B–B bond of the B_2_O ring in **4** undergoes versatile ring-expansion reactions with small molecules to engender new boron-containing heterocycles. In fact, oxidation of **4** with trimethylamine *N*-oxide, O_2_, and elemental sulfur afforded the unprecedented 1,3-dioxa-2,4-diboretane **7**, 1,3,4-trioxa-2,5-diborolane **8**, and 1-oxa-3,4-dithio-2,5-diborolane **9**, respectively. Moreover, **4** activates isocyanide to produce 1-oxa-2,4-diborete **10** and readily reacts with the C=O groups of benzophenone and CO_2_ to generate the ring-expansion products **11** and **12**, respectively.

## Introduction

**Figure 1:**
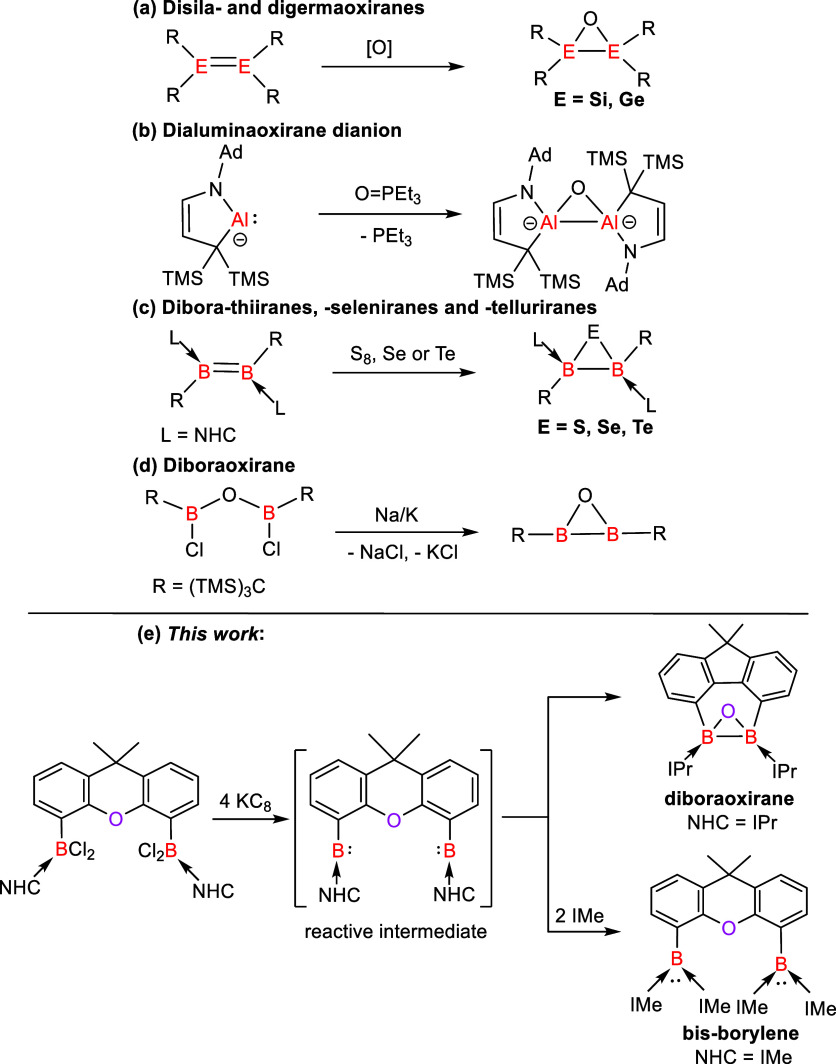
Groups 14 and 13 element hetero-oxiranes. Ad = adamantly; TMS = trimethylsilyl; R = bulky organic groups; NHC = N-heterocyclic carbenes such as IPr = C{N(^i^Pr)CMe}_2_ and IMe = C{N(Me)CMe}_2_.

Oxiranes are an important class of three-membered cyclic ethers that have diverse applications as essential raw materials to produce commercial organic products. Additionally, they also represent important intermediates in organic synthesis.^1−4^ The development of oxirane congeners with a E_2_O framework (E = heavy main-group elements) has attracted considerable attention due to their distinct electronic properties and reactivity.^5,6^ For instance, several examples of heterooxiranes with Group 14 elements (Si and Ge) have been synthesized through monooxygenation of respective dimetallylenes having E=E bonds (Figure 1a).^7−11^ With respect to heavy Group 13 elements, Inoue and co-workers reported the synthesis of a neutral dialuminaoxirane compound through the reaction of an *N*-heterocyclic carbene (NHC)-stabilized dialumene with a stoichiometric amount of *N*-methylmorpholine *N*-oxide. However, this compound could not be structurally characterized due to its fleeting character.^12^ Recently, Kinjo and co-workers isolated a dianionic dialuminaoxirane via oxidative coupling of an alumanyl anion (Figure 1b), which possesses a bent Al–Al bond that undergoes ring-expansion reactions with various unsaturated organic substrates.^13^ To our surprise, diboraoxirane congeners with a B_2_O core remain unknown, and their reactivity is still underexplored, not least because access is a challenge. Inspired by the facile synthesis of isolable E_2_O systems (E = Si, Ge) from monooxygenation of E=E bonds, we envisioned that diboraoxiranes could be produced readily through monooxygenation of the B=B bond of diborenes.^14−16^ In fact, this synthetic method is suitable for the preparation of heavy chalcogen analogues, that is, dibora-thiiranes,^17−19^ -seleniranes,^20,21^ and -telluriranes,^22^ respectively (Figure 1c). However, attempts to realize diboraoxiranes through oxygenation of the B=B bond of a cyclic diborene with O_2_ resulted in the isolation of a 1,3-dioxa-2,4-diboretane featuring a four-membered B_2_O_2_ ring.^21^ This is probably due to the strong oxidizing ability of common oxygen donors, which makes it difficult to control these reactions and selectively form diboraoxiranes. To our knowledge, only one compound with the three-membered B_2_O ring, namely, bis(trisyl)oxadiborirane has been synthesized and structurally characterized to date, in which both boron centers are three-coordinate and adopt a trigonal planar geometry (Figure 1d). Owing to the presence of two bulky tris(trimethylsilyl)methyl (trisyl) groups, this species is very stable and inert even toward water and O_2_.^23,24^

Monocoordinate borylenes of the type R–B (R = organic group) are fleeting species due to the presence of one lone pair of electrons and two vacant 2p orbitals at the boron centers. Addition of a Lewis base to the Lewis-acidic boron(I) center can enhance the stability of borylenes, resulting in isolable donor–acceptor complexes.^25,26^ In 2011, the groundbreaking work of Bertrand and co-workers demonstrated that the parent borylene H–B: can be stabilized by two cyclic (alkyl)(amino)carbenes (cAACs), affording a borylene complex with an amine-like nucleophilic B(I) site.^27^ Since then, a variety of three-coordinate borylenes supported by different Lewis bases^28−33^ could be realized that exhibit a remarkable reactivity, acting as nucleophiles and reducing agents.^34−37^ Additionally, Bertrand, Stephan, and co-workers reported the first two-coordinate aminoborylene stabilized by a single cAAC, which shows a transition-metal-like reactivity toward dihydrogen and carbon monoxide.^38^ Recently, the Braunschweig group discovered that two-coordinate cAAC-supported aryl-substituted borylenes are capable of activating and functionalizing dinitrogen.^39−42^ Despite these seminal advances, bidentate NHC-stabilized bis(borylenes) remain scarce. The latter feature two boron(I) centers that are expected to exhibit cooperative reactivity in small-molecule activation; they may serve as powerful ligands toward low-valent main-group and transition-metal centers akin to isolable bis(tetrylene) ligands.^43−46^

Herein, we report the synthesis of the peerless NHC-stabilized diboraoxirane **4** [NHC = IPr = C{N(iPr)CMe}_2_] through the reduction of the bis(dichloroboryl-IPr)xanthene adduct **3** with potassium graphite (KC_8_). Remarkably, the dechlorination of **3** caused a B–B bond formation along with the C–O–C deoxygenation of the xanthene moiety to afford **4** most likely via the corresponding bis(borylene-IPr)xanthene complex as a reactive intermediate, which is reminiscent of the formation of dianionic dialuminaoxirane through the deoxygenation of O = PEt_3_ by two aluminum(I) centers (Figure 1b). In fact, the related bis(borylene) adduct **6** could be trapped when the sterically less demanding *N*-heterocyclic carbene IMe [IMe =:C{N(Me)CMe}_2_] was employed (Figure 1e). Due to the high ring strain of **4**, the B–B bond is capable of activating small molecules, affording novel types of ring-expansion products.

## Results and Discussion

### Synthesis of Diboraoxirane Complex **4** through Diboron(I)-Mediated Deoxygenation of the Xanthene Moiety

At first, the diborylation of xanthene to form BCl_2_(Xant)BCl_2_ (Xant = 9,9-dimethyl-xanthene-4,5-diyl) was achieved by reaction of 4,5-bis(trimethylsilyl)Xant with excess BCl_3._^47^ BCl_2_(Xant)BCl_2_ was allowed to react with 2 M equiv of IDip (IDip = C{N(Ar)CH}_2_, Ar = 2,6-*i*Pr_2_C_6_H_3_) to furnish the bis(dichloroboryl-IDip)xanthene complex **1** in a 94% yield (see the Supporting Information). The high-field resonance signal (δ 1.3 ppm) in the ^11^B{^1^H} NMR spectrum of **1** indicates the presence of two four-coordinate boron atoms; this was confirmed by single-crystal X-ray diffraction (XRD) analysis (Figure S44).

**Scheme 1:**
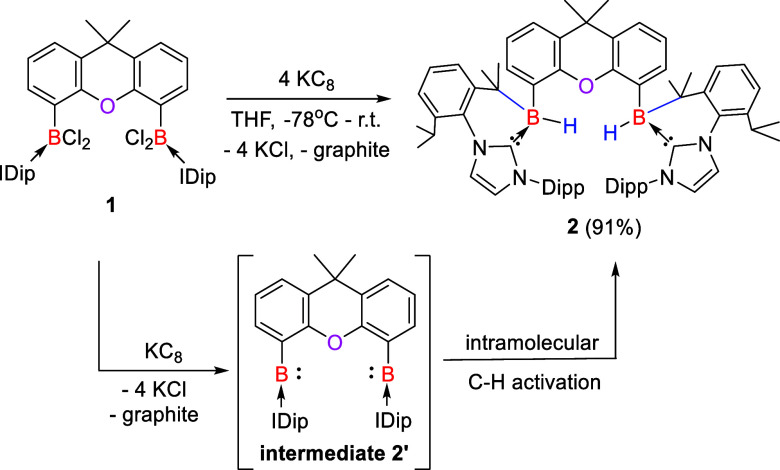
Formation of **2** from 1 via the Proposed Bis(borylene) Intermediate **2**′ [IDip = C{N(Ar)CH}_2_, Ar = 2,6-*i*Pr_2_C_6_H_3_]

**Figure 2:**
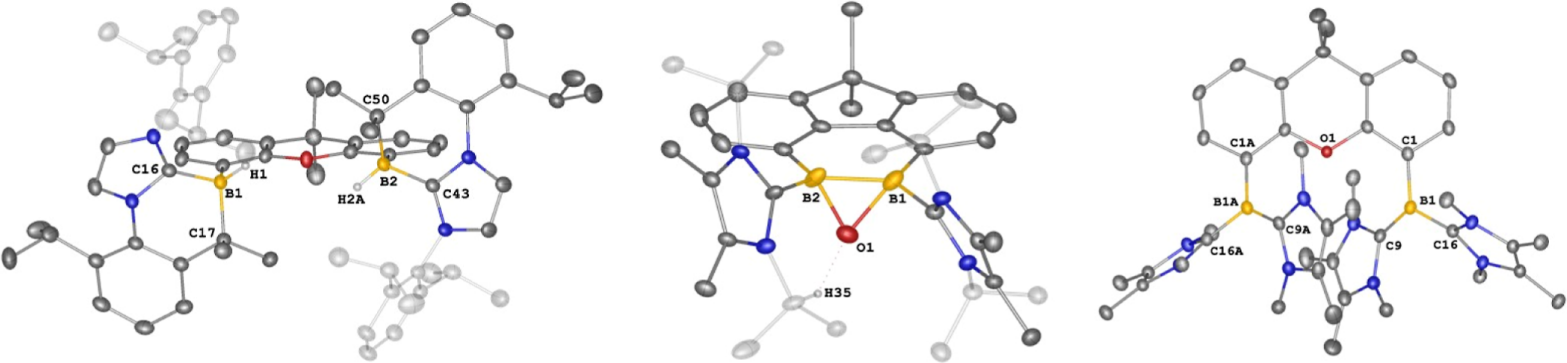
Molecular structures of **2** (left), **4** (middle, the entire molecule is disordered over two orientations with a ratio of 32:68%), and **6** (right). Thermal ellipsoids are drawn at the 50% probability level. H atoms and solvent molecules are omitted for clarity. Selected bond lengths (Å) and angles (^o^): **2**: B1–H1 1.0000, B1–C16 1.605(3), B1–C17 1.665(4), C16–B1–C17 114.89(19). **4**: B1–B2 1.725(10), B1–O1 1.662(10), B2–O1 1.566(10), B2–O1–B1 64.5(4), O1–B2–B1 60.5(4), O1–B1–B2 55.0(4). **6**: C1–B1 1.5773(19), C9–B1 1.5114(19), C16–B1 1.5327(19), C9–B1–C16 117.38(11), C1–B1–C16 116.02(11), and C1–B1–C9 125.78(11).

The reduction of **1** with 4 M equiv of KC_8_ in THF at −78 °C afforded a reddish-purple suspension. The mixture turned yellow when it was allowed to warm to ambient temperature. After a workup, compound **2** was isolated as a colorless solid in a 91% yield (Scheme 1). The ^11^B{^1^H} NMR spectrum of **2** shows a signal at δ −18.6 ppm. Its molecular structure obtained by XRD analysis shows that each boron atom inserted into a Me_2_C–H bond of the isopropyl groups of IDip, affording two six-membered C_4_NB rings (Figure 2, left). We proposed that the reductive dechlorination of **1** with KC_8_ generated the target IDip-stabilized two-coordinate bis(borylene) **2′** as a reactive intermediate that underwent intramolecular C–H bond activation to form **2**. Similar C–H bond activation mediated by transient two-coordinate borylenes were reported previously.^25,39,47,48^

**Scheme 2:**
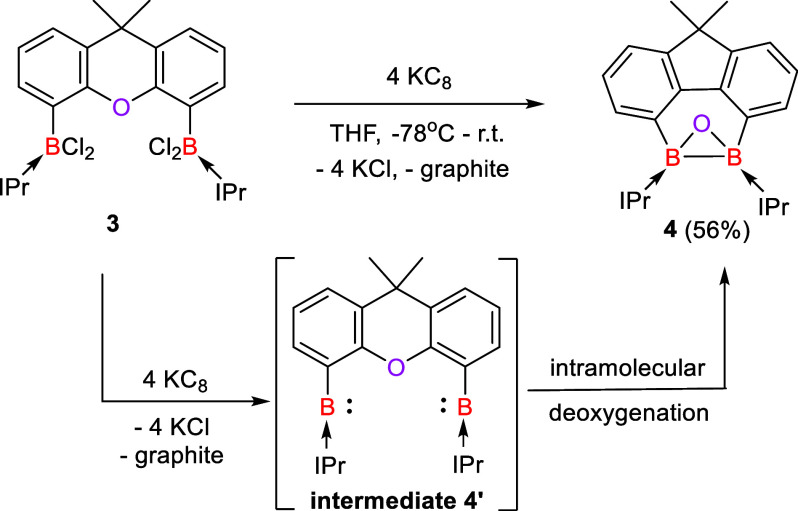
Formation of **4** from **3** via the Proposed Bis(borylene) Intermediate **4**′ (IPr = C{N(iPr)CMe}_2_)

Because the proposed bis(borylene) intermediate **2′** is sterically congested which facilitates the Me_2_C–H activation, we envisaged that using the sterically less demanding IPr in the bis(boryl-IPr) complex **3** may lead to the observable two-coordinate bis(borylene) **4’**. The starting material **3** was synthesized in a manner similar to that of **1** and characterized by NMR spectroscopy and XRD analysis (Figure S46). The dechlorination of **3** with 4 M equivs of KC_8_ in THF at −78 °C afforded also a reddish-purple suspension. The latter was allowed to warm up to room temperature and stirred for 12 h, resulting in an orange-red mixture. Remarkably, the reaction produced the IPr-stabilized diboraoxirane **4** through diboron(I)-mediated C–O–C deoxygenation of the xanthene moiety. The product was isolated as a red solid in a 56% yield (Scheme 2). Its ^11^B{^1^H} NMR spectrum exhibits a signal at δ −9.8 ppm, which is significantly shifted to a higher field compared to that of bis(trisyl)oxadiborirane (δ 65.7 ppm).^23^ To explore the reaction mechanism, we employed also two equiv of KC_8_ under the same conditions. The observed color change during the first 30 min was identical to that seen when four equiv of KC_8_ were used. The in situ ^11^B{^1^H} NMR spectrum revealed that the major products remained compound **4**, along with the unreacted compound **3**. However, after an additional 2 h, the reaction mixture became complex, suggesting that **4** is unstable in the presence of **3**, which contains B–Cl bonds. **4** was found to be decomposed in DCM-*d*_2_ during NMR measurements.

The molecular structure of **4** revealed that the two boron centers are bonded to a planar fluorene scaffold and constitute a three-membered B_2_O ring (Figure 2, middle). These two ring planes are almost perpendicular with a dihedral angle of 91.9°. The entire molecule is disordered over two orientations with a ratio of 32:68%, reminiscent of the structural features observed for bis(trisyl)oxadiborirane.^23^ Two C–H bonds in the isopropyl groups of IPr are oriented toward the O atom on both sides and indicate a weak hydrogen bond interaction. Because of the disorder, a discussion of bond lengths and angles is meaningless.

Recently, our group demonstrated that the reduction of a bis(silylene)-stabilized bis(dibromoboryl)xanthene with KC_8_ afforded a silylene-stabilized diborene species (Figure 5, right), which can be viewed as the dimerization product of the corresponding two-coordinate bis(borylene). Furthermore, this compound can undergo reductive deoxygenation of the xanthene backbone, facilitated by the silylene moiety, resulting in the formation of diboraphenanthrene derivatives.^49^ In contrast, replacing the silylene with a less sterically demanding and weaker σ-donating IPr ligand results in the formation of diboraoxirane 4 instead of a diborene. The formation of 4 involves a similar deoxygenation process, which is mediated by two boron(I) centers. In other words, akin to the formation of **2**, we propose that the formation of **4** proceeds via the bis(borylene) **4′** as a reactive intermediate that is capable of deoxygenating the C–O–C moiety in the xanthene backbone.

### Trapping a Bis(borylene-NHC) Intermediate

**Scheme 3:**
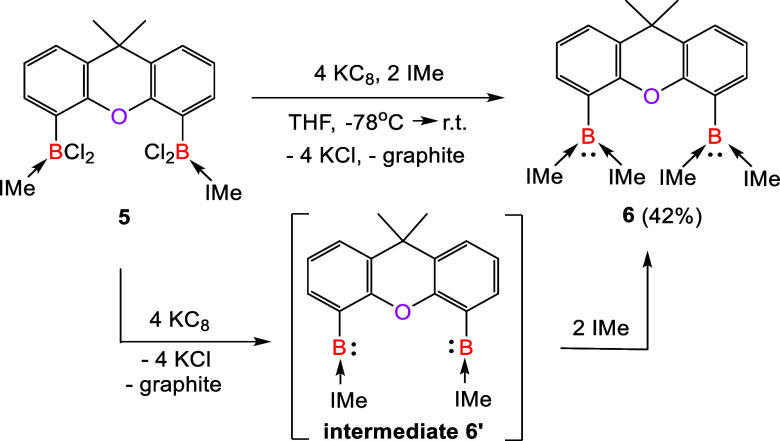
Formation of **6** from **5** via the Proposed Bis(borylene) Intermediate **6**′ [IMe = C{N(Me)CMe}_2_]

We also probed the suitability of the sterically less demanding IMe to trap a two-coordinate bis(borylene-NHC) intermediate. The starting material, the bis[dichloroboryl-IMe]xanthene complex **5**, was also synthesized and characterized by multinuclear NMR spectroscopy and XRD analysis (Figure S48). The reductive dechlorination of **5** with various reducing agents resulted in an unidentified mixture. However, the reduction of **5** with 4 M equivs of KC_8_ in the presence of two additional molar equiv of IMe in THF afforded the tetrakis(IMe)-supported bis(borylene) **6** as a red solid in a 42% yield (Scheme 3). The ^11^B{^1^H} NMR spectrum of **6** shows a singlet at δ −4.1 ppm, falling in the range of values reported for bis(NHC)-stabilized borylenes (δ −6.8 to 1.6 ppm).^34^ An XRD analysis of **6** uncovered that each boron center is coordinated with two IMe ligands and adopts a trigonal planar geometry (Figure 2, right). The C_IMe_–B distances in **6** (1.5114(19) and 1.5327(19) Å) are shorter than the C_xanthene_-B length (1.5773(19) Å), but similar to those observed in bis(IMe)-stabilized borylenes (1.519(3) – 1.520(3) Å).^34^ The isolation of **6** supports that the bis(borylene-IMe) complex **6′** or diborene **6″** (Figure 5, left) could be formed as a reactive intermediate.

Our attempts to synthesize the analogous bis(borylene) complexes through the reduction of **1** and **3** with KC_8_ in the presence of excess IMe failed, most likely due to the larger steric congestion from the IDip and IPr ligands. While the borylene chemistry has been rapidly developed in recent decades, isolable bis(borylene) complexes are still scarce. To our knowledge, only one CO– and cAAC-stabilized bis(borylene)naphthalene species have been reported to date.^50^ Compound **6** is the first bis(borylene) complex stabilized by four weak π-accepting IMe ligands.

### Ring-Expansion Reactions of **4** with Small Molecules Leading to Unique Boron-Containing Heterocycles

**Scheme 4:**
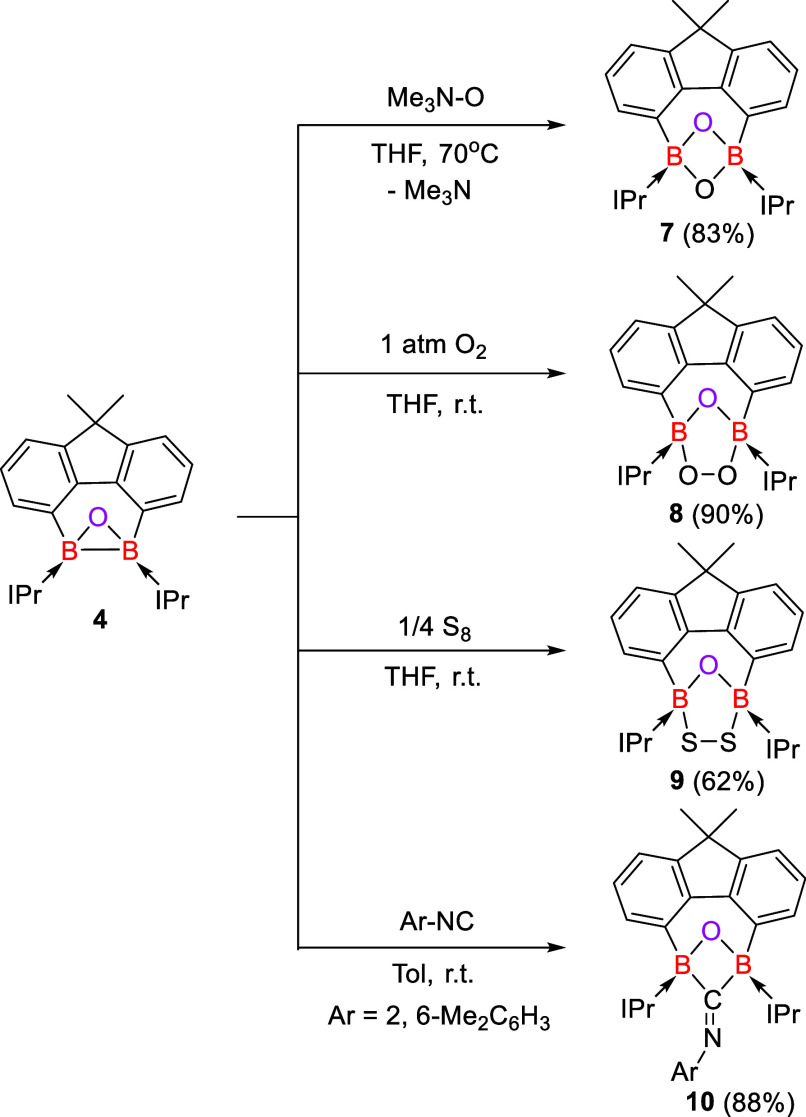
Ring-Expansion Reactions of **4** with Me_3_N–O, O_2_, S_8_ and Isocyanide

**Figure 3:**
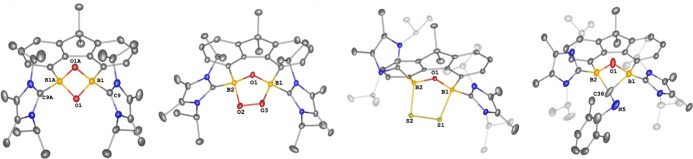
Molecular structures of **7**, **8**, **9**, and **10**. Thermal ellipsoids are drawn at the 50% probability level. H atoms and solvent molecules are omitted for clarity. Selected bond lengths (Å) and angles (^o^): **7**: O1–B1 1.484(3), B1–C9 1.648(5), B1–O1–B1A 83.2(3), O1–B1–O1A 95.3(3). **8**: B2–O1 1.446(4), B2–O2 1.491(4), O2–O3 1.437(3), B1–O1 1.451(4), B1–O3 1.504(4), O1–B1–O3 106.2(2), O1–B2–O2 105.2(2), B2–O1–B1 103.9(2), O3–O2–B2 102.1(2), O2–O3–B1 107.1(2). **9**: S1–S2 2.0688(14), S1–B1 2.049(5), S2–B2 1.990(5), O1–B1 1.437(5), O1–B2 1.428(5), B1–S1–S2 96.17(14), B2–S2–S1 88.92(14), B2–O1–B1 118.2(3). **10**: O1–B1 1.485(7), O1–B2 1.488(8), B2–C38–B1 79.6(5), O1–B1–C38 82.3(4), B1–O1–B2 99.7(4), and O1–B2–C38 92.9(5).

Due to their higher ring strain, boron-containing three-membered ring species are expected to show a higher reactivity toward small molecules than oxiranes.^51−53^ This prompted us to investigate the reactivity of **4** (Scheme 4) toward oxidants. In fact, it undergoes facile reaction with 1 M equiv of trimethylamine *N*-oxide (Me_3_NO) in THF at 70 °C to afford the 1,3-dioxa-2,4-diboretane **7** as a colorless solid in 83% yield. In this reaction, the B–B bond of **4** was monooxygenated, resulting in the B–O–B moiety. The ^11^B{^1^H} NMR spectrum of **7** shows a resonance at δ 8.5 ppm, which is downfield shifted compared to that of **4** (δ −9.8 ppm). The molecular structure of **7** consists of a four-membered B_2_O_2_ core (Figure 3). The related 1,3-dioxa-2,4-diboretanes have been reported previously,^21,54^ but most of them feature three-coordinate boron atoms.

The activation of O_2_ by oxygenase metalloenzymes is an important process in biological chemistry.^55^ Various boron species have been utilized to activate O_2_ for mimicking such an enzymatic processes.^56−58^ However, the activation of O_2_ by a B–B-bonded compound has not been reported as of yet. We learned that the B–B bond in **4** reacts readily with O_2_ (1 atm) in THF at ambient temperature to form the 1,3,4-trioxa-2,5-diborolane **8**, which was isolated as a colorless solid in 90% yield. Its ^11^B{^1^H} NMR signal at δ 5.1 ppm is very close to that of **7** (δ 8.5 ppm). Compound **8** consisted of a five-membered B_2_O_3_ ring with a diboraperoxide B–O–O–B moiety in which both boron atoms adopt a tetrahedral coordination geometry. O_2_ reduction to peroxide compounds mediated by main-group species is a significant chemical transformation. Several examples of diboraperoxide derivatives have been synthesized through the O_2_ activation by spatially separated diboron species.^59−61^ In this work, the B–B bond in **4** engenders direct diboraperoxide formation.

Similarly, treatment of **4** with 1/4 molar equivs of elemental sulfur (S_8_) in THF at room temperature furnished the 1-oxa-3,4-dithio-2,5-diborolane **9** in a 62% yield, where two S atoms were inserted into the B–B bond of **4**. The ^11^B{^1^H} NMR spectrum of **9** shows a signal at δ = 4.9 ppm, which is akin to that of **8** (δ = 5.1 ppm). Its molecular structure shows a similar five-membered ring in which two oxygen atoms in **8** were replaced by two sulfur atoms (Figure 3). While 1,3,4-trithio-2,5-diborolanes and 1,3,4-triseleno-2,5-diborolanes have previously been synthesized through oxidation of the B=B bond of diborenes with elemental chalcogens (S and Se),^17^**8** and **9** represent the first examples of 1,3,4-trioxa-2,5-diborolane and 1-oxa-3,4-dithio-2,5-diborolane derivatives, respectively.

The B–B bond of **4** shows also a high reactivity toward isocyanides. The reaction of **4** with 1 M equiv of 2,6-dimethylphenyl isocyanide in toluene at room temperature afforded a yellow solution, from which **10** was isolated as a yellow solid in a 88% yield. Compound **10** is a 1-oxa-2,4-diborete derivative with a four-membered CB_2_O ring (see its molecular XRD structure in Figure 3) that resulted from the insertion of the C atom of the isocyanide group into the B–B bond of **4**. Although the insertion of isocyanides into B–B single or multiple bonds are well documented,^62−64^**10** represents a rare example of its type.

**Scheme 5:**
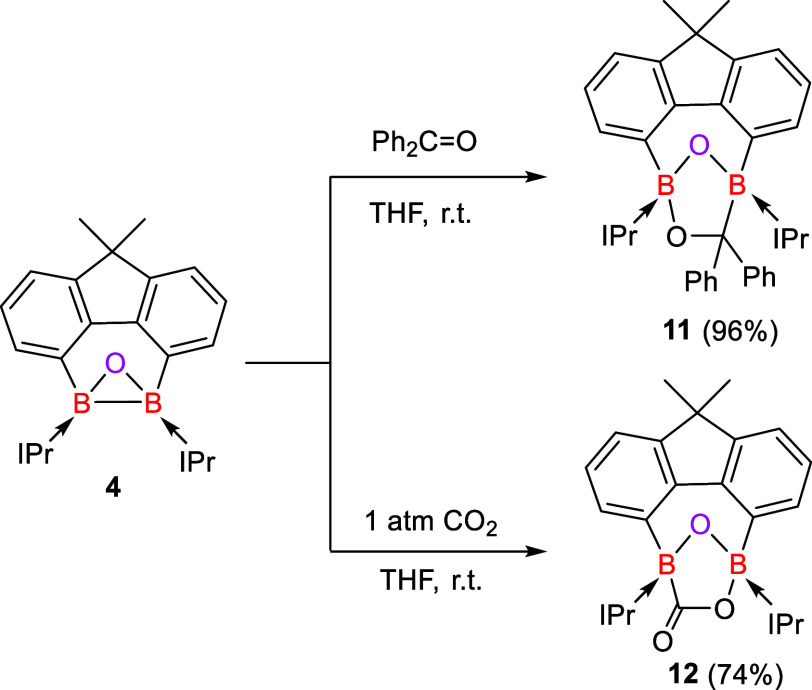
Diborylation Reactions of **4** with Benzophenone and CO_2_

**Figure 4:**
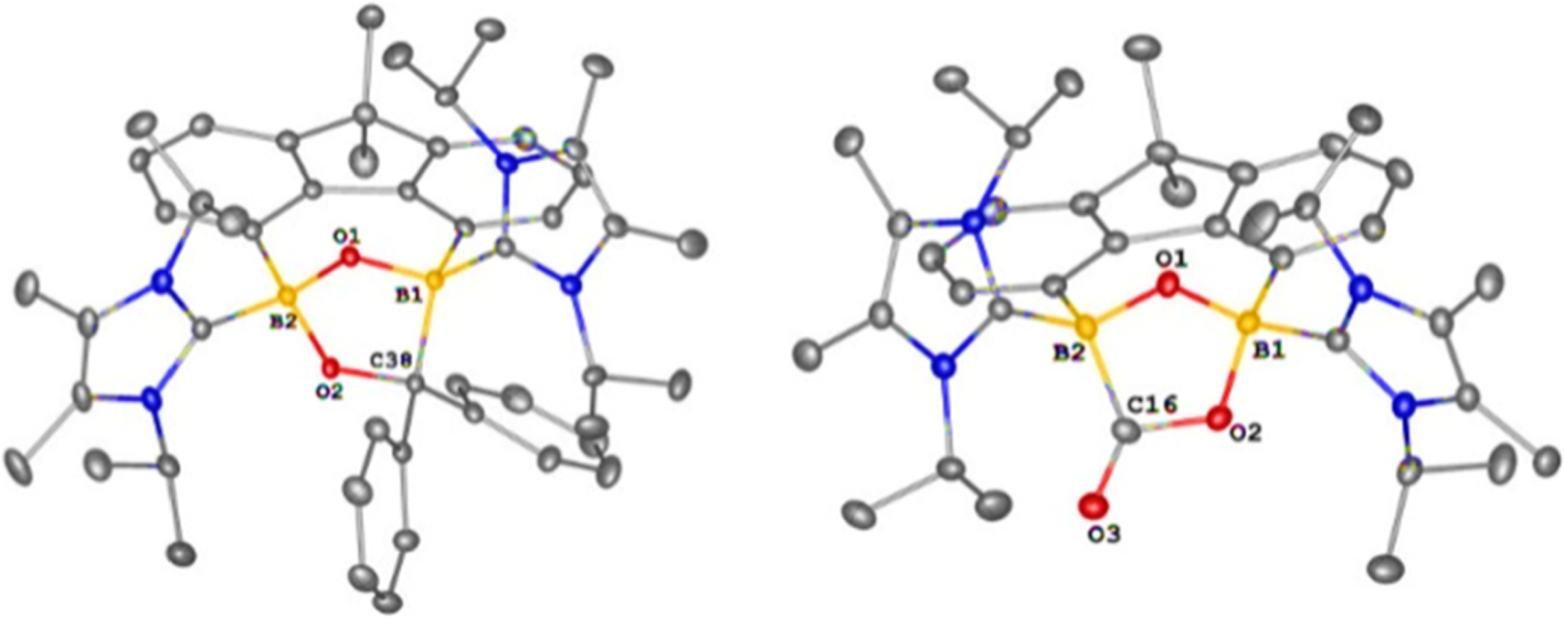
Molecular structures of **11** (left) and **12** (right). Thermal ellipsoids are drawn at the 50% probability level. H atoms and solvent molecules are omitted for clarity. Selected bond lengths (Å) and angles (^o^): **11**: O1–B1 1.4607(18), O1–B2 1.4496(18), O2–C38 1.4566(16), O2–B2 1.4869(18), C38–B1 1.715(2), B2–O1–B1 107.38(11), C38–O2–B2 111.21(10), O2–C38–B1 96.67(10), O1–B1–C38 100.39(11), O1–B2–O2 106.93(11). **12**: O1–B1 1.444(2), O1–B2 1.461(3), O2–C16 1.364(3), O2–B1 1.521(3), O3–C16 1.216(3), C16–B2 1.650(3), B1–O1–B2 107.90(15), and C16–O2–B1 110.62(15).

Remarkably, the B–B bond of **4** can also undergo spontaneous diborylation reactions with the C=O bonds of ketones and CO_2_, affording ring-expansion products (Scheme 5). Treatment of **4** with 1 M equiv of benzophenone in THF at room temperature produced the diborylation product **11**, which was isolated as a colorless solid in 96% yield. The ^11^B{^1^H} NMR spectrum of **11** shows two resonances at δ 6.8 and −4.0 ppm. Its molecular structure of **11** features a five-membered B_2_O_2_C ring (Figure 4, left).

The reaction of **4** with CO_2_ (1 atm) in THF at ambient temperature resulted in a color change of the orange-red solution to yellow. After workup, the diborylated product **12** was isolated as a colorless solid in 74% yield. Its ^11^B{^1^H} NMR spectrum shows two signals at δ 5.9 and 3.0 ppm, which are comparable to those of **11** (δ 6.8 and −4.0 ppm). The molecular structure of **12** features a five-membered B_2_O_2_C ring akin to that of **11** and an exocyclic C=O (1.216(3) Å) bond (Figure 4, right). The results are reminiscent of the CO_2_ activation mode by the 1,2-disila-3-oxirane.^11^

### Density Functional Theory Calculations

We carried out density functional theory (DFT) calculations at the BP86-D3(BJ) level using various basis sets in order to shed light on reactions **3** → **4** and **5** → **6** as well as the electronic structures of **4** and **6** (see the Supporting Information). The calculated bond lengths and angles of **3** and **6** are in good agreement with the experimental values (Figure S56). The theoretical values for **4** significantly deviate from the metric values obtained by X-ray structure analysis due to disordering. The identical B1–O1 and B2–O1 distances of 1.466 Å are much shorter than the experimentally derived unequal bond lengths (1.566 and 1.662 Å). The calculated bond lengths and angles of **4** may, therefore, be used as reliable values for the geometry of the compound. We do not discuss the results of the bonding situation in the molecules in this work because we mainly focus on the reaction mechanisms. A detailed analysis of the electronic structure will be presented in a forthcoming theoretical study.

**Figure 5:**
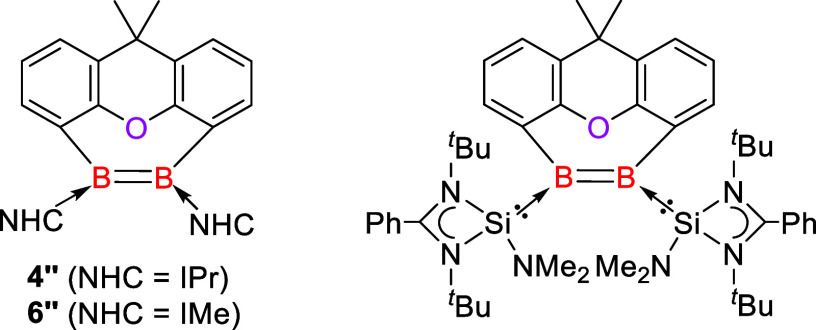
Structures of **4″**, **6″** and related isolable silylene-diborene.

The mechanism of the dichlorination of **3** and **5** with KC_8_ is still unknown. We propose that the reactions proceed via the bis(borylene) species **4′** and **6′** as first reactive intermediates, which have rather short B–C distances in their optimized geometries. Notably, the bis(borylene) species **4′** and **6′** have only shallow minima on the potential energy surface, which easily built the diborene species **4″** and **6″** possessing short B–B distances of 1.660 and 1.664 Å, respectively (Figure 5, left). The latter diborenes **4″** and **6″** are 69.1 and 68.1 kcal mol^–1^ lower in energy than the bis(borylenes) **4′** and **6’** (Figure S61). Accordingly, we envisaged that the reduction of **5** affords bis(borylene) **6′** and/or diborene **6″** as intermediates. Remarkably, the reaction of diborene **6″** with two IMe ligands to afford bis(borylene) **6** as the final product is exergonic by Δ*G* = −73.0 kcal mol^–1^.

The related reaction of the IPr-ligated complex **3** gives compound **4** as the product, which involves not only dechlorination but also oxygen 3,3'-migration. It is peculiar that **3** and **5**, which differ only in the slightly less bulky ligand, exhibit vastly different reactivities. We calculated the analogous reaction of **3** giving the complex **4** (4-IPr) (Figure S56) with two IPr ligands at each boron atoms via the putative diborene intermediate **4″**, which is experimentally not observed. This reaction is exergonic at Δ*G* = −57.0 kcal mol^–1^.

We then calculated the experimentally observed reaction of **3** giving complex **4** via putative intermediate **4″** and the related but not observed reaction of **5** via putative intermediate **6″**, which leads to **6** (B–O–B) (Figure S56), where the oxygen atom is bonded to boron. These two reactions are exergonic by Δ*G* values of −87.7 and −86.8 kcal mol^–1^, respectively.

Similar reaction energies of these reactions suggest that the different reaction processes of **3** and **5** do not have a thermodynamic origin. We previously isolated a xanthene-based silylene-diborene species (Figure 5, right) through the reduction of a similar bis(silylene)-stabilized bis(dibromoboryl)xanthene with KC_8_, which could undergo various types of deoxygenations.^49^ The related diborene species **4″**, featuring a less sterically demanding and weaker σ-donating IPr ligand, is expected to be more reactive than the silylene-diborene derivative.

**Figure 6:**
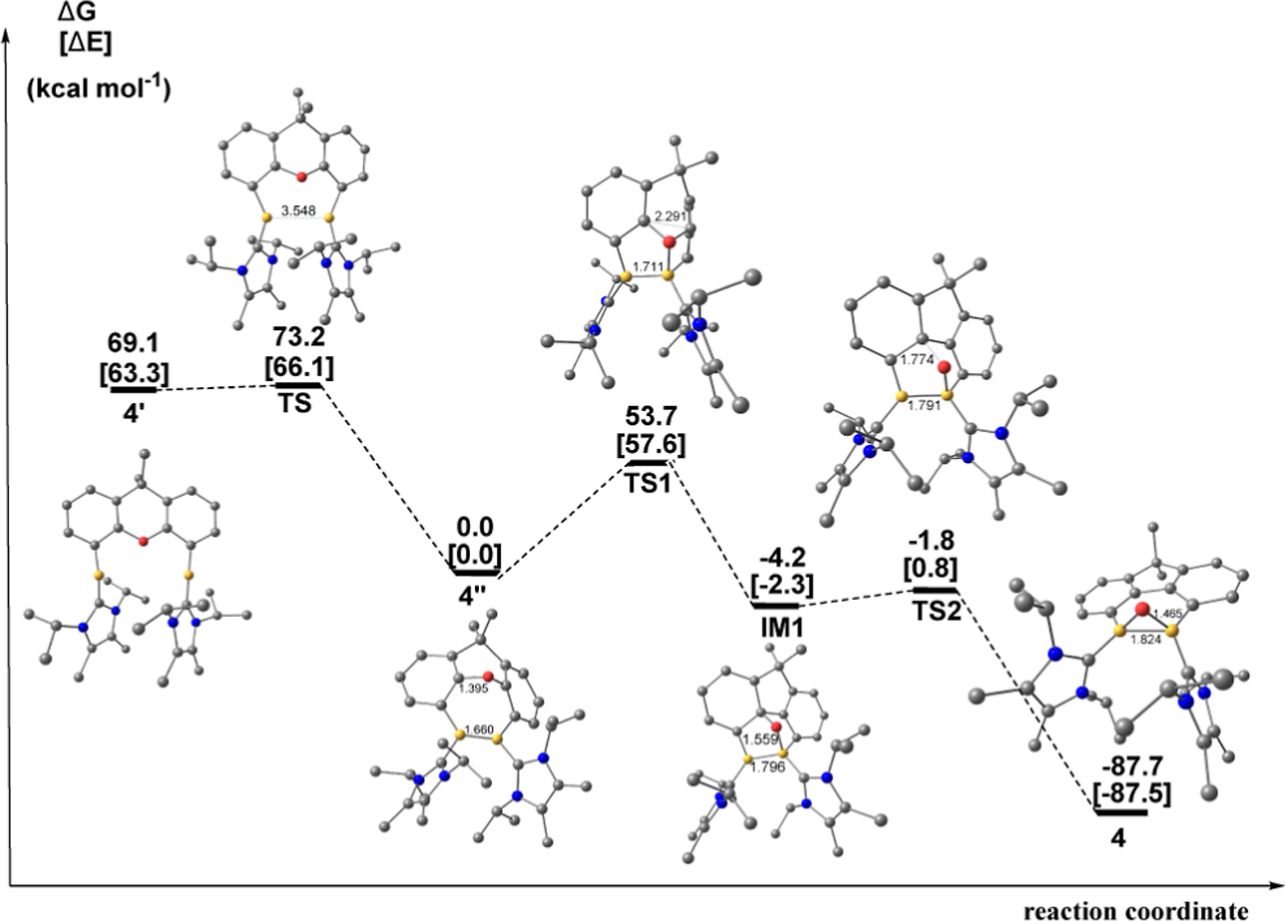
Calculated free energy profile for the formation of complex **4** at the BP86-D3(BJ)/def2-SVP level which gives likely and unlikely intermediates and transition states. Key bond distances are given in Å. Hydrogen atoms have been omitted for clarity.

We still do not know the mechanism for the reaction **4″** → **4** but performed calculations on a possible reaction pathway **4″** → **4**, which gives likely and unlikely intermediates and transition states (Figure 6). The strongly exergonic reaction consists of a 3,3' migration, where an oxygen atom migrates from a C–O–C position to a B–O–B placement. The calculations indicate that the reaction proceeds with two 1,3-migrations via **IM1**. The second step has a very low activation barrier, but the first step **4″** → **IM1** has a too high barrier of Δ*G*^≠^ = 53.7 kcal mol^–1^ (**TS1**), which does not agree with the experimental observations. We did not find a transition state for a direct reaction **4″** → **4** nor for other opening steps with a lower barrier than **TS1**. It is possible that KC_8_ may be involved in an unspecified way in the facile oxygen migration reaction, which takes place in the reaction **3** → **4**.

## Conclusions

In conclusion, the reduction of the bis(IDip)- and bis(IPr)-BCl_2_(Xant)BCl_2_ complexes with KC_8_ in THF yielded the C–H bond activation product **2** and diboraoxirane **4**, respectively. We proposed that the formation of **2** and **4** proceeds via two-coordinate bulky NHC-supported bis(borylene) complexes as reactive intermediates. Remarkably, employing the sterically less demanding N-heterocyclic carbene ligand IMe allowed the synthesis and isolation of the first three-coordinate bis(borylene) complex **6**. Due to its high ring strain, the B(sp^3^)-B(sp^3^) bond in **4** shows a remarkable reactivity toward small molecules such as Me_3_NO, O_2_, elemental sulfur, isocyanide, benzophenone, and CO_2_, to afford a series of novel diboron-containing heterocycles.

## Supporting Information Available

Experimental details, materials and methods, spectroscopic data, and DFT calculations (PDF)

## References

[ref1] Lewars E. G. Oxirenes. Chem. Rev. 1983, 83, 519–534. 10.1021/cr00057a002.

[ref2] Capriati V.; Florio S.; Luisi R. α-Substituted α-Lithiated Oxiranes: Useful Reactive Intermediates. Chem. Rev. 2008, 108, 1918–1942. 10.1021/cr0683921. 18543876

[ref3] Gorzynski Smith J. Synthetically Useful Reactions of Epoxides. Synthesis 1984, 1984, 629–656. 10.1055/s-1984-30921.

[ref4] Hanif M.; Zahoor A. F.; Saif M. J.; Nazeer U.; Ali K. G.; Parveen B.; Mansha A.; Chaudhry A. R.; Irfan A. Exploring the synthetic potential of epoxide ring opening reactions toward the synthesis of alkaloids and terpenoids: a review. RSC Adv. 2024, 14, 13100–13128. 10.1039/D4RA01834F. 38655462 PMC11036177

[ref5] He G.; Shynkaruk O.; Lui M. W.; Rivard E. Small Inorganic Rings in the 21st Century: From Fleeting Intermediates to Novel Isolable Entities. Chem. Rev. 2014, 114, 7815–7880. 10.1021/cr400547x. 24605824

[ref6] Yan C.; Kinjo R. Three-Membered Aluminacycles. J. Am. Chem. Soc. 2023, 145, 12967–12986. 10.1021/jacs.3c03152. 37289812

[ref7] Yokelson H. B.; Millevolte A. J.; Gillette G. R.; West R. Disilaoxiranes: synthesis and crystal structure. J. Am. Chem. Soc. 1987, 109, 6865–6866. 10.1021/ja00256a058.

[ref8] McKillop K. L.; Gillette G. R.; Powell D. R.; West R. 1,2-Disiladioxetanes: structure, rearrangement and reactivity. J. Am. Chem. Soc. 1992, 114, 5203–5208. 10.1021/ja00039a035.

[ref9] Ando W.; Kako M.; Akasaka T.; Nagase S. Reaction of singlet oxygen with disiliranes: dioxygen insertion into silicon-silicon. sigma. bonds. Organometallics 1993, 12, 1514–1522. 10.1021/om00029a009.

[ref10] Masamune S.; Batcheller S. A.; Park J.; Davis W. M.; Yamashita O.; Ohta Y.; Kabe Y. Oxygenation of digermene derivatives. J. Am. Chem. Soc. 1989, 111, 1888–1889. 10.1021/ja00187a058.

[ref11] Wendel D.; Szilvási T.; Henschel D.; Altmann P. J.; Jandl C.; Inoue S.; Rieger B. Precise Activation of Ammonia and Carbon Dioxide by an Iminodisilene. Angew. Chem., Int. Ed. 2018, 57, 14575–14579. 10.1002/anie.201804472. 29920891

[ref12] Weetman C.; Porzelt A.; Bag P.; Hanusch F.; Inoue S. Dialumenes - aryl vs. silyl stabilisation for small molecule activation and catalysis. Chem. Sci. 2020, 11, 4817–4827. 10.1039/D0SC01561J. 34122939 PMC8159210

[ref13] Koshino K.; Kinjo R. A Highly Strained Al–Al σ-Bond in Dianionic Aluminum Analog of Oxirane for Molecule Activation. J. Am. Chem. Soc. 2021, 143, 18172–18180. 10.1021/jacs.1c07389. 34697939

[ref14] Braunschweig H.; Dewhurst R. D. Boron–Boron Multiple Bonding: From Charged to Neutral and Back Again. Organometallics 2014, 33, 6271–6277. 10.1021/om500875g.

[ref15] Braunschweig H.; Dewhurst R. D. Single, Double, Triple Bonds and Chains: The Formation of Electron-Precise B-B Bonds. Angew. Chem., Int. Ed. 2013, 52, 3574–3583. 10.1002/anie.201208189. 23362015

[ref16] Arrowsmith M.; Braunschweig H.; Stennett T. E. Formation and Reactivity of Electron-Precise B-B Single and Multiple Bonds. Angew. Chem., Int. Ed. 2017, 56, 96–115. 10.1002/anie.201610072. 27860056

[ref17] Auerhammer D.; Arrowsmith M.; Dewhurst R. D.; Kupfer T.; Bohnke J.; Braunschweig H. Closely related yet different: a borylene and its dimer are non-interconvertible but connected through reactivity. Chem. Sci. 2018, 9, 2252–2260. 10.1039/C7SC04789D. 29719698 PMC5897878

[ref18] Stoy A.; Arrowsmith M.; Eyßelein M.; Dellermann T.; Mies J.; Radacki K.; Kupfer T.; Braunschweig H. NHC-Stabilized 1,2-Dihalodiborenes: Synthesis, Characterization, and Reactivity Toward Elemental Chalcogens. Inorg. Chem. 2021, 60, 12625–12633. 10.1021/acs.inorgchem.1c01169. 34042444

[ref19] Fan J.; Chia P.-T.; Zhang Z.-F.; Yang M.-C.; Su M.-D.; So C.-W. A Pyridine-Stabilized N-Phosphinoamidinato N-Heterocyclic Carbene-Diboravinyl Cation: Boron Analogue of Vinyl Cation. Angew. Chem., Int. Ed. 2022, 61, e202212842 10.1002/anie.202212842. 36098906

[ref20] Wang S. R.; Arrowsmith M.; Böhnke J.; Braunschweig H.; Dellermann T.; Dewhurst R. D.; Kelch H.; Krummenacher I.; Mattock J. D.; Müssig J. H.; Thiess T.; Vargas A.; Zhang J. Engineering a Small HOMO-LUMO Gap and Intramolecular B-B Hydroarylation by Diborene/Anthracene Orbital Intercalation. Angew. Chem., Int. Ed. 2017, 56, 8009–8013. 10.1002/anie.201704063. 28493620

[ref21] Mu Y.; Dai Y.; Ruiz D. A.; Liu L. L.; Xu L.-P.; Tung C.-H.; Kong L. Aromatic 1,4,2,3-Diazadiborole Featuring an Unsymmetrical B = B Entity: A Versatile Synthon for Unusual Boron Heterocycles. Angew. Chem., Int. Ed. 2024, 63, e202405905 10.1002/anie.202405905. 38771269

[ref22] Braunschweig H.; Constantinidis P.; Dellermann T.; Ewing W. C.; Fischer I.; Hess M.; Knight F. R.; Rempel A.; Schneider C.; Ullrich S.; Vargas A.; Woollins J. D. Highly Strained Heterocycles Constructed from Boron-Boron Multiple Bonds and Heavy Chalcogens. Angew. Chem., Int. Ed. 2016, 55, 5606–5609. 10.1002/anie.201601691. 27027522

[ref23] Paetzold P.; Géret-Baumgarten L.; Boese R. Bis(trisyl)oxadiborirane. Angew. Chem., Int. Ed. Engl. 1992, 31, 1040–1042. 10.1002/anie.199210401.

[ref24] Bühl M.; Schaefer III H. F.; von Rague Schleyer P.; Boese R. On the BO Bond Length in Oxadiboriranes. Angew. Chem., Int. Ed. Engl. 1993, 32, 1154–1155. 10.1002/anie.199311541.

[ref25] Soleilhavoup M.; Bertrand G. Borylenes: An Emerging Class of Compounds. Angew. Chem., Int. Ed. 2017, 56, 10282–10292. 10.1002/anie.201705153. 28577325

[ref26] Légaré M.-A.; Pranckevicius C.; Braunschweig H. Metallomimetic Chemistry of Boron. Chem. Rev. 2019, 119, 8231–8261. 10.1021/acs.chemrev.8b00561. 30640447

[ref27] Kinjo R.; Donnadieu B.; Celik M. A.; Frenking G.; Bertrand G. Synthesis and characterization of a neutral tricoordinate organoboron isoelectronic with amines. Science 2011, 333, 610–613. 10.1126/science.1207573. 21798945

[ref28] Kong L.; Li Y.; Ganguly R.; Vidovic D.; Kinjo R. Isolation of a bis(oxazol-2-ylidene)-phenylborylene adduct and its reactivity as a boron-centered nucleophile. Angew. Chem., Int. Ed. 2014, 53, 9280–9283. 10.1002/anie.201405201. 24980138

[ref29] Arrowsmith M.; Auerhammer D.; Bertermann R.; Braunschweig H.; Celik M. A.; Erdmannsdorfer J.; Krummenacher I.; Kupfer T. From Borane to Borylene without Reduction: Ambiphilic Behavior of a Monovalent Silylisonitrile Boron Species. Angew. Chem., Int. Ed. 2017, 56, 11263–11267. 10.1002/anie.201705561. 28640395

[ref30] Ruiz D. A.; Melaimi M.; Bertrand G. An efficient synthetic route to stable bis(carbene)borylenes [(L1)(L2)BH]. Chem. Commun. 2014, 50, 7837–7839. 10.1039/C4CC03497J. 24909943

[ref31] Wang H.; Wu L.; Lin Z.; Xie Z. Transition-Metal-Like Behavior of Monovalent Boron Compounds: Reduction, Migration, and Complete Cleavage of CO at a Boron Center. Angew. Chem., Int. Ed. 2018, 57, 8708–8713. 10.1002/anie.201802643. 29575367

[ref32] Wang H.; Wu L.; Lin Z.; Xie Z. Synthesis Structure and Reactivity of a Borylene Cation [(NHSi)2B(CO)](+) Stabilized by Three Neutral Ligands. J. Am. Chem. Soc. 2017, 139, 13680–13683. 10.1021/jacs.7b08667. 28885010

[ref33] Braunschweig H.; Krummenacher I.; Legare M. A.; Matler A.; Radacki K.; Ye Q. Main-Group Metallomimetics: Transition Metal-like Photolytic CO Substitution at Boron. J. Am. Chem. Soc. 2017, 139, 1802–1805. 10.1021/jacs.6b13047. 28103028

[ref34] Fan J.; Koh A.-P.; Wu C.-S.; Su M.-D.; So C.-W. Carbon dioxide capture and functionalization by bis(N-heterocyclic carbene)-borylene complexes. Nat. Commun. 2024, 15, 3052 10.1038/s41467-024-47381-7. 38594261 PMC11003992

[ref35] Kong L.; Ganguly R.; Li Y.; Kinjo R. Diverse reactivity of a tricoordinate organoboron L2PhB: (L = oxazol-2-ylidene) towards alkali metal, group 9 metal, and coinage metal precursors. Chem. Sci. 2015, 6, 2893–2902. 10.1039/C5SC00404G. 29308167 PMC5655907

[ref36] Braunschweig H.; Dewhurst R. D.; Pentecost L.; Radacki K.; Vargas A.; Ye Q. Dative Bonding between Group 13 Elements Using a Boron-Centered Lewis Base. Angew. Chem., Int. Ed. 2016, 55, 436–440. 10.1002/anie.201509289. 26768824

[ref37] Liu S.; Legare M. A.; Hofmann A.; Braunschweig H. A Boradiselenirane and a Boraditellurirane: Isolable Heavy Analogs of Dioxiranes and Dithiiranes. J. Am. Chem. Soc. 2018, 140, 11223–11226. 10.1021/jacs.8b07829. 30130957

[ref38] Dahcheh F.; Martin D.; Stephan D. W.; Bertrand G. Synthesis and reactivity of a CAAC-aminoborylene adduct: a hetero-allene or an organoboron isoelectronic with singlet carbenes. Angew. Chem., Int. Ed. 2014, 53, 13159–13163. 10.1002/anie.201408371. 25267591

[ref39] Légaré M. A.; Bélanger-Chabot G.; Dewhurst R. D.; Welz E.; Krummenacher I.; Engels B.; Braunschweig H. Nitrogen fixation and reduction at boron. Science 2018, 359, 896–900. 10.1126/science.aaq1684. 29472479

[ref40] Legare M. A.; Belanger-Chabot G.; Rang M.; Dewhurst R. D.; Krummenacher I.; Bertermann R.; Braunschweig H. One-pot, room-temperature conversion of dinitrogen to ammonium chloride at a main-group element. Nat. Chem. 2020, 12, 1076–1080. 10.1038/s41557-020-0520-6. 32929247

[ref41] Légaré M.-A.; Rang M.; Bélanger-Chabot G.; Schweizer J. I.; Krummenacher I.; Bertermann R.; Arrowsmith M.; Holthausen M. C.; Braunschweig H. The reductive coupling of dinitrogen. Science 2019, 363, 1329–1332. 10.1126/science.aav9593. 30898929

[ref42] Rang M.; Heinz M.; Halkić A.; Weber M.; Dewhurst R. D.; Rempel A.; Härterich M.; Holthausen M. C.; Braunschweig H. Trapping of a Terminal Intermediate in the Boron-Mediated Dinitrogen Reduction: Mono-Tri-and Tetrafunctionalized Hydrazines in Two Steps from N2. J. Am. Chem. Soc. 2024, 146, 11048–11053. 10.1021/jacs.4c01818. 38598273

[ref43] Zhou Y. P.; Driess M. Isolable Silylene Ligands Can Boost Efficiencies and Selectivities in Metal-Mediated Catalysis. Angew. Chem., Int. Ed. 2019, 58, 3715–3728. 10.1002/anie.201811088. 30318857

[ref44] Yao S.; Saddington A.; Xiong Y.; Driess M. Chelating Bis-silylenes As Powerful Ligands To Enable Unusual Low-Valent Main-Group Element Functions. Acc. Chem. Res. 2023, 56, 475–488. 10.1021/acs.accounts.2c00763. 36720115

[ref45] Kern R. H.; Schneider M.; Eichele K.; Schubert H.; Bettinger H. F.; Wesemann L. Boradigermaallyl: (4 + 3) Cycloaddition-Initiated Boron Insertion into Benzene. Angew. Chem., Int. Ed. 2023, 62, e202301593 10.1002/anie.202301593. 36807732

[ref46] Chen M.; Zhang Z.; Qiao Z.; Zhao L.; Mo Z. An Isolable Bis(Germylene)-Stabilized Plumbylone. Angew. Chem., Int. Ed. 2023, 62, e202215146 10.1002/anie.202215146. 36421062

[ref47] Liu T.-T.; Chen J.; Chen X.-L.; Ma L.; Guan B.-T.; Lin Z.; Shi Z.-J. Neutral Boryl Radicals in Mixed-Valent B(III)Br-B(II) Adducts. Chem.—Eur. J. 2023, 29, e202202634 10.1002/chem.202202634. 36217568

[ref48] Bissinger P.; Braunschweig H.; Damme A.; Dewhurst R. D.; Kupfer T.; Radacki K.; Wagner K. Generation of a carbene-stabilized bora-borylene and its insertion into a C-H bond. J. Am. Chem. Soc. 2011, 133, 19044–19047. 10.1021/ja208372k. 22032808

[ref49] Fan J.; Xu J.; Ma Q.; Yao S.; Zhao L.; Frenking G.; Driess M. Silylene-Stabilized Neutral Dibora-Aromatics with a B = B Bond. J. Am. Chem. Soc. 2024, 146, 20458–20467. 10.1021/jacs.4c06579. 38980827 PMC11273343

[ref50] Gärtner A.; Meier L.; Arrowsmith M.; Dietz M.; Krummenacher I.; Bertermann R.; Fantuzzi F.; Braunschweig H. Highly Strained Arene-Fused 1,2-Diborete Biradicaloid. J. Am. Chem. Soc. 2022, 144, 21363–21370. 10.1021/jacs.2c09971. 36350352

[ref51] Wang J.; Ye Q. Borirenes and Boriranes: Development and Perspectives. Chem.—Eur. J. 2024, 30, e202303695 10.1002/chem.202303695. 38085103

[ref52] Wang H.; Zhang J.; Xie Z. Ring-opening and ring-expansion reactions of carborane-fused borirane. Chem. Sci. 2021, 12, 13187–13192. 10.1039/D1SC04453B. 34745550 PMC8513813

[ref53] Zhu L.; Kinjo R. Crystalline 2π Aromatic Azadiboriridinylium: A BN Analogue of Cyclopropenylium Cation. Angew. Chem., Int. Ed. 2023, 62, e202312949 10.1002/anie.202312949. 37828652

[ref54] Braunschweig H.; Radacki K.; Schneider A. Cyclodimerization of an Oxoboryl Complex Induced by trans Ligand Abstraction. Angew. Chem., Int. Ed. 2010, 49, 5993–5996. 10.1002/anie.201002300. 20623815

[ref55] Romero E.; Gómez Castellanos J. R.; Gadda G.; Fraaije M. W.; Mattevi A. Same Substrate, Many Reactions: Oxygen Activation in Flavoenzymes. Chem. Rev. 2018, 118, 1742–1769. 10.1021/acs.chemrev.7b00650. 29323892

[ref56] Tao X.; Daniliuc C. G.; Janka O.; Pöttgen R.; Knitsch R.; Hansen M. R.; Eckert H.; Lübbesmeyer M.; Studer A.; Kehr G.; Erker G. Reduction of Dioxygen by Radical/B(p-C6F4X)3 Pairs to Give Isolable Bis(borane)superoxide Compounds. Angew. Chem., Int. Ed. 2017, 56, 16641–16644. 10.1002/anie.201709309. 29112325

[ref57] Wood T. K.; Piers W. E.; Keay B. A.; Parvez M. 9-Boraanthracene Derivatives Stabilized by N-Heterocyclic Carbenes. Angew. Chem., Int. Ed. 2009, 48, 4009–4012. 10.1002/anie.200901217. 19402082

[ref58] Tsurumaki E.; Sung J.; Kim D.; Osuka A. Stable Boron Peroxides with a Subporphyrinato Ligand. Angew. Chem., Int. Ed. 2016, 55, 2596–2599. 10.1002/anie.201511590. 26773709

[ref59] Henthorn J. T.; Agapie T. Dioxygen Reactivity with a Ferrocene–Lewis Acid Pairing: Reduction to a Boron Peroxide in the Presence of Tris(pentafluorophenyl)borane. Angew. Chem., Int. Ed. 2014, 53, 12893–12896. 10.1002/anie.201408462. 25250531

[ref60] Wang B.; Kinjo R. Boron-based stepwise dioxygen activation with 1,4,2,5-diazadiborinine. Chem. Sci. 2019, 10, 2088–2092. 10.1039/C8SC04624G. 30881633 PMC6385103

[ref61] Taylor J. W.; McSkimming A.; Guzman C. F.; Harman W. H. N-Heterocyclic Carbene-Stabilized Boranthrene as a Metal-Free Platform for the Activation of Small Molecules. J. Am. Chem. Soc. 2017, 139, 11032–11035. 10.1021/jacs.7b06772. 28759220

[ref62] Fan J.; Yang M.-C.; Su M.-D.; So C.-W. N-Phosphinoamidinato Silylene– and Phosphine–Borylborylene Complexes. Inorg. Chem. 2023, 62, 863–870. 10.1021/acs.inorgchem.2c03660. 36600552

[ref63] Lu W.; Li Y.; Ganguly R.; Kinjo R. Alkene-Carbene Isomerization induced by Borane: Access to an Asymmetrical Diborene. J. Am. Chem. Soc. 2017, 139, 5047–5050. 10.1021/jacs.7b02251. 28334527

[ref64] Katsuma Y.; Tsukahara N.; Wu L.; Lin Z.; Yamashita M. Reaction of B_2_ (o-tol)_4_ with CO and Isocyanides: Cleavage of the C-O Triple Bond and Direct C-H Borylations. Angew. Chem., Int. Ed. 2018, 57, 6109–6114. 10.1002/anie.201800878. 29573087

