# Supplementary material for: From Bis(borylene)-Substituted Xanthenes as Reactive Intermediates to Diboraoxirane Complexes
Source: J Am Chem Soc. 2025 Feb 13;147(8):6925–33. doi: 10.1021/jacs.4c17463 (PMC11869280; doi:10.1021/jacs.4c17463)
Supplement: Supplementary file 1 — ja4c17463_si_001.pdf [file ja4c17463_si_001.pdf]

Supporting Information

From Bis(borylene)-Substituted Xanthenes as Reactive Intermediates to Diboraoxirane Complexes

Jun Fan,[a] Sudip Pan,[b] Shenglai Yao,[a] Chengxiang Ding,[b] Gernot Frenking*[c, d] and Matthias Driess*[a]

[a]Department of Chemistry: Metalorganics and Inorganic Materials, Technische Universität Berlin, Strasse des 17. Juni 115, Sekr. C2, 10623 Berlin (Germany)

[b]Institute of Atomic and Molecular Physics, Jilin University, 130023 Changchun (China)

[c]State Key Laboratory of Materials-Oriented Chemical Engineering, School of Chemistry and Molecular Engineering, Nanjing Tech University, Nanjing 211816 (China)

[d]Fachbereich Chemie, Philipps-Universität Marburg, 35032 Marburg (Germany)

Contents

A. Experimental Procedures 2

A1. General Considerations 2

A2. Single-Crystal X-ray Structure Determination 2

B. Synthesis and Characterization 3

C. X-ray Crystallographic Data 42

D. Theoretical Calculations 66

References 109

A. Experimental Procedures

A1. General Considerations

All experiments were carried out under dry oxygen-free nitrogen using standard Schlenk techniques or MBraun glove box fitted with a gas purification and recirculation unit. Solvents were dried by standard methods and freshly distilled prior to use. The solution NMR spectra were recorded on Bruker Spectrometers AV 400 and 500 with residual solvent signals as internal reference (^{1}H NMR: Benzene-*d*$_6$, 7.16 ppm, THF-*d*$_8$, 3.58 and 1.72 ppm, CD_2Cl_2, 5.32 ppm, Pyridine-*d*$_5$, 8.74, 7.58 and 7.22 ppm, ^{13}C{^{1}H} NMR: Benzene-*d*$_6$, 128.06 ppm, THF-*d*$_8$, 67.21 and 25.31 ppm, CD_2Cl_2, 53.84 ppm, Pyridine-*d*$_5$, 123.5 ppm) and external standards (^{1}H and ^{13}C{^{1}H} NMR: $SiMe_4$; ^{11}B{^{1}H} NMR: $BF_3.Et_2O$). The following abbreviations were used to describe peak patterns when appropriate: br = broad, s = singlet, d = doublet, t = triplet, dd = doublet of doublets, m = multiplet. High-resolution ESI-MS were measured on a Thermo Scientific LTQ orbitrap XL. FT-IR spectra were measured with a Nicolet iS5 FT-IR-Spectrometer from the company Thermo. UV/Vis spectra were recorded on an Analytik Jena Specord S600 diode array spectrometer.

A2. Single-Crystal X-ray Structure Determination

Crystals were each mounted on a glass capillary in perfluorinated oil and measured in a cold N_2 flow. The data of all compounds were collected on an Oxford Diffraction SuperNova, Single source at offset, Atlas at 130 or 150 K (Cu-Kα radiation, λ = 1.54184 Å). The structures were solved by direct methods and refined on F^2 with the SHELX-2014[1] and Olex2[2] software package. For the crystal of compounds **1**, **2**, **7** and **12**, strongly disordered solvent molecules THF (**1**, **2**, **12**), and C_7H_8 (**7**) were treated using Solvent Masking in Olex2. In the molecular structure of compound **4**, the entire molecule is disordered over two orientations with a ratio of 0.32:0.68. For the crystal of compounds **4** and **5**, despite multiple recrystallizations of these compounds, significant molecular disorder persists. As a result, the data are sufficient solely for structure confirmation, and the selected bond lengths and bond angles should be considered for reference purposes only.

CCDC: 2390958 (**1**), 2390956 (**2**), 2390955 (**3**), 2390951 (**4**), 2390948 (**5**), 2390953 (**6**), 2390949 (**7**), 2390950 (**8**), 2390947 (**9**), 2390957 (**10**), 2390954 (**11**) and 2390952 (**12**) contain the supplementary crystallographic data for this paper. These data can be obtained free of charge from The Cambridge Crystallographic Data Centre via www.ccdc.cam.ac.uk/structures/

B. Synthesis and Characterization

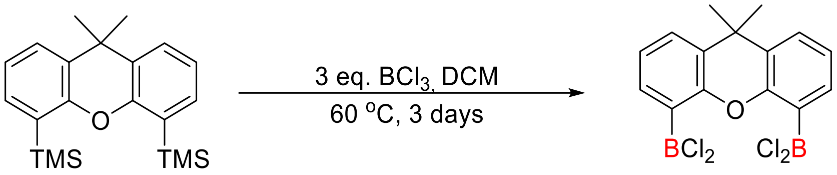

Synthesis of BCl_2(Xant)BCl_2. In a high-pressure Schlenk flask, the hexane solution of BCl_3 (1 M, 30 mL, 30 mmol) was added into a DCM solution of 4,5-bis(trimethylsilyl)xanthene (3.56 g, 10 mmol) at room temperature. The reaction mixture was stirred at 60 °C for three days. The resulting solution was dried under vacuum to remove all volatiles to get a brown solid, which was washed with cold hexane to give **BCl_2(Xant)BCl_2** as white solid in 76 % yield (2.83 g).

M.p.: 108.7 °C.

^{1}H NMR (500 MHz, Benzene-d_6) δ/ppm 7.45 (d, J = 7.3 Hz, 2H, Ar-H), 7.10 (d, J = 7.8 Hz, 2H, Ar-H), 6.75 (t, J = 7.6 Hz, 2H, Ar-H), 1.19 (s, 6H, C(CH_3)$_2$).

^{13}C{^{1}H} NMR (126 MHz, Benzene-d_6) δ/ppm 153.38, 133.95, 130.96, 130.18, 123.00 (s, Ar-C),, 33.82 (s, C(CH$_3$)$_2$, 31.06 (s, C(CH$_3$)$_2$).

^{11}B{^{1}H} NMR (160 MHz, Benzene-d_6) δ/ppm 56.26.

HR-MS (ESI): (m/z) calcd for $[M\text{-}Cl]^+$ ($C_{15}H_{12}B_2Cl_3O^+$) 335.0134; Found: 335.0726.

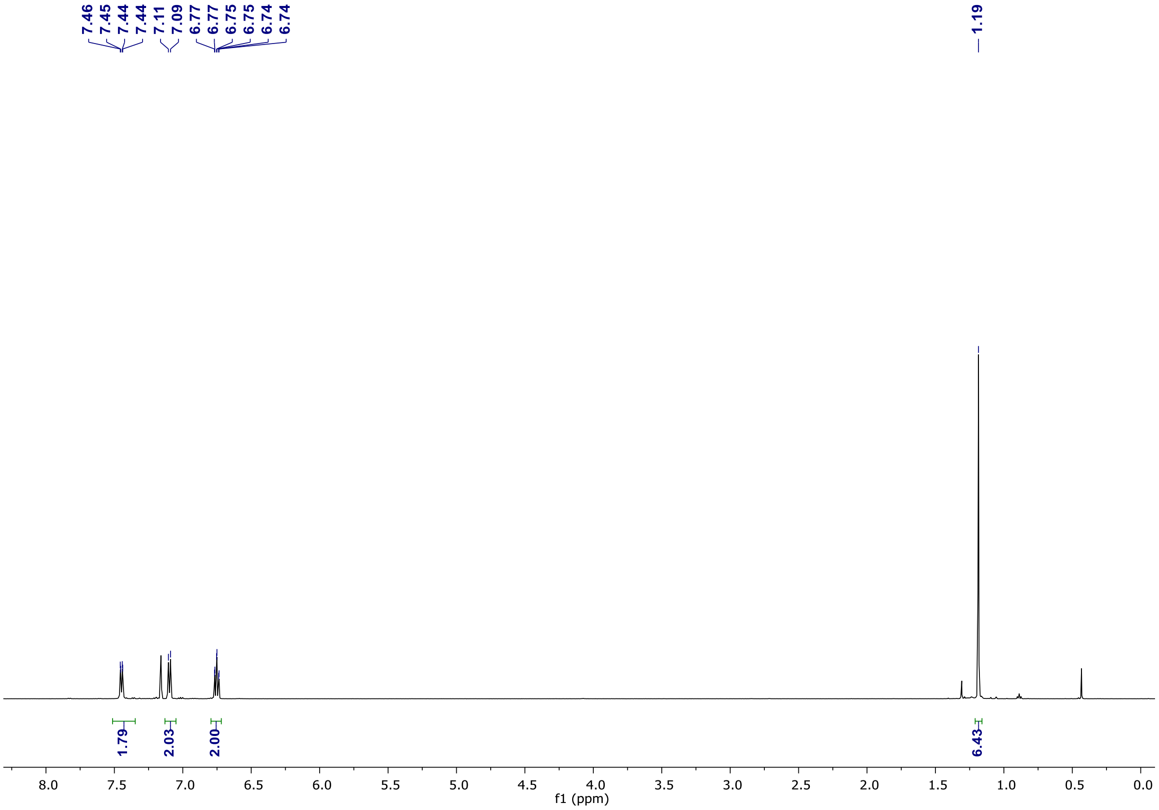

Figure S1. ^{1}H NMR spectrum of **BCl_2(Xant)BCl_2** in benzene-d_6.

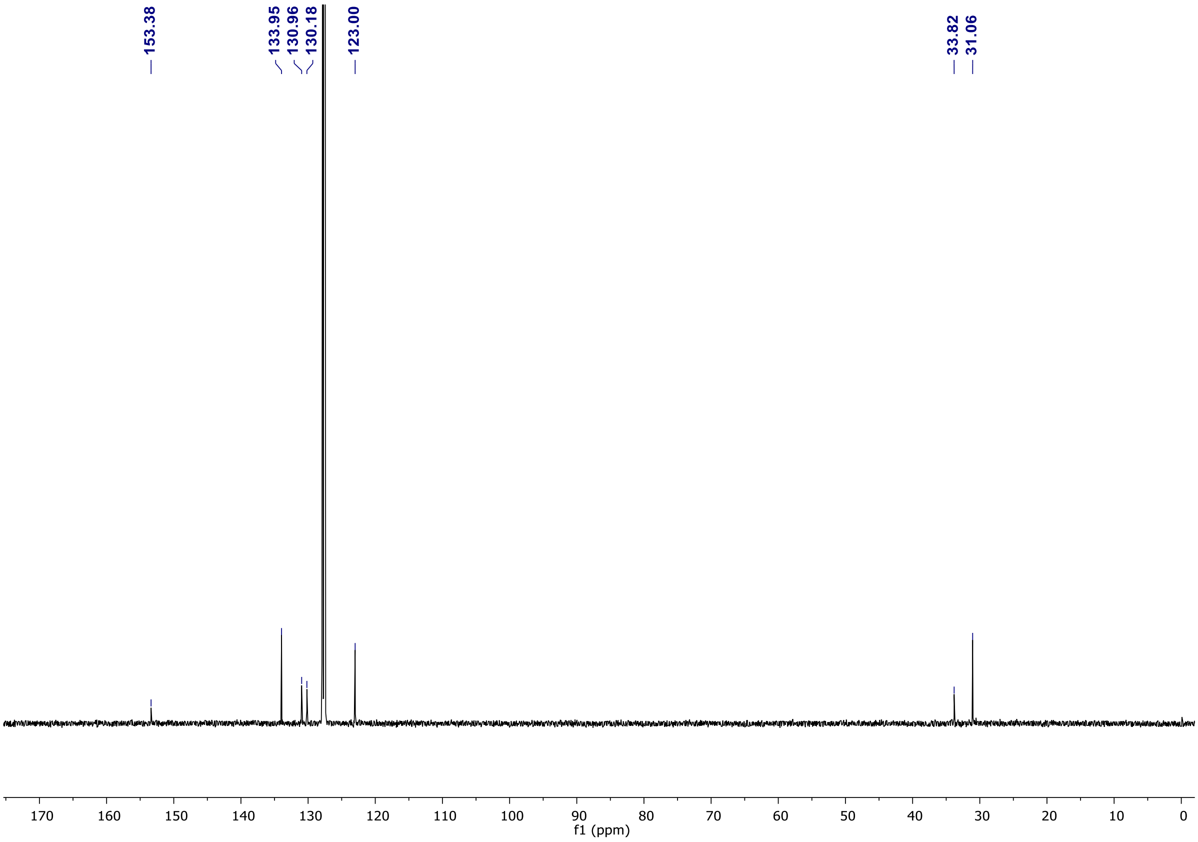

Figure S2. $^{13}C\{^{1}H\}$ NMR spectrum of **BCl_2(Xant)BCl_2** in benzene-d_6.

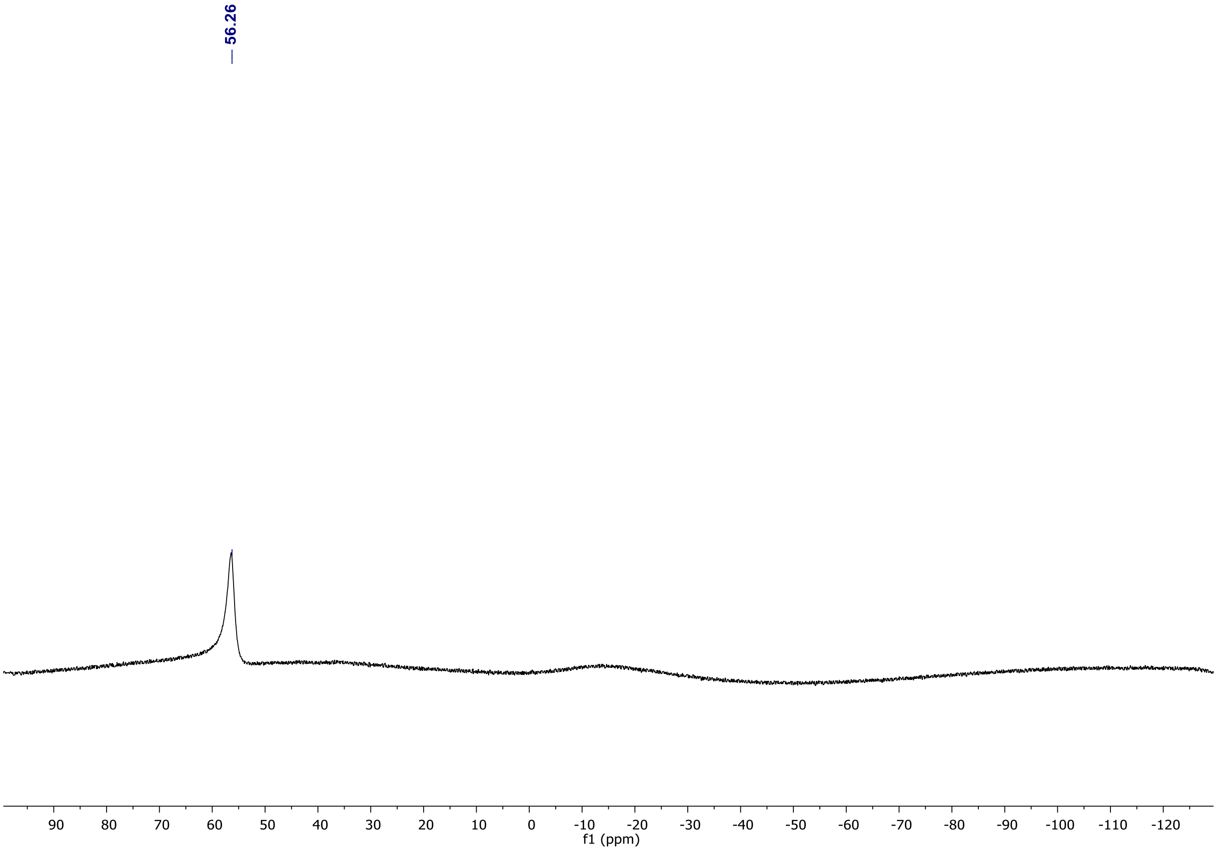

Figure S3. $^{11}B\{^{1}H\}$ NMR spectrum of **BCl_2(Xant)BCl_2** in benzene-d_6.

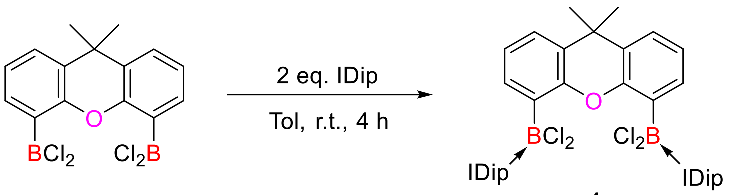

1

Synthesis of compound 1. Toluene (60 mL) was added into a 100 mL Schlenk flask containing **BCl_2(Xant)BCl_2** (372 mg, 1 mmol) and IDip (776 mg, 2 mmol) at room temperature. The reaction mixture was stirred at room temperature for 4 hours. The resulting solution was filtered. The filtrate was concentrated to 5 mL to afford compound **1** as colorless crystals in 94 % yield (1081 mg).

M.p.: 197.6 °C.

^{1}H NMR (500 MHz, THF-d_8) δ/ppm 7.40 (s, 4H, C*H*=C*H*), 7.23 (t, J = 7.8 Hz, 4H, Ar-*H*), 7.08 (d, J = 7.8 Hz, 8H, Ar-*H*), 6.89 (d, J = 7.6 Hz, 2H, Ar-*H*), 6.77 (d, J = 7.4 Hz, 2H, Ar-*H*), 6.33 (t, J = 7.5 Hz, 2H, Ar-*H*), 2.92 (sept, J = 6.7 Hz, 8H, C*H*(CH_3)$_2$), 1.39 (s, 6H, C(CH_3)$_2$), 1.15 (d, J = 6.7 Hz, 24H, CH(CH_3)$_2$), 1.03 (d, J = 6.9 Hz, 24H, CH(CH_3)$_2$).

^{13}C{^{1}H} NMR (126 MHz, THF-d_8) δ/ppm 154.34, 146.35, 137.32, 135.90, 130.18, 128.62, 126.24, 124.93, 123.97 (s, Ar-*C*), 119.87 (s, *C*H=*C*H), 34.57 (s, *C*(CH_3)$_2$), 34.32 (s, C(*C*H_3)$_2$), 29.86 (s, CH(*C*H_3)$_2$), 26.00 (s, CH(*C*H_3)$_2$), 23.33 (s, *C*H(CH_3)$_2$).

^{11}B{^{1}H} NMR (160 MHz, THF-d_8) δ/ppm 1.34.

HR-MS (ESI): (m/z) calcd for $[M-Cl]^+$ ($C_{69}H_{84}B_2N_4O_1Cl_3^+$) 1110.5928; Found: 1110.6362.

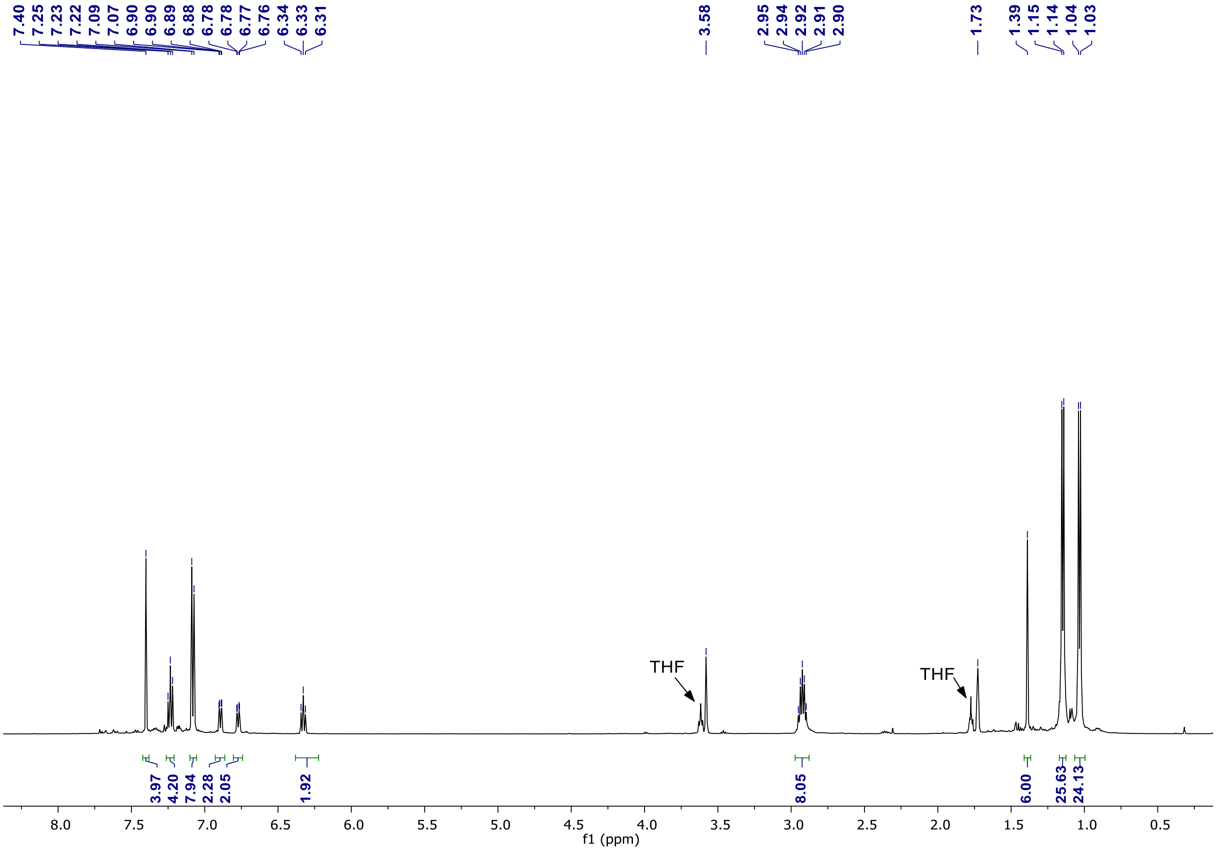

Figure S4. ^{1}H NMR spectrum of **1** in THF-d_8.

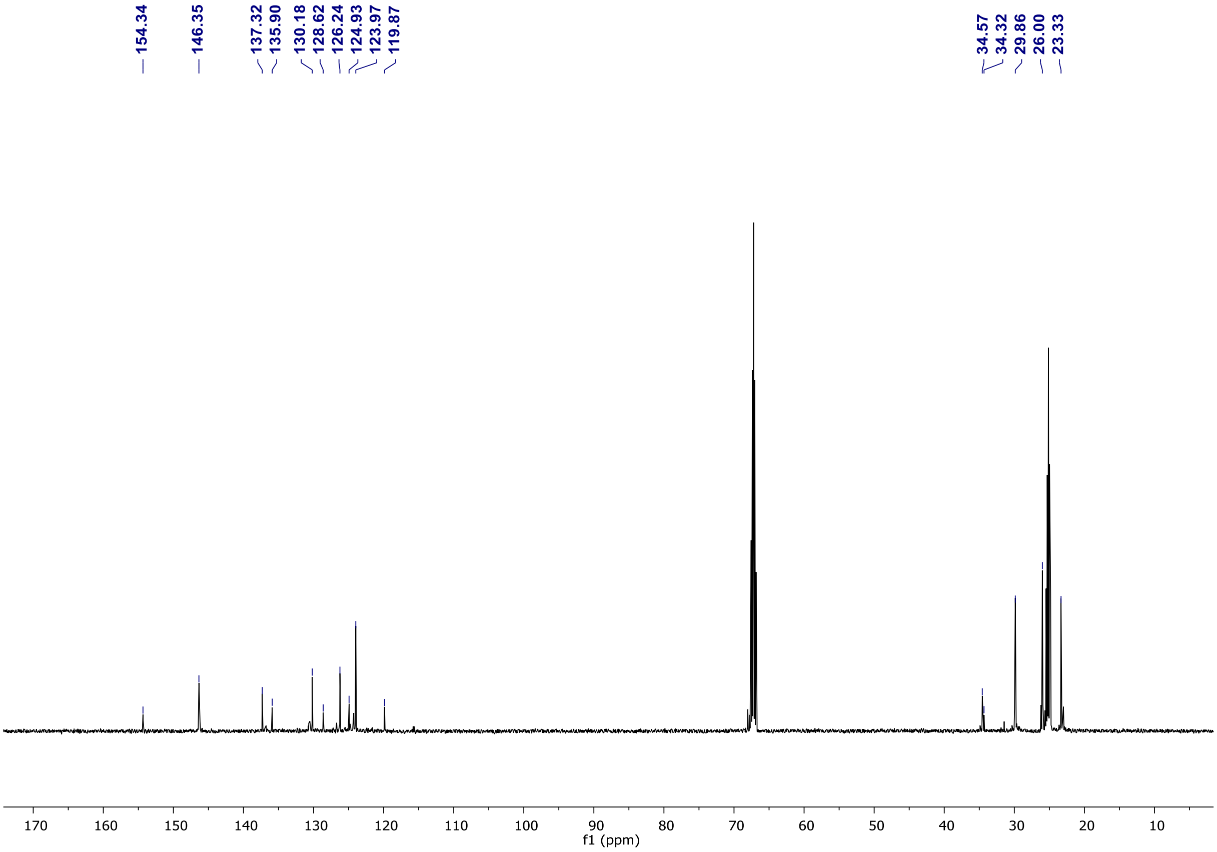

Figure S5. ^{13}C{^{1}H} NMR spectrum of **1** in THF-d_8.

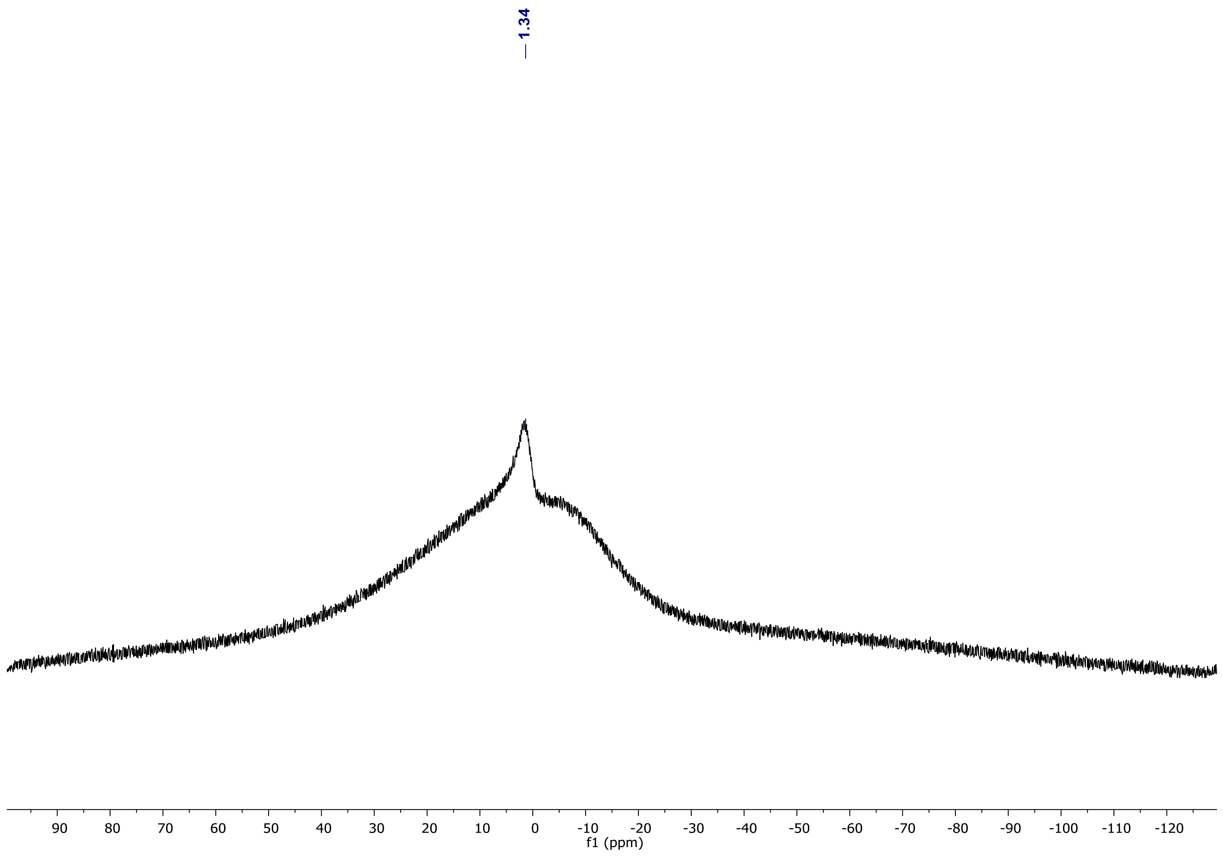

Figure S6. $^{11}B\{^{1}H\}$ NMR spectrum of **1** in THF-d_8.

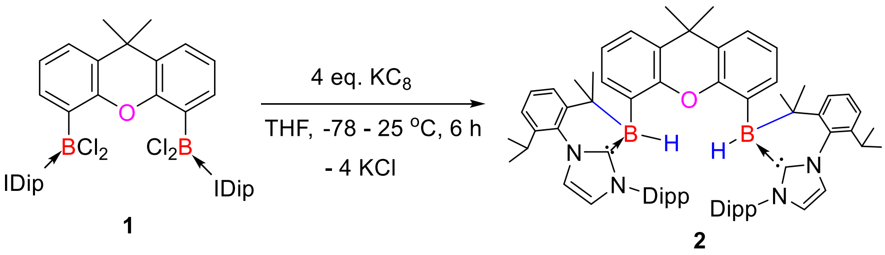

Synthesis of compound 2. THF (50 mL) was added into a 100 mL Schlenk flask containing **1** (1148 mg, 1 mmol) and KC_8 (540 mg, 4 mmol) at -78 °C under stirring. The reaction mixture tured reddish-purple immediately. The mixture was allowed to warm up to room temperature during which time another color change from reddish-purple to yellow was observed. After 6 hours, the resulting yellow suspension was filtered and the filtrare was concentrated to afford compound **2** as colorless crystals in 91 % yield (919 mg).

M.p.: 263.6 °C.

^{1}H NMR (500 MHz, THF-d_8) δ/ppm 7.70 (d, J = 2.0 Hz, 2H, Ar-H), 7.40 (t, J = 7.8 Hz, 2H, Ar-H), 7.32 (m, 2H, Ar-H), 7.28 (d, J = 7.8 Hz, 2H, Ar-H), 7.15 (d, J = 7.9 Hz, 2H, Ar-H), 7.07 (m, 4H, Ar-H), 6.90 (dd, J = 7.5, 1.4 Hz, 2H, Ar-H), 6.74 (dd, J = 7.0, 2.4 Hz, 2H, Ar-H), 6.26 – 6.19 (m, 4H, CH=CH), 3.72 – 3.61 (sept, J = 6.8 Hz, 2H, CH(CH$_3$)$_2$), 2.67 – 2.57 (sept, J = 6.8 Hz, 2H, CH(CH$_3$)$_2$), 2.57 – 2.49 (sept, J = 6.8 Hz, 2H, CH(CH$_3$)$_2$), 1.59 (d, J = 6.7 Hz, 6H, CH(CH_3)$_2$), 1.31 – 1.26 (12H, CH(CH_3)$_2$; 6H, C(CH_3)$_2$, overlapping), 1.22 (d, J = 6.8 Hz, 6H, CH(CH_3)$_2$), 0.76 (d, J = 6.9 Hz, 6H, CH(CH_3)$_2$), 0.69 (s, 12H, CB(CH_3)$_2$), 0.28 (d, J = 6.7 Hz, 6H, CH(CH_3)$_2$).

^{13}C{^{1}H} NMR (126 MHz, THF-d_8) δ/ppm 155.69, 150.28, 147.07, 145.50, 138.22, 134.72, 134.19, 132.27, 130.25, 128.17, 124.94, 124.49, 124.41, 123.00, 122.55, 121.62 (s, Ar-C), 120.59 (s, CH=CH), 119.95 (s, CH=CH), 34.53 (s, C(CH$_3$)$_2$), 33.34 (s, C(CH$_3$)$_2$), 32.44 (s, CB(CH$_3$)$_2$), 29.13 (s, CH(CH$_3$)$_2$), 29.08 (s, CH(CH$_3$)$_2$), 28.72 (s, CH(CH$_3$)$_2$), 25.86 (s, CH(CH$_3$)$_2$), 24.18 (s, CH(CH$_3$)$_2$), 21.15 (s, CB(CH$_3$)$_2$).

^{11}B{^{1}H} NMR (160 MHz, THF-d_8) δ/ppm -18.58.

IR (cm^{-1}): 2367.39 (br, BH).

HR-MS (ESI): (m/z) calcd for [M+H]$^+$ ($C_{69}H_{83}B_2N_4O_1{}^+$) 1005.6748; Found: 1005.6775.

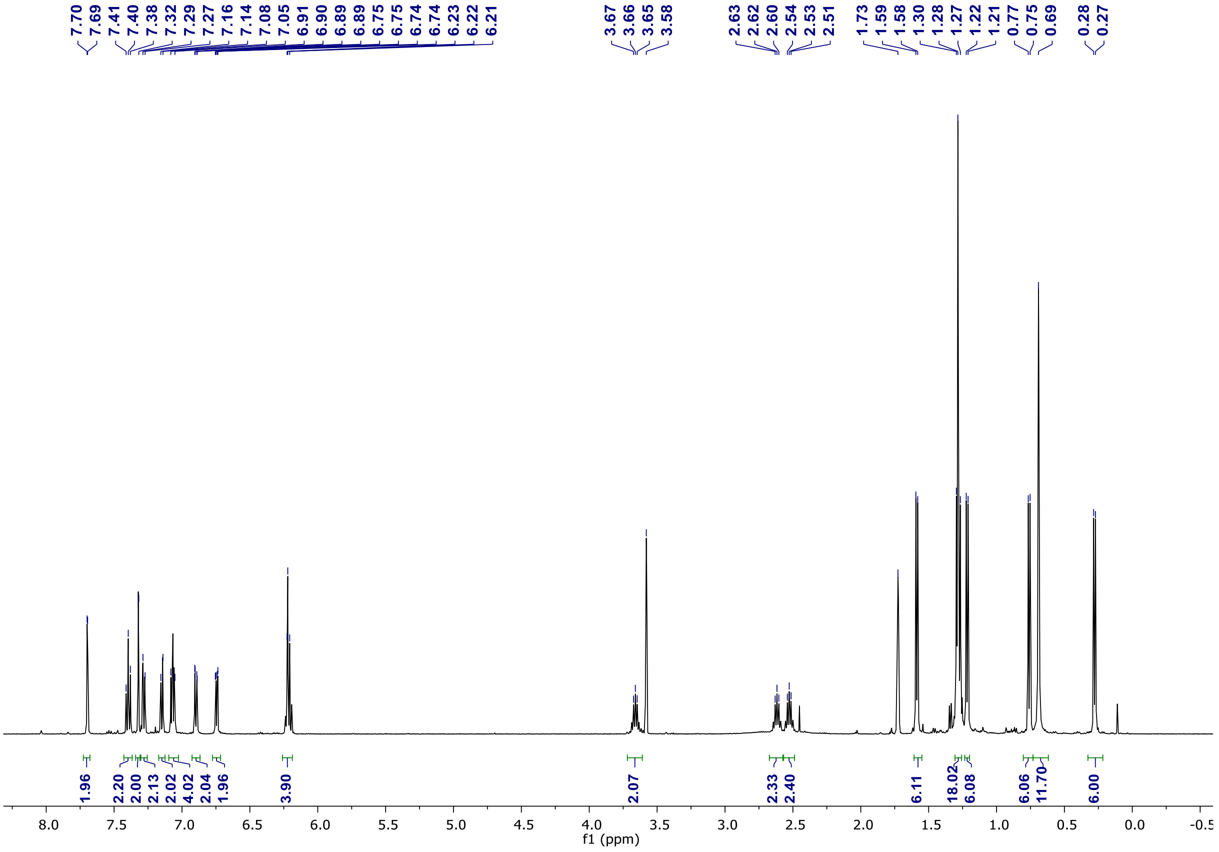

Figure S7. ^{1}H NMR spectrum of **2** in THF-d_8.

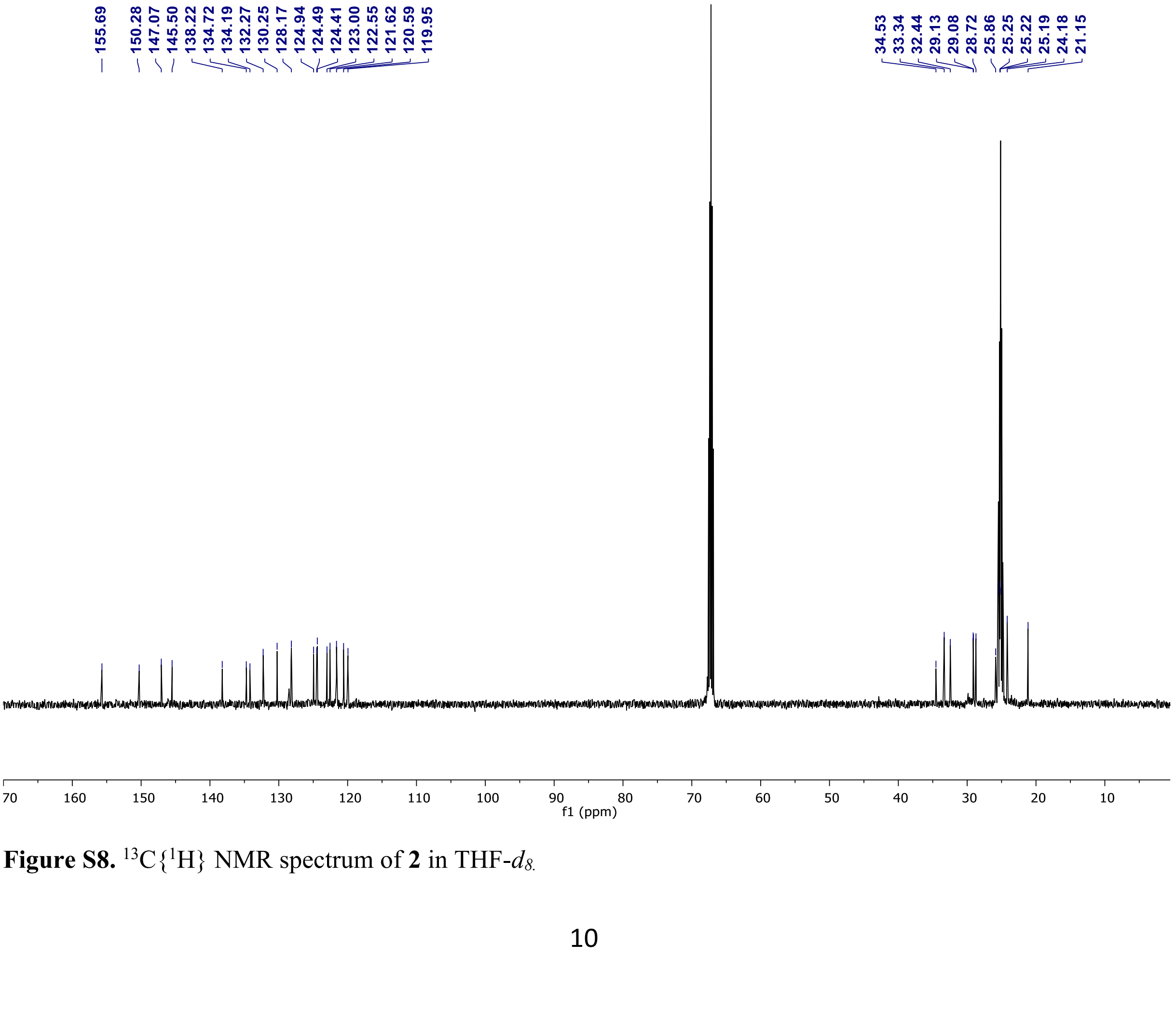

Figure S8. ^{13}C{^{1}H} NMR spectrum of **2** in THF-d_8.

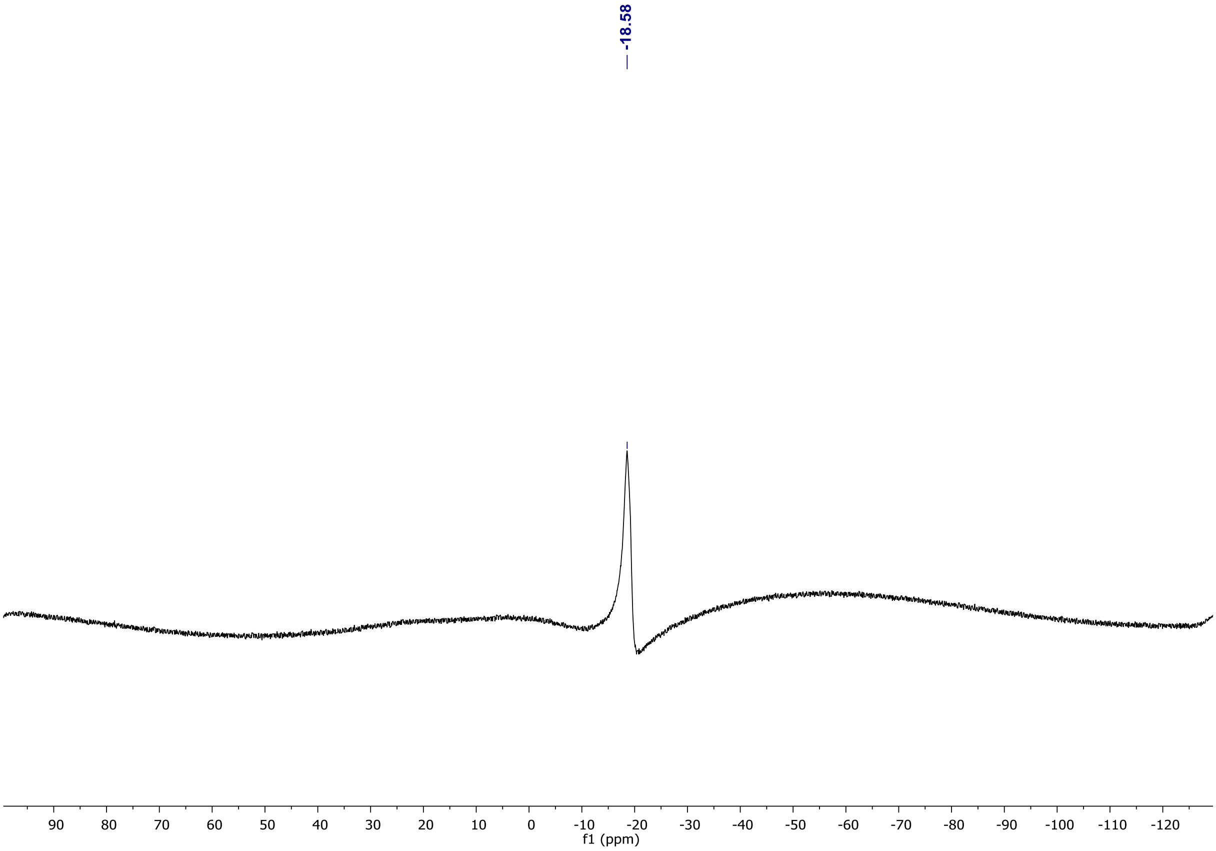

Figure S9. $^{11}B\{^{1}H\}$ NMR spectrum of **2** in THF-d_8.

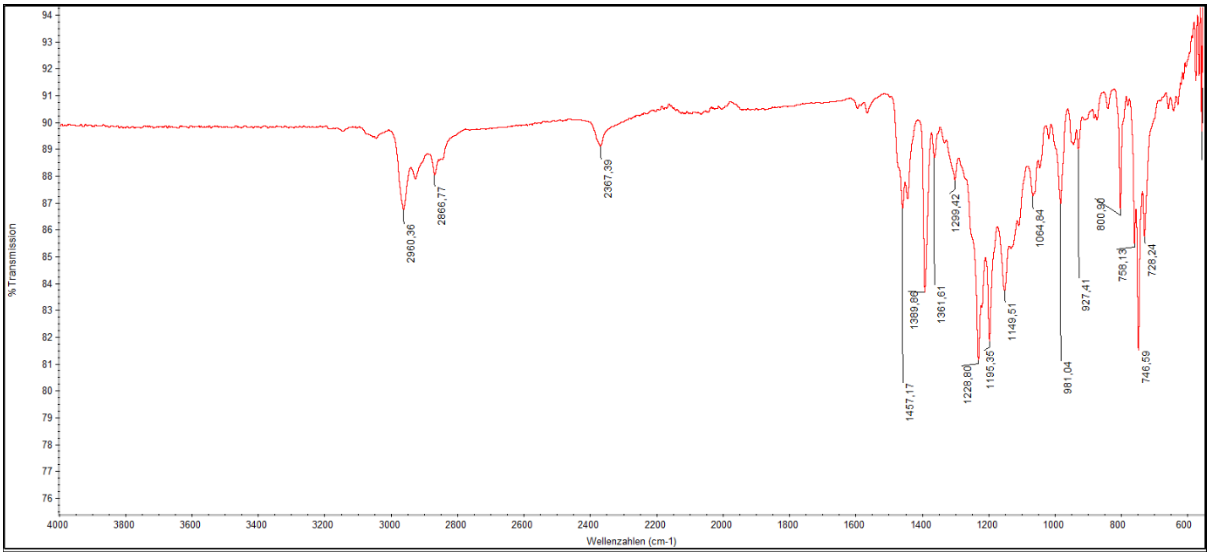

Figure S10. IR spectrum of **2**.

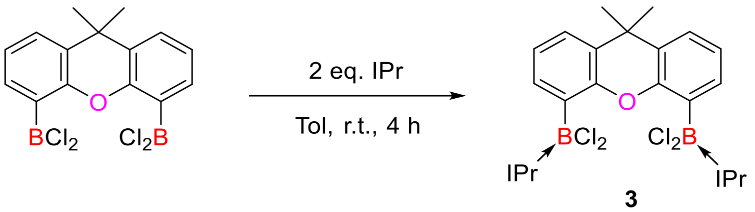

3

Synthesis of compound 3. Toluene (60 mL) was added into a 100 mL Schlenk flask containing **BCl_2(Xant)BCl_2** (372 mg, 1 mmol) and IPr (360 mg, 2 mmol) at room temperature. The reaction mixture was stirred at room temperature for 4 hours. The resulting solution was filtered. The filtrate was concentrated to afford compound **3** as colorless crystals in 89% yield (652 mg).

M.p.: 228.9 °C.

^{1}H NMR (500 MHz, THF-d_8) δ/ppm 7.48 (d, J = 7.5 Hz, 2H, Ar-H), 7.28 (d, J = 7.6 Hz, 2H, Ar-H), 6.84 (t, J = 7.6 Hz, 2H, Ar-H), 6.07 – 5.85 (sept, J = 7.0 Hz, 4H, CH(CH$_3$)$_2$), 2.26 (s, 12H, CH_3C=CCH_3), 1.60 (s, 6H, C(CH_3)$_2$), 1.30 (d, J = 7.0 Hz, 24H, CH(CH_3)$_2$).

^{13}C{^{1}H} NMR (126 MHz, THF-d_8) δ/ppm 153.71, 134.70, 130.17, 126.66, 125.06 (s, Ar-C), 121.81 (s, CH$_3$$C$=$CCH_3$), 50.27 (s, CH(CH$_3$)$_2$), 34.73 (s, C(CH$_3$)$_2$), 33.73 (s, C(CH$_3$)$_2$), 21.22 (s, CH(CH$_3$)$_2$), 10.69 (s, CH$_3$C=CCH$_3$).

^{11}B{^{1}H} NMR (160 MHz, THF-d_8) δ/ppm -0.93.

HR-MS (ESI): (m/z) calcd for [M-Cl]$^+$ ($C_{37}H_{52}B_2N_4O_1Cl_3^+$) 695.3387; Found: 695.3841.

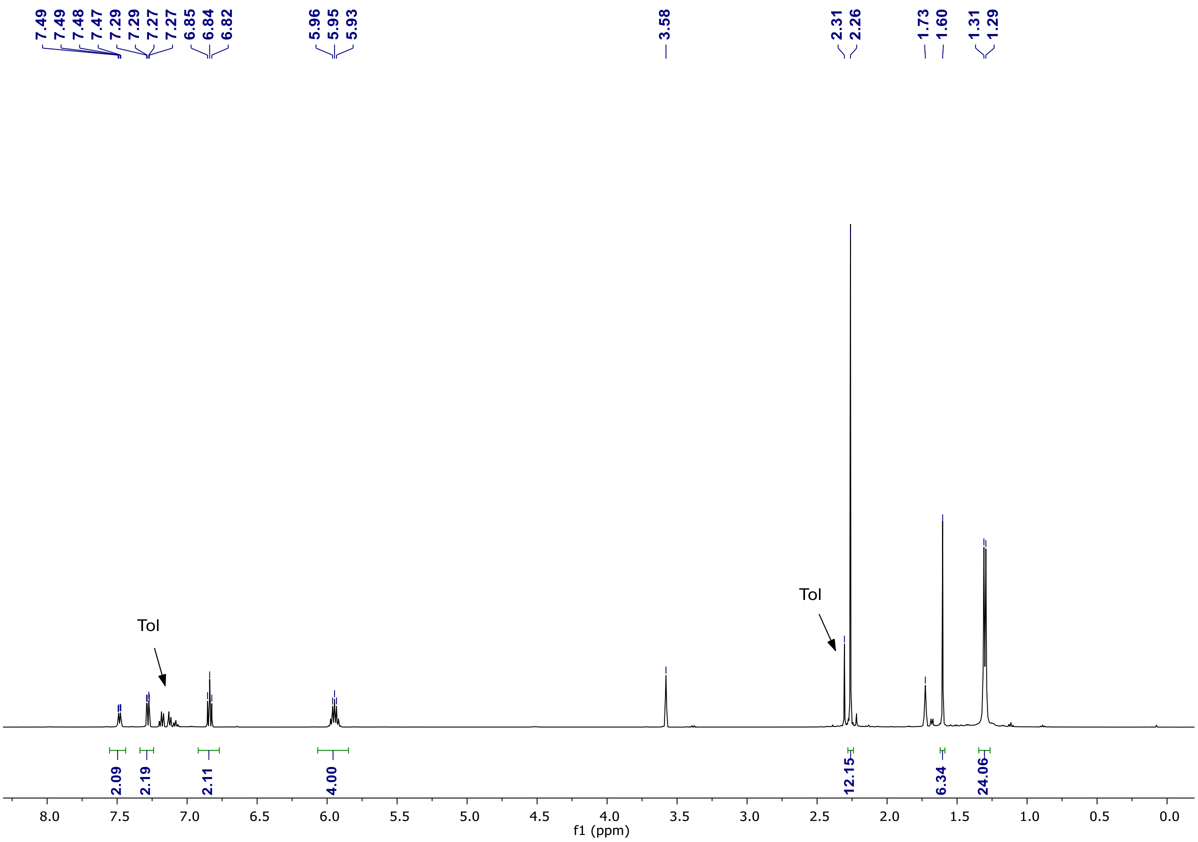

Figure S11. ^{1}H NMR spectrum of **3** in THF-d_8.

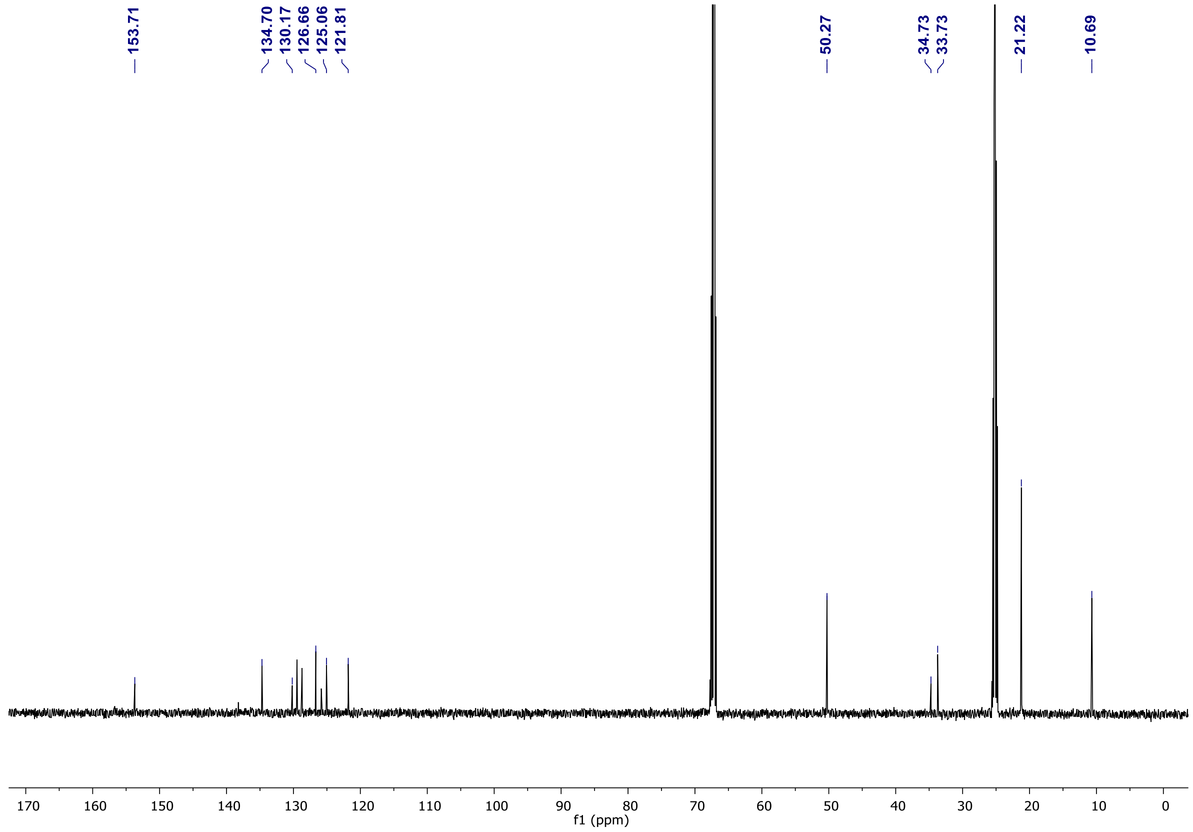

Figure S12. $^{13}C\{^{1}H\}$ NMR spectrum of **3** in THF-d_8.

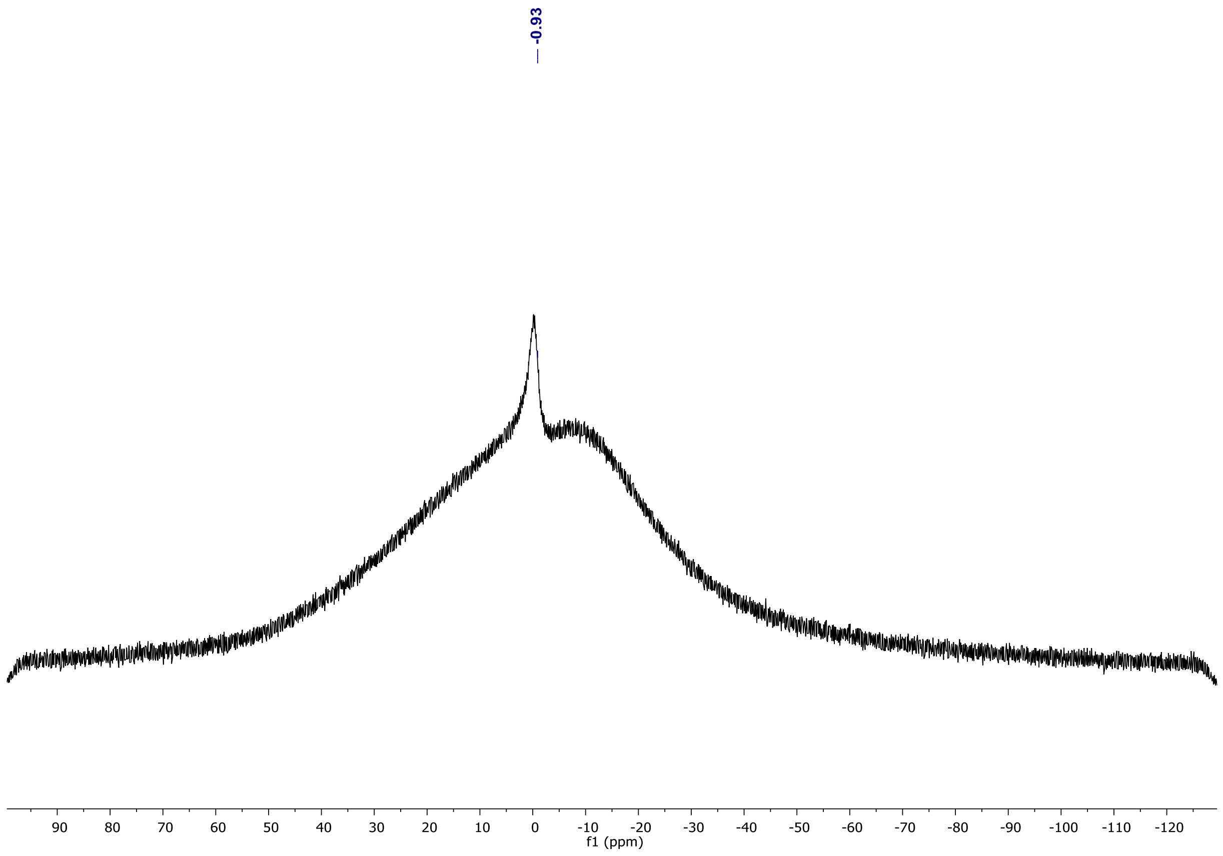

Figure S13. $^{11}B\{^{1}H\}$ NMR spectrum of **3** in THF-d_8.

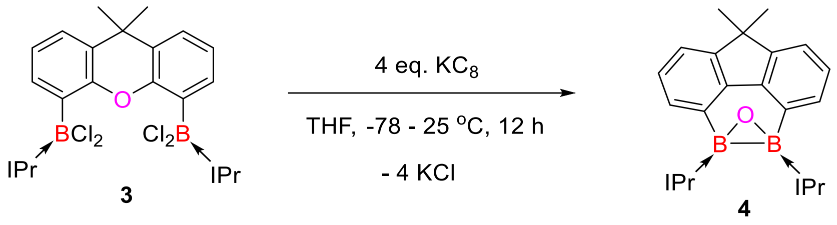

Synthesis of compound 4. THF (50 mL) was added into a 100 mL Schlenk flask containing **3** (732 mg, 1 mmol) and KC_8 (540 mg, 4 mmol) at -78 °C under stirring. The reaction mixture turned reddish-purple immediately. The mixture was allowed to warm up to room temperature during which time another color change from reddish-purple to orange-red was observed. After 12 h, the resulting orange-red suspension was filtered and the filtrare was concentrated to afford compound **4** red crystals in 56% yield (410 mg).

M.p.: 223.2 °C.

^{1}H NMR (500 MHz, Pyridine-d_5) δ/ppm 7.57 (d, J = 7.3 Hz, 2H, Ar-*H*), 7.44 (t, J = 7.2 Hz, 2H, Ar-*H*), 7.37 (d, J = 7.0 Hz, 2H, Ar-*H*), 6.61 (sept, J = 6.8 Hz, 2H, C*H*(CH_3)$_2$), 5.78 (sept, J = 6.8 Hz, 2H, C*H*(CH_3)$_2$), 2.16 (s, 12H, CH_3C=CCH_3), 1.56 (s, 3H, C(CH_3)$_2$), 1.52 (s, 3H, C(CH_3)$_2$), 1.38 (d, J = 7.0 Hz, 6H, CH(CH_3)$_2$), 1.24 (d, J = 7.1 Hz, 6H, CH(CH_3)$_2$), 1.17 (d, J = 7.0 Hz, 6H, CH(CH_3)$_2$), 0.82 (d, J = 7.0 Hz, 6H, CH(CH_3)$_2$).

^{13}C{^{1}H} NMR (126 MHz, Pyridine-d_5) δ/ppm 154.06, 145.08, 130.81, 125.48, 124.11 (s, Ar-*C*), 115.66 (s, CH_3*C*=*C*CH_3), 51.03 (s, *C*H(CH_3)$_2$), 50.00 (s, *C*H(CH_3)$_2$), 48.84 (s, *C*(CH_3)$_2$), 30.29 (s, C(*C*H_3)$_2$), 23.59 (s, C(*C*H_3)$_2$), 23.43 (s, CH(*C*H_3)$_2$), 22.30 (s, CH(*C*H_3)$_2$), 11.67 (s, *C*H_3C=C*C*H_3).

^{11}B{^{1}H} NMR (160 MHz, Pyridine-d_5) δ/ppm -9.82.

HR-MS (ESI): (m/z) calcd for $[M+H]^+$ ($C_{37}H_{53}B_2N_4O_1^+$) 592.4434; Found: 592.4474.

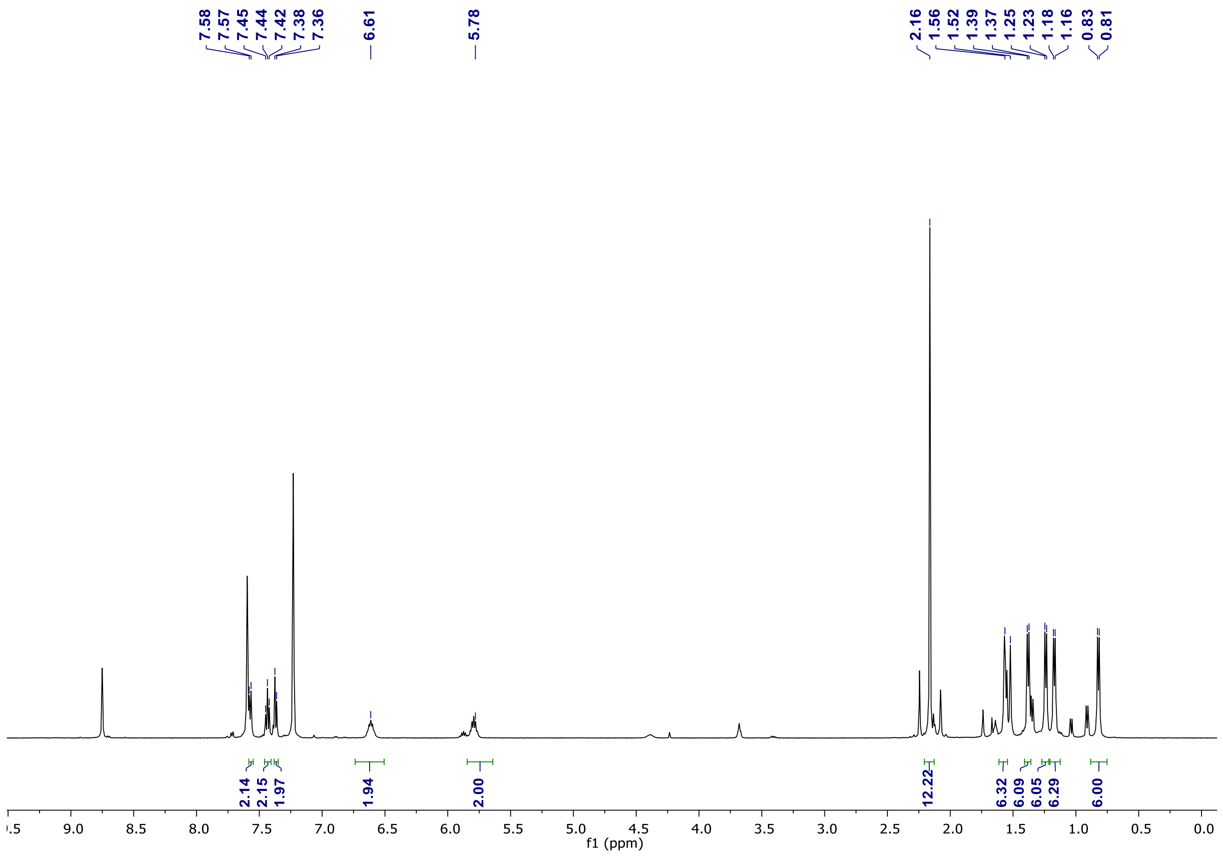

Figure S14. ^{1}H NMR spectrum of **4** in pyridine-d_5.

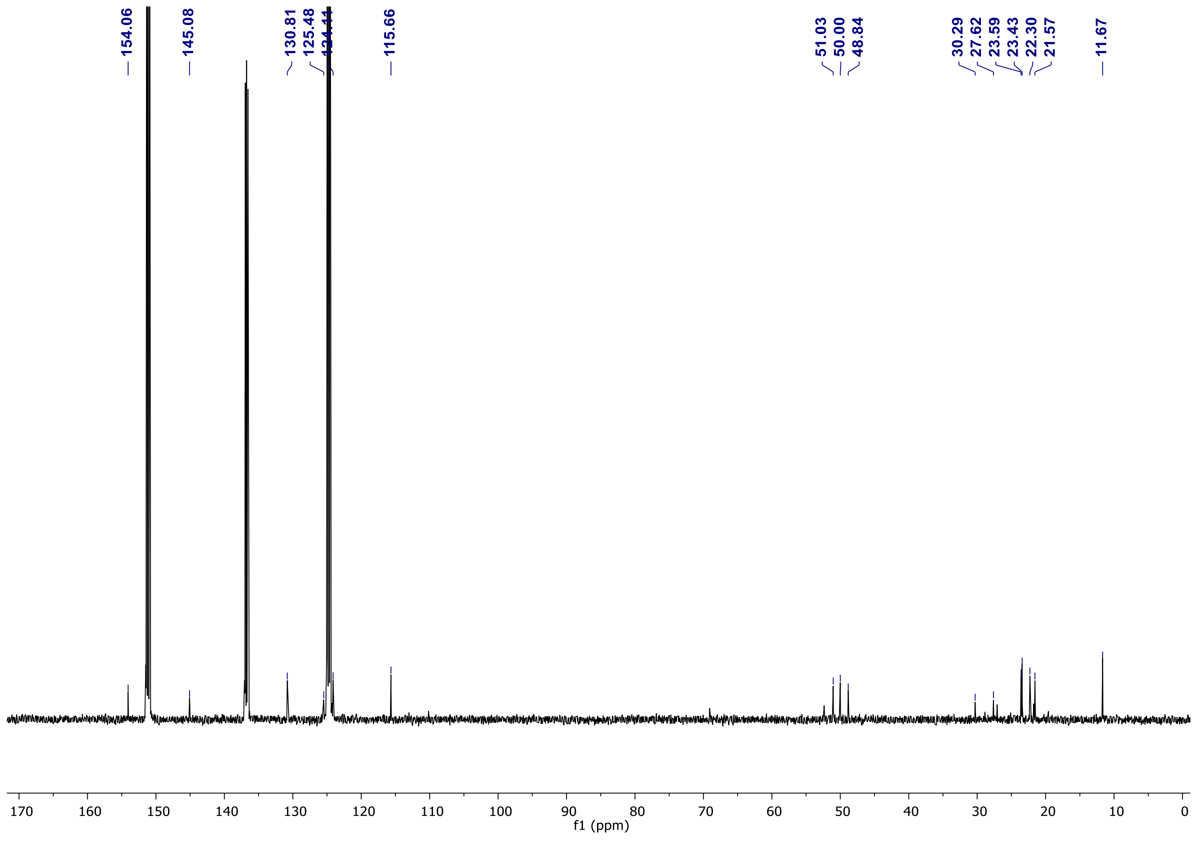

Figure S15. $^{13}C\{^{1}H\}$ NMR spectrum of **4** in pyridine-d_5.

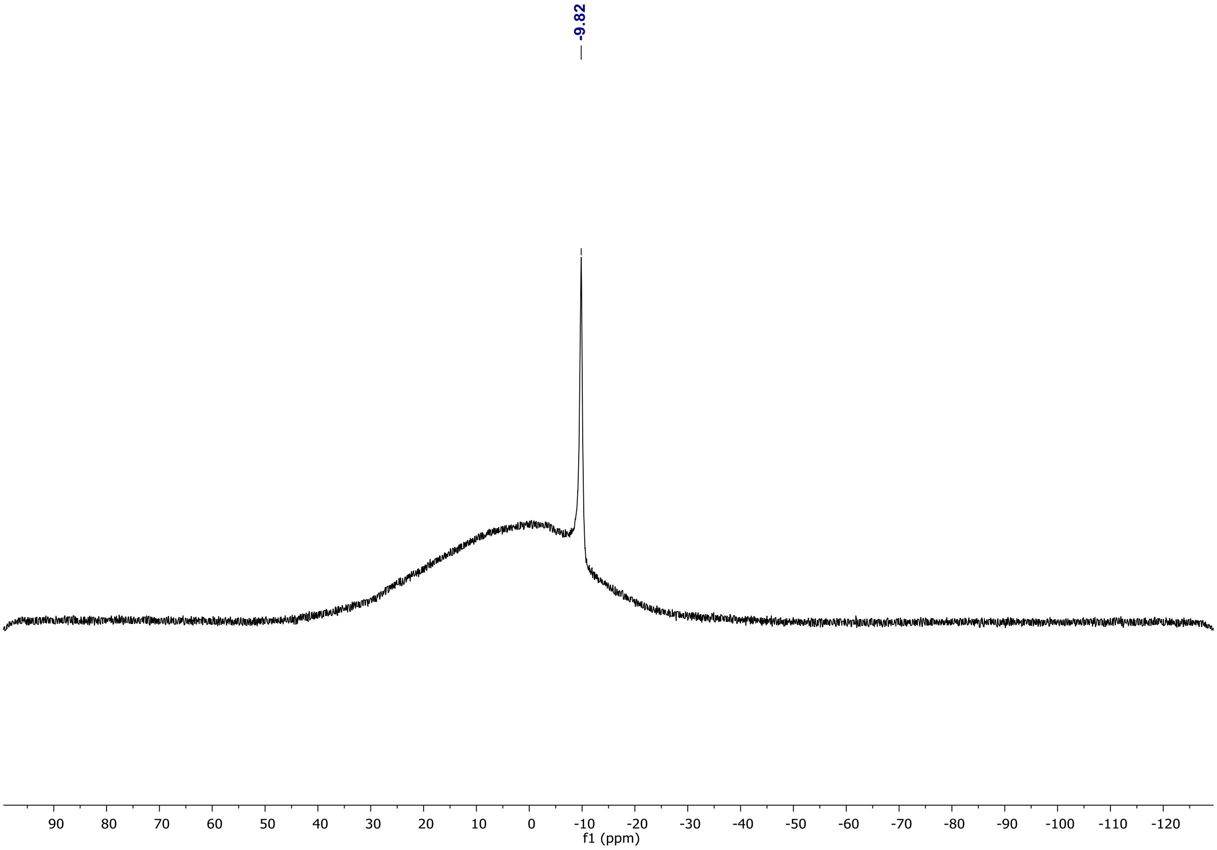

Figure S16. $^{11}B\{^{1}H\}$ NMR spectrum of **4** in pyridine-d_5.

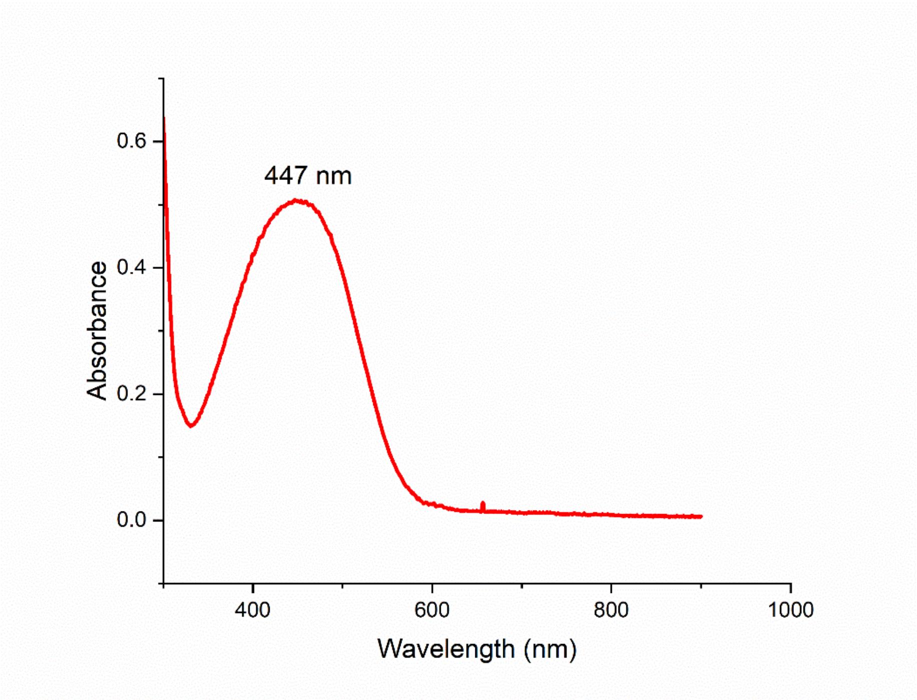

Figure S17. UV-vis absorption spectrum of **4** in THF.

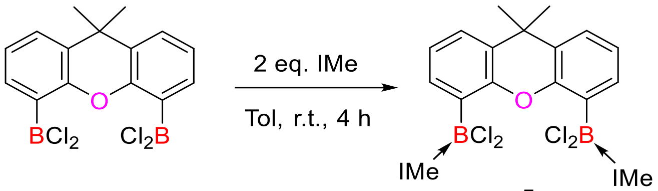

Synthesis of compound 5. Toluene (60 mL) was added into a 100 mL Schlenk flask containing **BCl_2(Xant)BCl_2** (372 mg, 1 mmol) and IMe (248 mg, 2 mmol) at room temperature. The reaction mixture was stirred at room temperature for 4 hours. The resulting solution was filtered. The filtrate was concentrated to afford compound **5** as colorless crystals in 85% yield (527 mg).

M.p.: 207.4 °C.

^{1}H NMR (500 MHz, THF-d_8) δ/ppm 8.02 (d, J = 7.5, Hz, 2H, Ar-H), 7.38 (d, J = 7.7, Hz, 2H, Ar-H), 6.95 (t, J = 7.6 Hz, 2H, Ar-H), 3.52 (br, 12H, NCH_3), 2.10 (s, 12H, CCH_3), 1.61 (s, 6H, C(CH_3)$_2$).

^{13}C{^{1}H} NMR (126 MHz, THF-d_8) δ/ppm 154.01, 134.36, 129.03, 128.30, 126.57, 126.09 (s, Ar-C), 122.49 (s, CH_3C=CCH_3), 34.51 (s, C(CH_3)$_2$), 34.31(s, NCH_3), 33.50 (s, C(CH_3)$_2$), 8.54 (s, CCH_3).

^{11}B{^{1}H} NMR (160 MHz, THF-d_8) δ/ppm 1.53.

HR-MS (ESI): (m/z) calcd for $[M\text{-}Cl]^+$ ($C_{29}H_{36}B_2N_4O_1Cl_3^+$) 583.2135; Found: 583.2585.

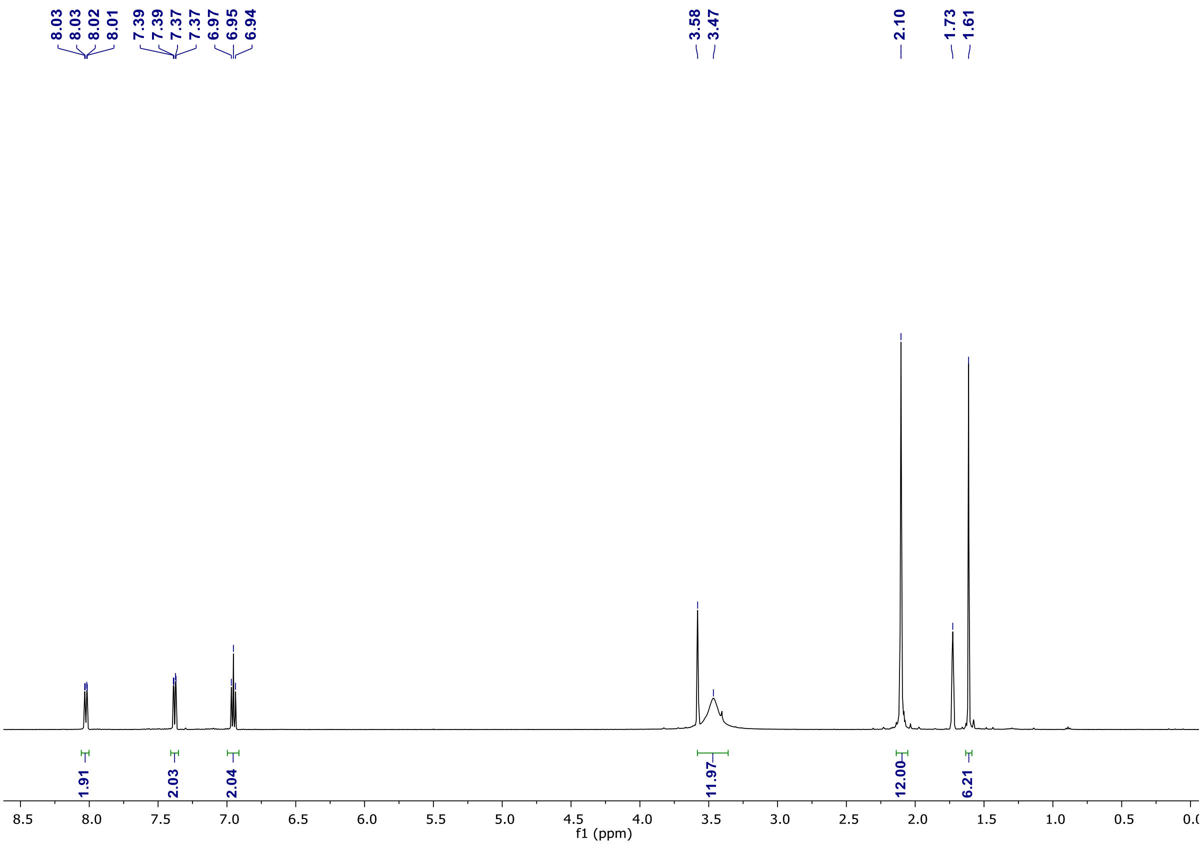

Figure S18. ^{1}H NMR spectrum of **5** in THF-d_8.

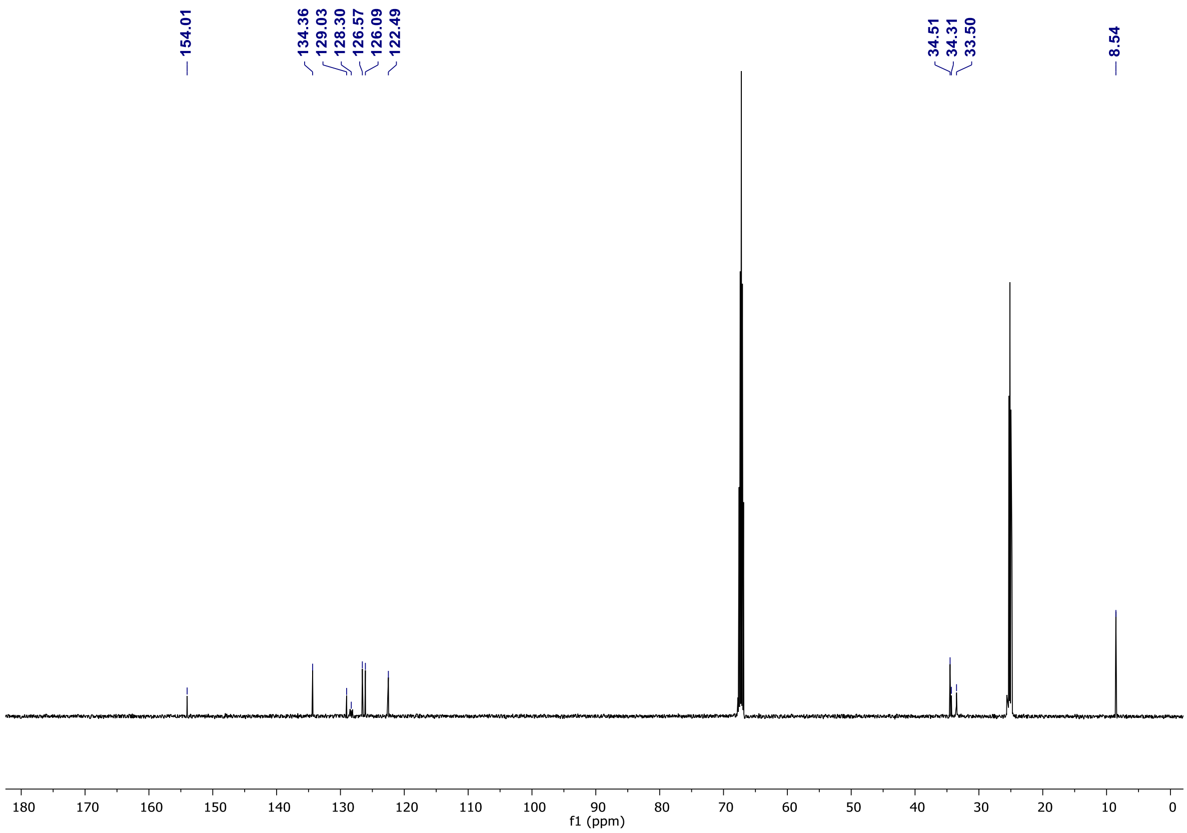

Figure S19. ^{13}C{^{1}H} NMR spectrum of **5** in THF-d_8.

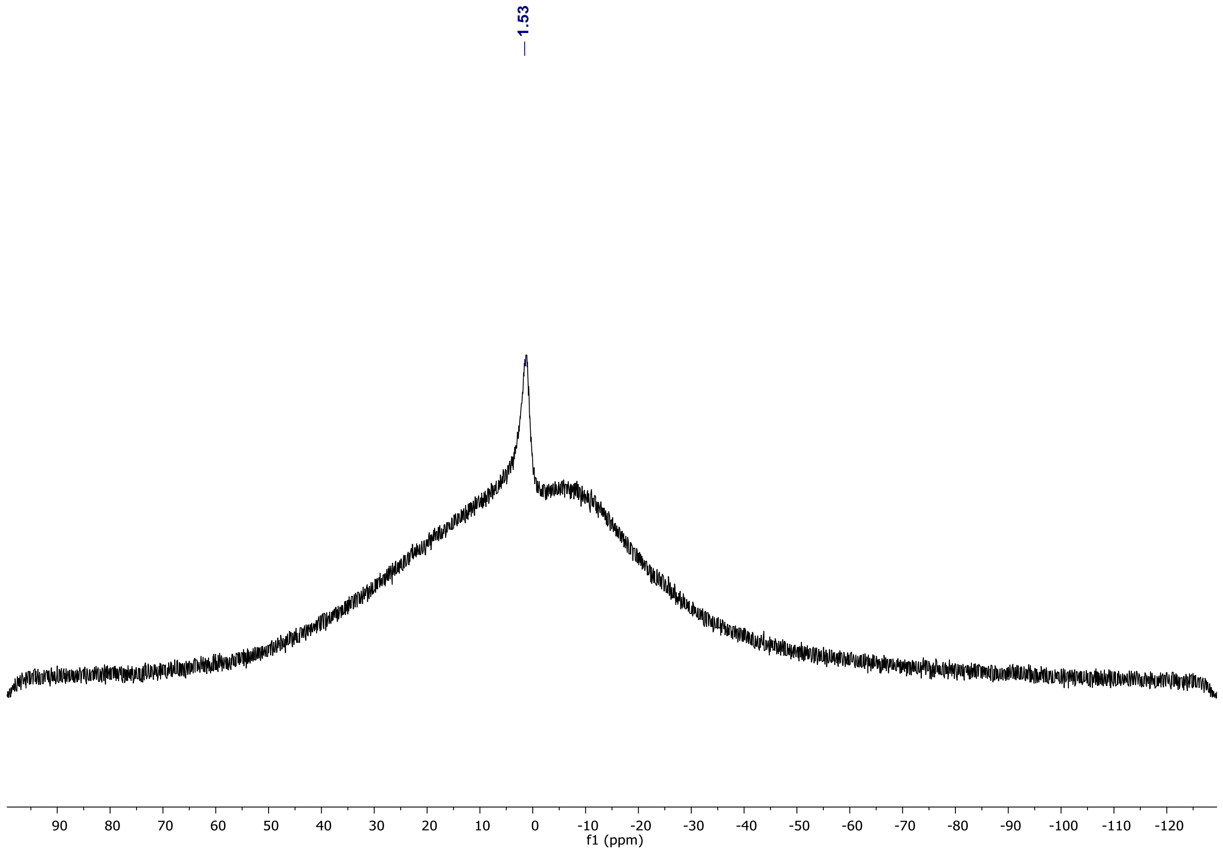

Figure S20. $^{11}B\{^{1}H\}$ NMR spectrum of **5** in THF-d_8.

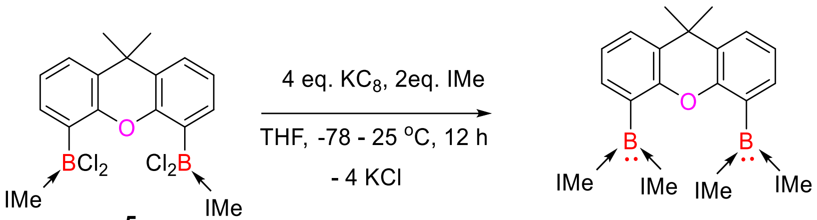

5 **6**

Synthesis of compound 6. THF (50 mL) was added into a 100 mL Schlenk flask containing **5** (620 mg, 1 mmol), IMe (248 mg, 2 mmol) and KC_8 (540 mg, 4 mmol) at room temperature. After 3 min, the color of the reaction mixture turned red. The resulting suspension was stirred at room temperature for overnight. The reaction mixture was filtered and the filtrare was concentrated and kept for one week at 4 °C to afford **6** as red crystals in 42% yield (305 mg).

M.p.: 182.6 °C.

^{1}H NMR (200 MHz, Benzene-d_6) δ/ppm 7.51 (d, J = 6.9 Hz, 2H, Ar-*H*), 7.20 (m, 2H, Ar-*H*), 7.10 (m, 2H, Ar-*H*), 2.82 (s, 24H, NC*H*$_3$), 1.94 (s, 6H, C(C*H*$_3$)$_2$), 1.67 (s, 24H, CC*H*$_3$).

^{13}C{^{1}H} NMR (101 MHz, Benzene-d_6) δ/ppm 154.65, 137.22, 129.61, 120.54 (s, Ar-*C*), 119.02 (s, CH$_3$*C*=*C*CH$_3$), 35.21 (s, *C*(CH$_3$)$_2$), 34.40 (s, N*C*H$_3$), 32.84 (s, C(*C*H$_3$)$_2$), 9.85 (s, *C*H$_3$C=C*C*H$_3$).

^{11}B{^{1}H} NMR (160 MHz, Benzene-d_6) δ/ppm -4.09.

HR-MS (ESI): (m/z) calcd for $[M+H]^+$ ($C_{43}H_{61}B_2N_8O_1^+$) 727.5149; Found: 727.5146.

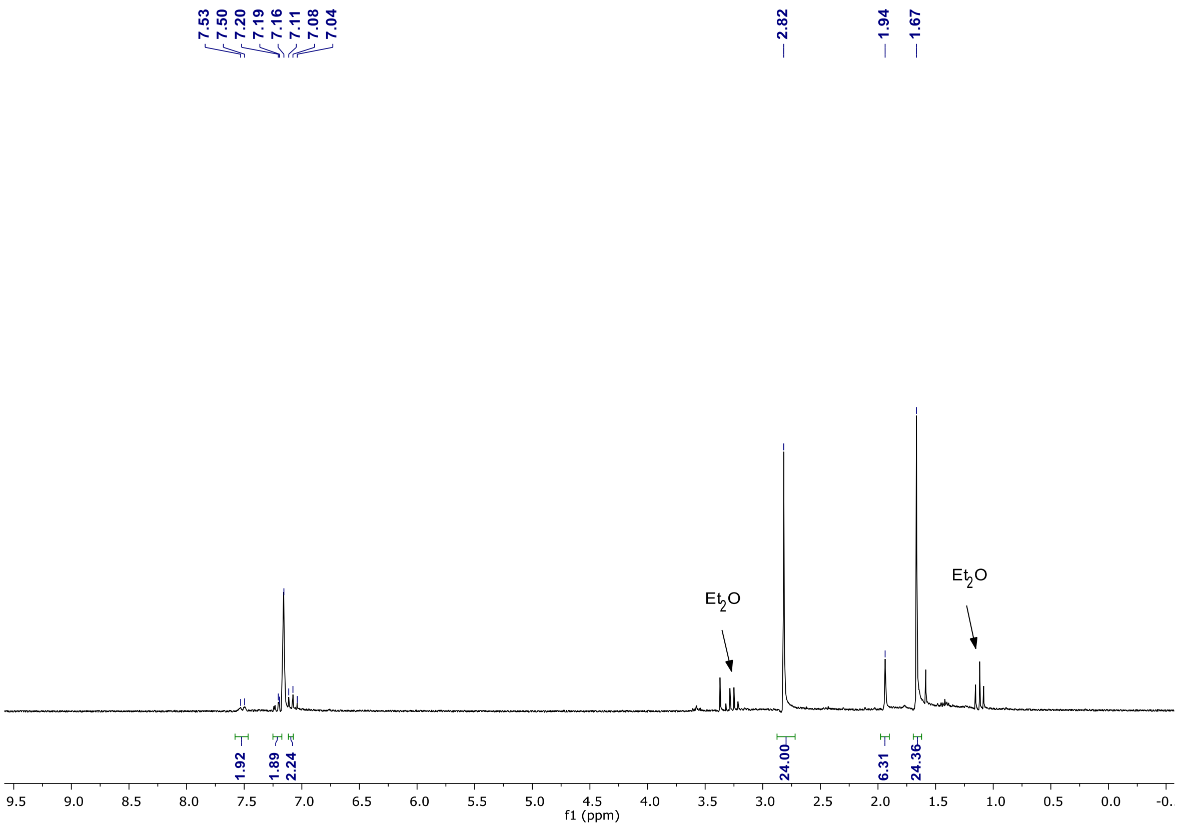

Figure S21. ^{1}H NMR spectrum of **6** in benzene-d_6.

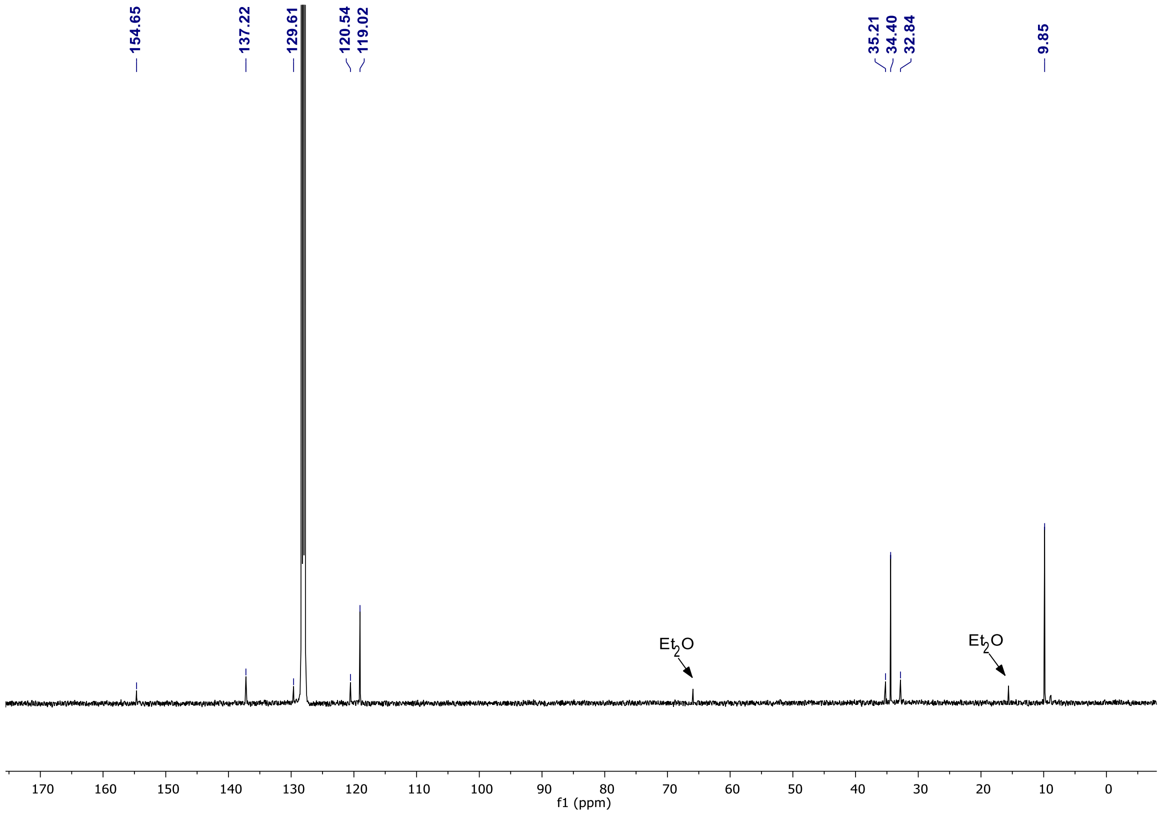

Figure S22. ^{13}C{^{1}H} NMR spectrum of **6** in benzene-d_6.

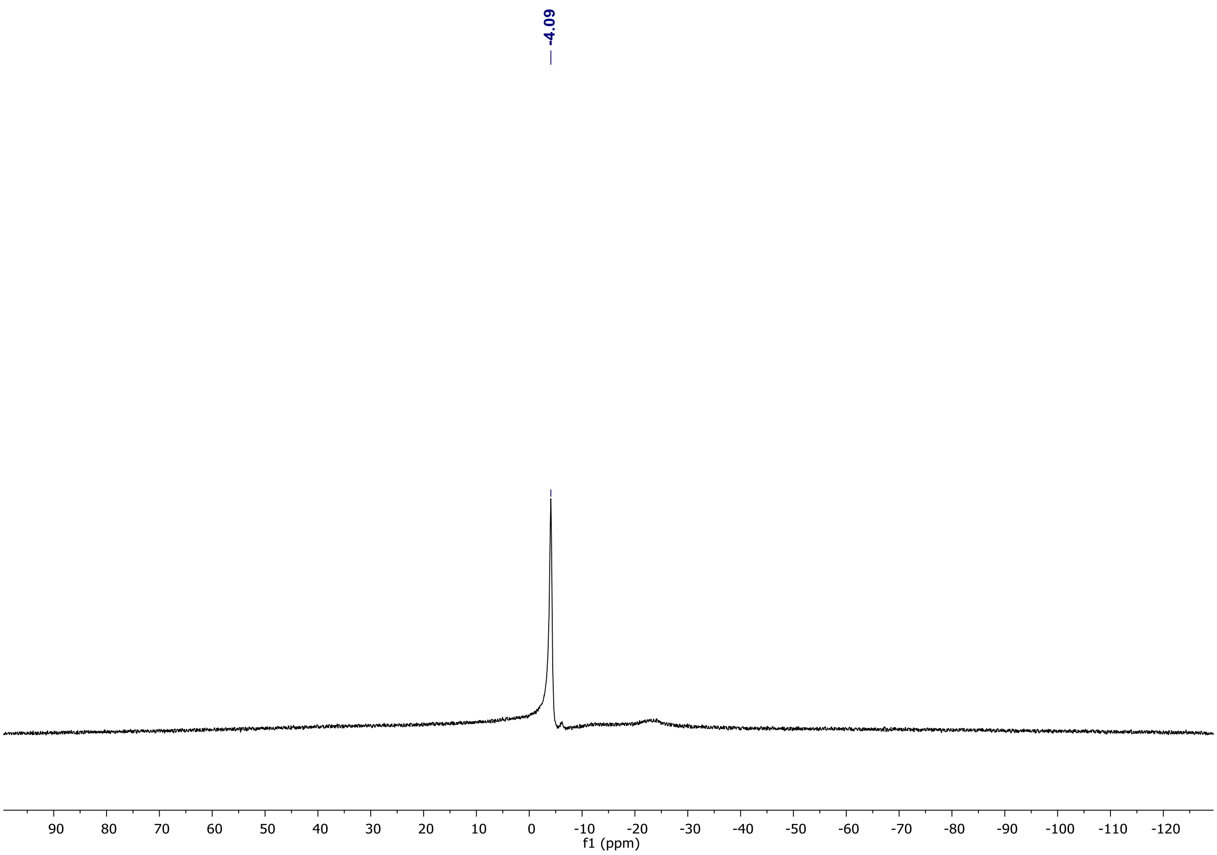

Figure S23. $^{11}B\{^{1}H\}$ NMR spectrum of **6** in benzene-*d*$_6$.

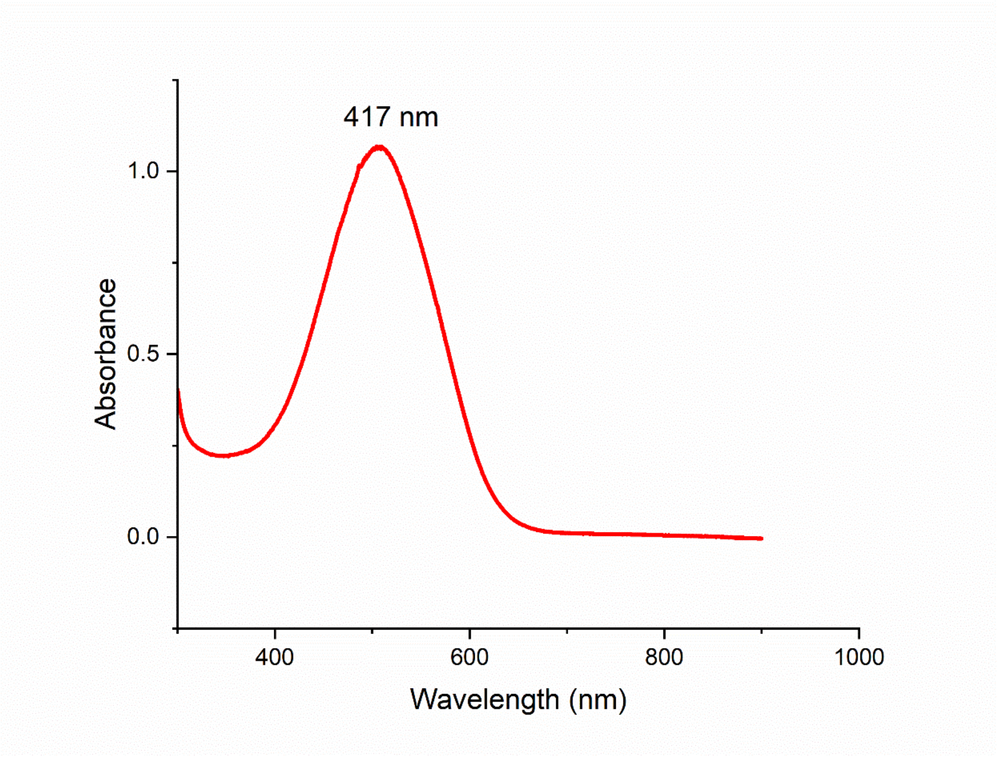

Figure S24. UV-vis absorption spectrum of **6** in THF.

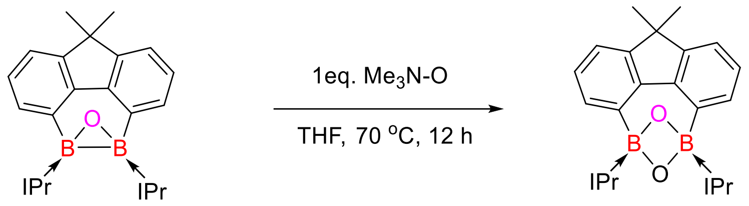

4 **7**

Synthesis of compound 7. THF (50 mL) was added into a 100 mL Schlenk flask containing **4** (177 mg, 0.3 mmol) and Me_3N-O (30 mg, 0.4 mmol) at room temperature. The reaction mixture was stirred at 70 °C for 12 hours. The resulting yellow solution was filtered. The filtrate was concentrated to afford compound **7** as colorless crystals in 83% yield (147 mg).

M.p.: 235.3 °C.

^{1}H NMR (500 MHz, Pyridine-d_5) δ/ppm 7.38 (d, J = 7.4 Hz, 2H, Ar-H), 7.18 (m, 2H, Ar-H), 6.70 (d, J = 6.9 Hz, 2H, Ar-H), 6.62 – 6.48 (sept, J = 7.2 Hz, 4H, CH(CH_3)$_2$), 2.20 (s, 12H, CCH_3), 1.51 (s, 6H, C(CH_3)$_2$), 1.44 (d, J = 7.0 Hz, 12H, CH(CH_3)$_2$), 1.26 (d, J = 7.2 Hz, 12H, CH(CH_3)$_2$).

^{13}C{^{1}H} NMR (126 MHz, Pyridine-d_5) δ/ppm 152.64, 147.78, 129.87, 129.13, 126.23 (s, Ar-C), 119.41 (s, $CH_3$$C$=$C$$CH_3$), 49.21 (s, C(CH_3)$_2$), 47.21 (s, C(CH$_3$)$_2$), 28.14 (s, CH(CH_3)$_2$), 22.44 (s, CH(CH$_3$)$_2$), 22.08 (s, CH(CH$_3$)$_2$), 10.71 (s, CH$_3$C=CCH$_3$).

^{11}B{^{1}H} NMR (160 MHz, Pyridine-d_5) δ/ppm 8.55.

HR-MS (ESI): (m/z) calcd for $[M+H]^+$ ($C_{37}H_{53}B_2N_4O_2^+$) 607.4349; Found: 607.4351.

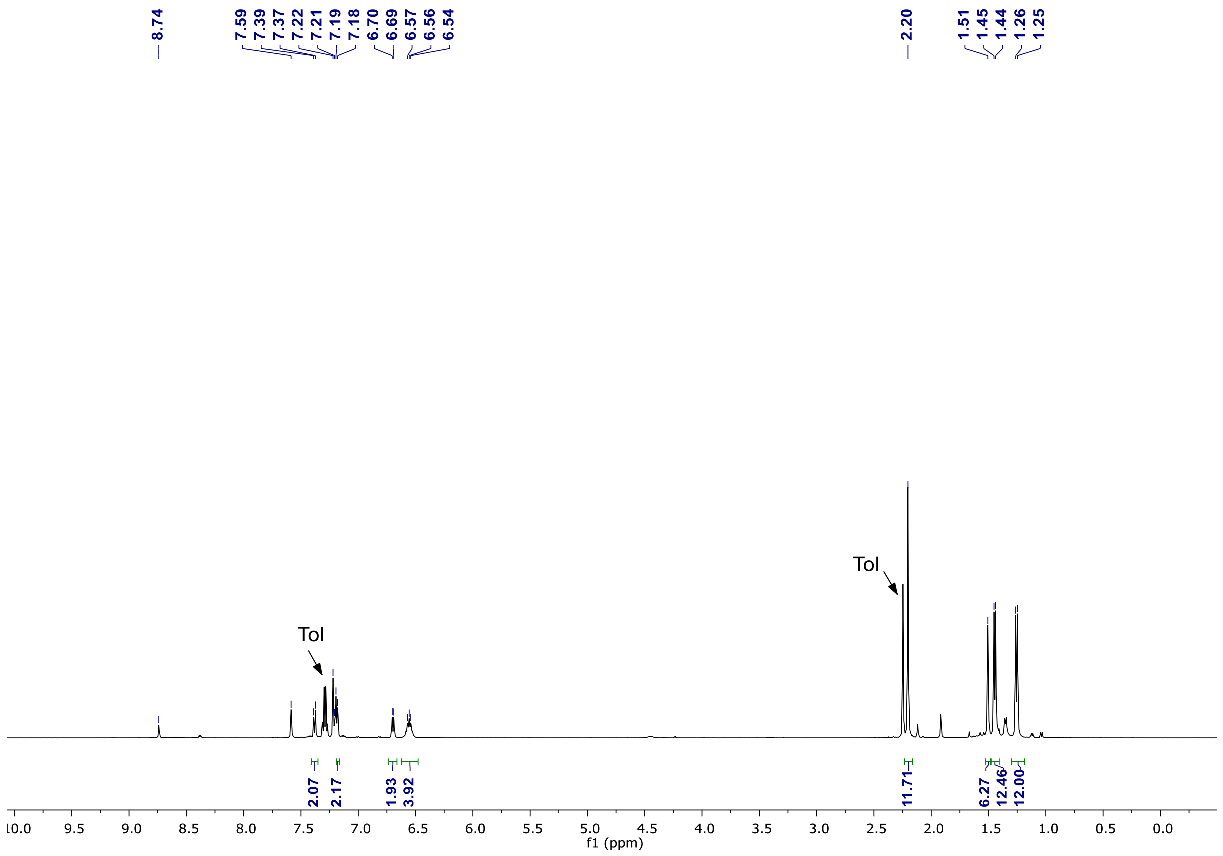

Figure S25. ^{1}H NMR spectrum of **7** in pyridine-d_5.

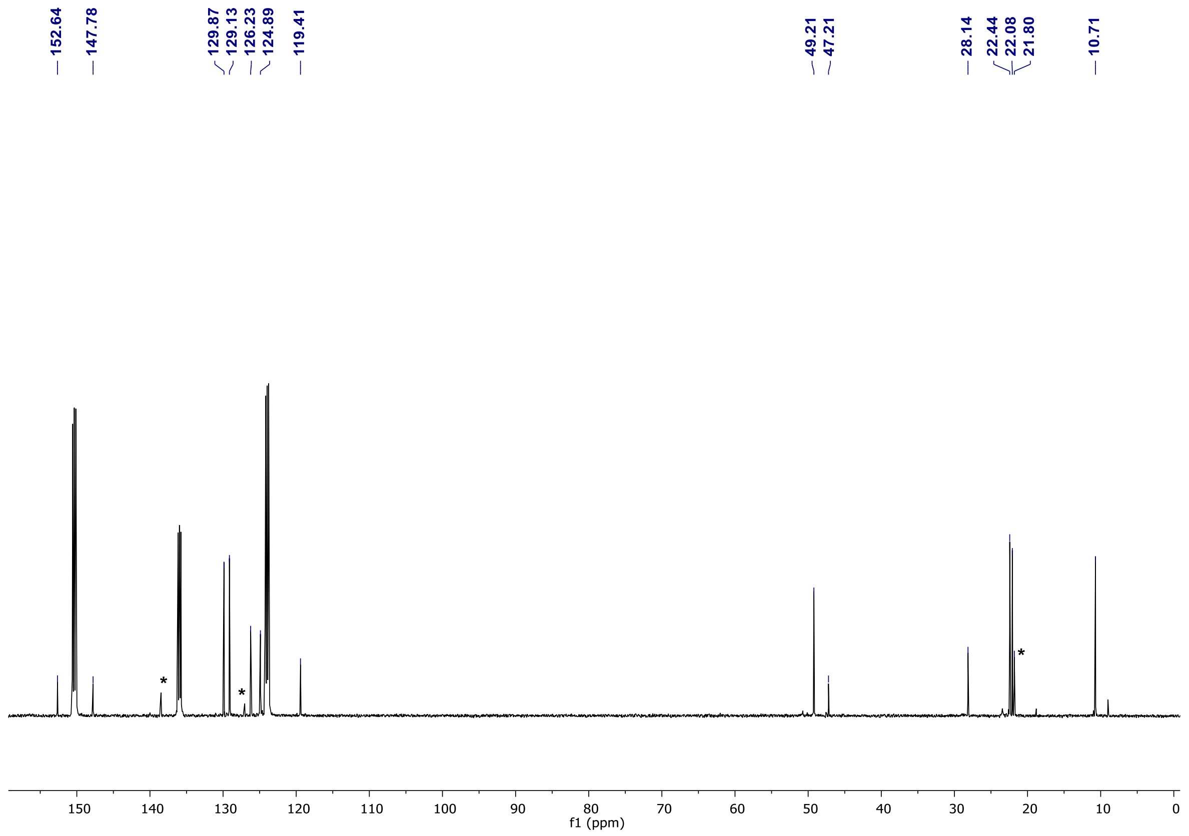

Figure S26. $^{13}C\{^{1}H\}$ NMR spectrum of **7** in pyridine-d_5. * is toluene.

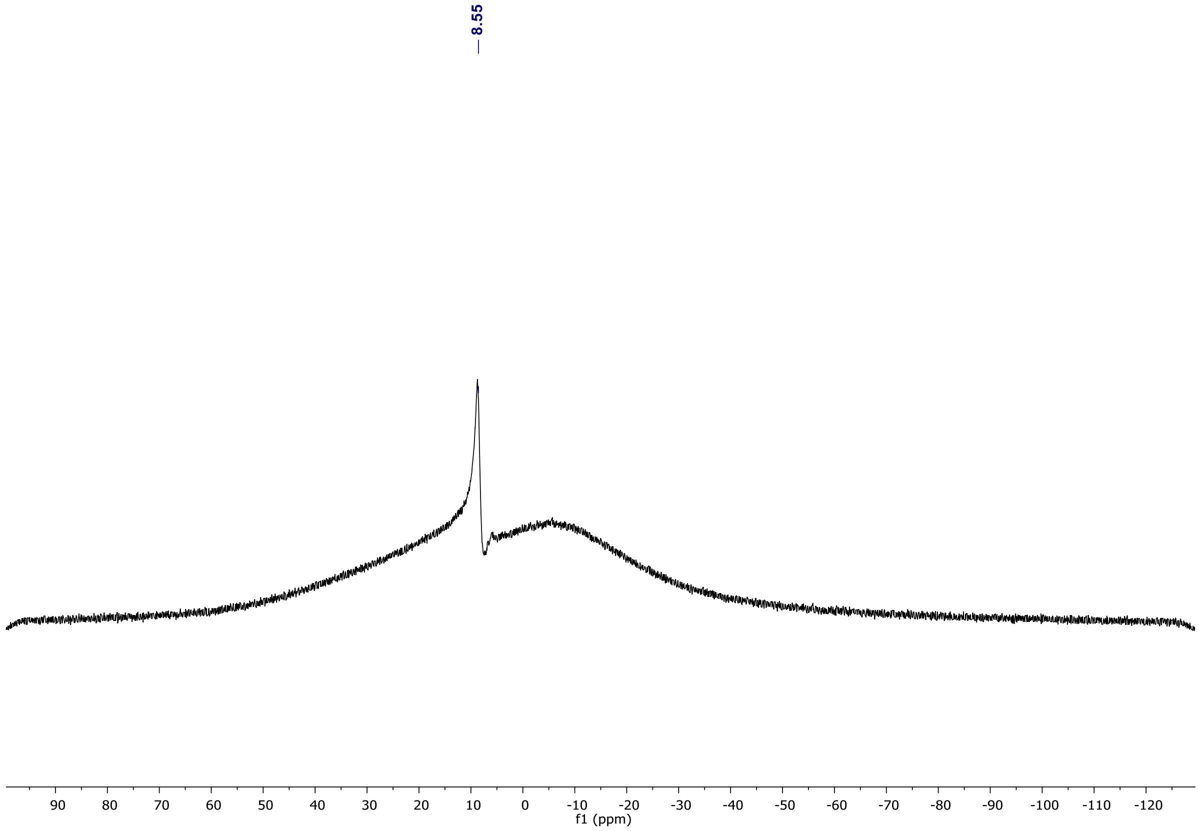

Figure S27. $^{11}B\{^{1}H\}$ NMR spectrum of **7** in pyridine-d_5.

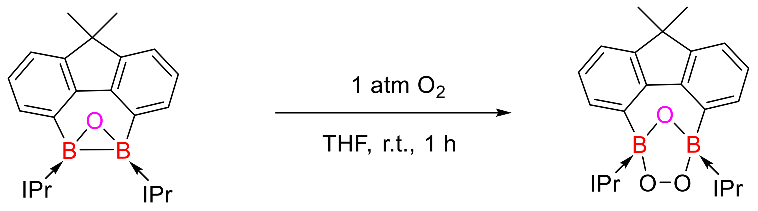

4 8

Synthesis of compound 8. After three freeze-pump thaw cycles, the THF solution of compound **4** (177 mg, 0.3 mmol) in a 100 mL Schlenk flask was subjected to 1 atm O_2 at room temperature under stirring. After 1 hour, the color of the mixture changed from orange-red to colorless. The resulting colorless solution was filtered. The filtrate was concentrated to afford compound **8** as colorless crystals in 90% yield (170 mg).

M.p.: 182.6 °C.

^{1}H NMR (400 MHz, Methylene Chloride-d_2) δ/ppm 7.14 (d, J = 7.1 Hz, 2H, Ar-H), 6.90 (t, J = 7.3 Hz, 2H, Ar-H), 6.36 (d, J = 7.2 Hz, 2H, Ar-H), 6.15 (sept, J = 7.0 Hz, 2H, CH(CH$_3$)$_2$), 5.81 (br, 2H, CH(CH$_3$)$_2$), 2.31 (s, 6H, C=CCH_3), 2.26 (s, 6H, C=CCH_3), 1.54 (d, J = 6.9 Hz, 6H, CH(CH_3)$_2$), 1.50 (d, J = 7.2 Hz, 6H, CH(CH_3)$_2$), 1.44 – 1.42 (12H, CH(CH_3)$_2$ and C(CH_3)$_2$; overlapping), 1.05 (d, J = 7.1 Hz, 6H, CH(CH_3)$_2$).

^{13}C{^{1}H} NMR (101 MHz, Methylene Chloride-d_2) δ/ppm 152.70, 146.37, 130.37, 125.22, 124.19 (s, Ar-C), 119.17 (s, CH$_3$$C$=$CCH_3$), 51.49 (s, CH(CH$_3$)$_2$), 50.61 (s, CH(CH$_3$)$_2$), 49.72 (s, C(CH$_3$)$_2$), 28.29 (s, C(CH$_3$)$_2$), 28.08 (s, C(CH$_3$)$_2$), 22.43 (s, CH(CH$_3$)$_2$), 22.37 (s, CH(CH$_3$)$_2$), 22.27 (s, CH(CH$_3$)$_2$), 22.14 (s, CH(CH$_3$)$_2$), 11.01 (s, C=CCH$_3$), 8.86 (s, C=CCH$_3$).

^{11}B{^{1}H} NMR (128 MHz, Methylene Chloride-d_2) δ/ppm 5.12.

HR-MS (ESI): (m/z) calcd for $[M+H]^+$ ($C_{37}H_{53}B_2N_4O_3^+$) 623.4298; Found: 623.4322.

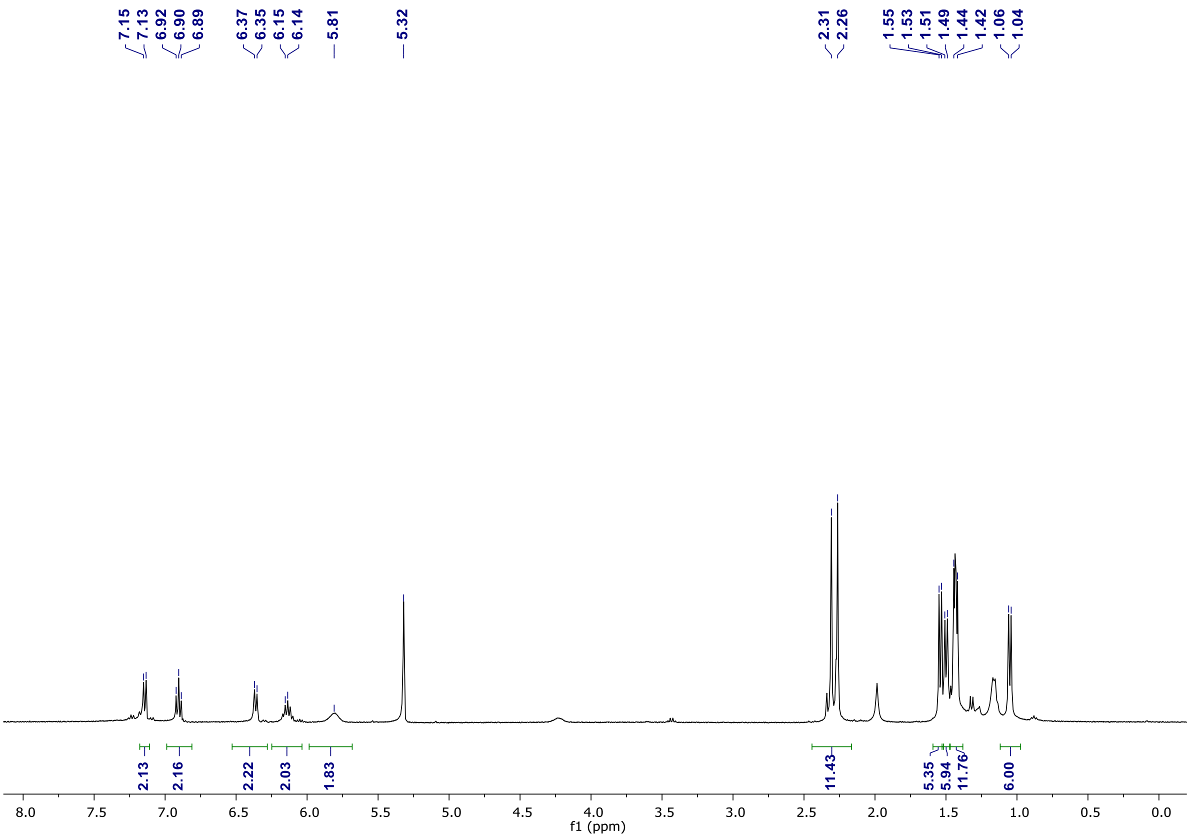

Figure S28. ^{1}H NMR spectrum of **8** in methylene chloride-d_2. * is toluene.

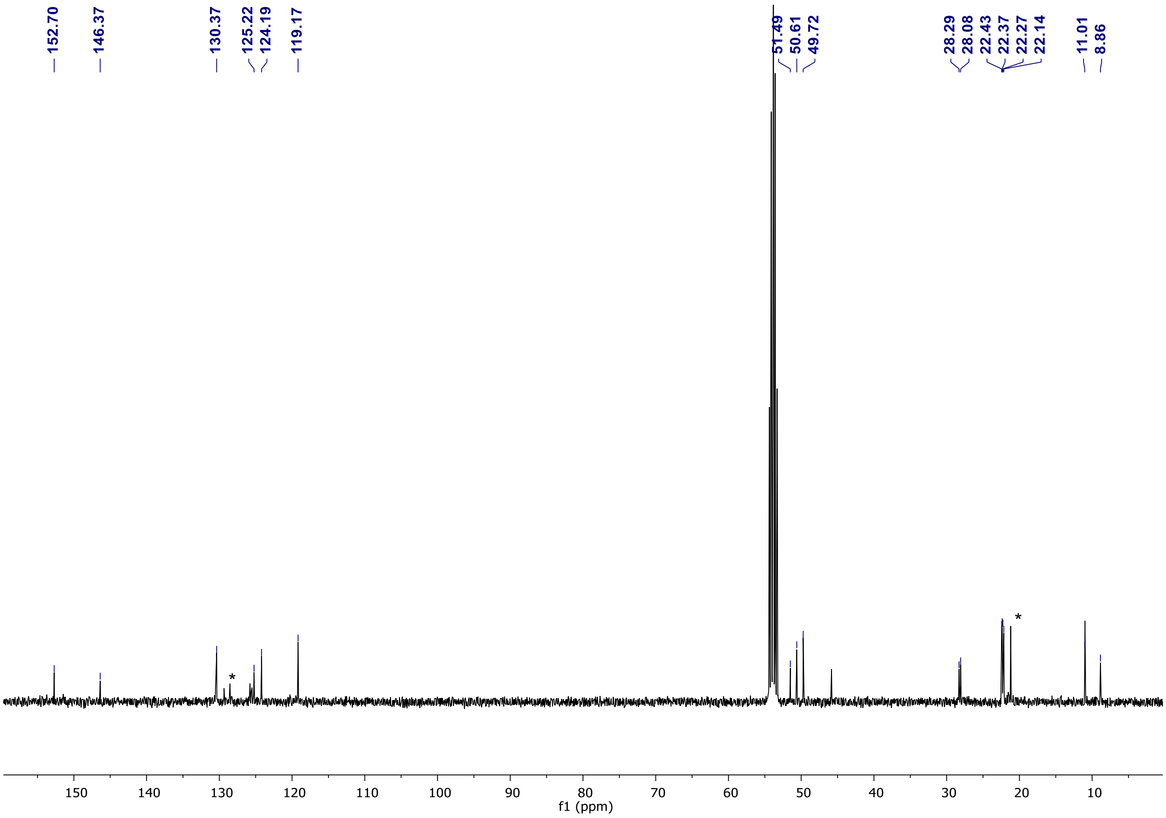

Figure S29. $^{13}C\{^{1}H\}$ NMR spectrum of **8** in methylene chloride-d_2. * is toluene.

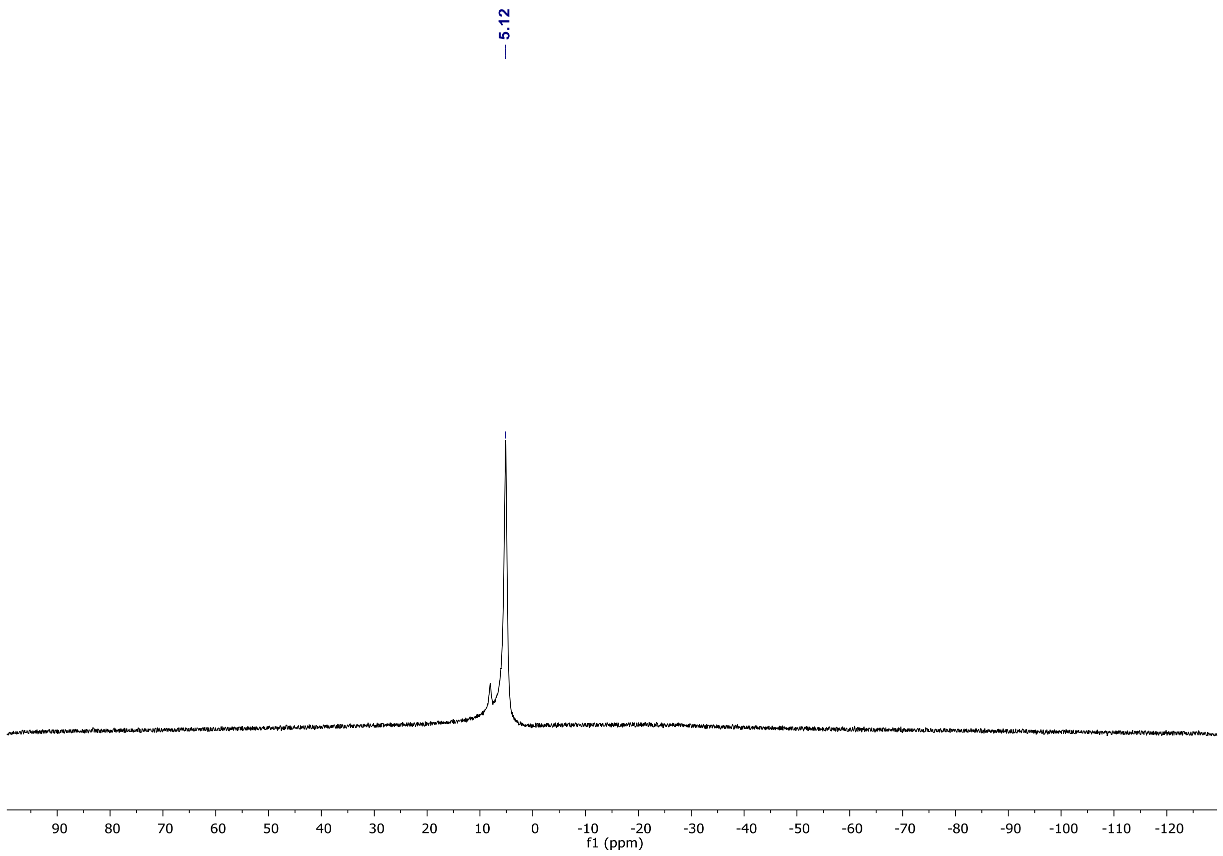

Figure S30. $^{11}B\{^{1}H\}$ NMR spectrum of **8** in methylene chloride-d_2.

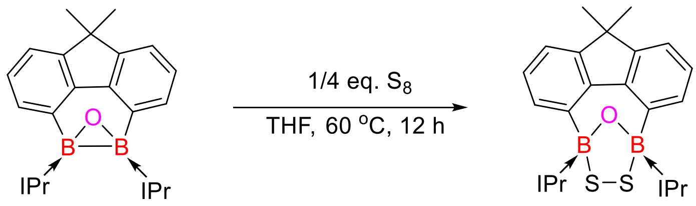

4 9

Synthesis of compound 9. THF (50 mL) was added into a 100 mL Schlenk flask containing **4** (177 mg, 0.3 mmol) and sulfur powder (20 mg, 0.6 mmol) at room temperature. The reaction mixture was stirred at 60 °C for 12 hours. The color of the reaction solution changed from orange-red to yellow. The resulting yellow solution was filtered. The filtrate was concentrated to afford compound **9** as colorless crystals in 62% yield (122 mg).

M.p.: 160.9 °C.

^{1}H NMR (500 MHz, Pyridine-d_5) δ/ppm 7.44 (d, J = 7.4 Hz, 2H, Ar-H), 7.30 (t, J = 7.2 Hz, 2H, Ar-H), 6.71 (d, J = 7.1 Hz, 2H, Ar-H), 6.48 (sept, J = 6.8 Hz, 2H, CH(CH$_3$)$_2$), 6.11 (sept, J = 7.0 Hz, 2H, CH(CH$_3$)$_2$), 2.18 (s, 6H, C=CCH_3), 2.13 (s, 6H, C=CCH_3), 1.66 (d, J = 6.9 Hz, 6H, CH(CH_3)$_2$), 1.57 (s, 3H, C(CH_3)$_2$), 1.51 (s, 3H, C(CH_3)$_2$), 1.35 (d, J = 7.0 Hz, 6H, CH(CH_3)$_2$), 1.21 (d, J = 7.1 Hz, 6H, CH(CH_3)$_2$), 1.08 (d, J = 7.0 Hz, 6H, CH(CH_3)$_2$).

^{13}C{^{1}H} NMR (126 MHz, Pyridine-d_5) δ/ppm 154.52, 129.45, 126.38, 126.20, 125.76 (s, Ar-C), 120.46 (s, CH$_3$$C$=$CCH_3$), 52.20 (s, CH(CH$_3$)$_2$), 50.58 (s, CH(CH$_3$)$_2$), 49.86 (s, C(CH$_3$)$_2$), 28.33 (s, C(CH$_3$)$_2$), 27.07 (s, C(CH$_3$)$_2$), 23.38 (s, CH(CH$_3$)$_2$), 23.23 (s, CH(CH$_3$)$_2$), 23.12 (s, CH(CH$_3$)$_2$), 22.59 (s, CH(CH$_3$)$_2$), 11.82 (s, C=CCH$_3$), 11.56 (s, C=CCH$_3$).

^{11}B{^{1}H} NMR (160 MHz, Pyridine-d_5) δ/ppm 4.87.

HR-MS (ESI): (m/z) calcd for $[M+H]^+$ ($C_{37}H_{53}B_2N_4O_1S_2^+$) 655.3841; Found: 655.3849.

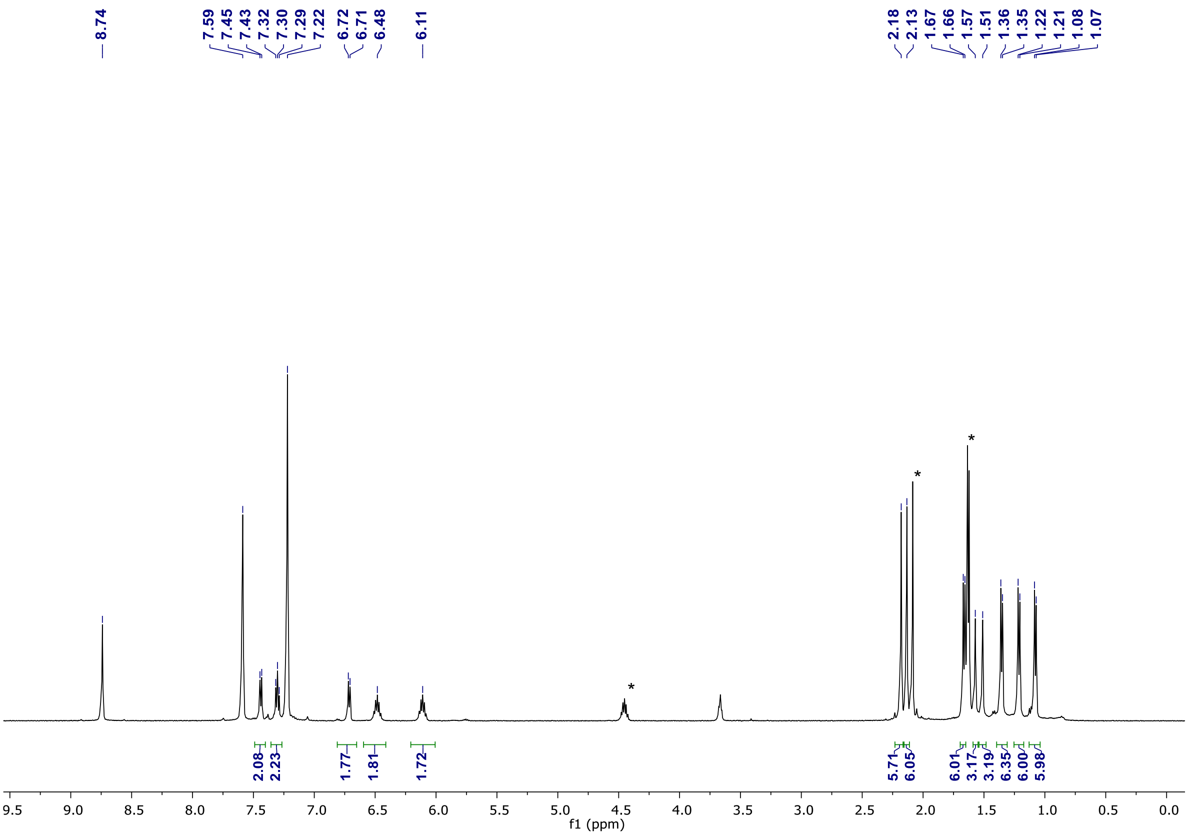

Figure S31. ^{1}H NMR spectrum of **9** in pyridine-d_5. * is ^{iPr}NHC=S.

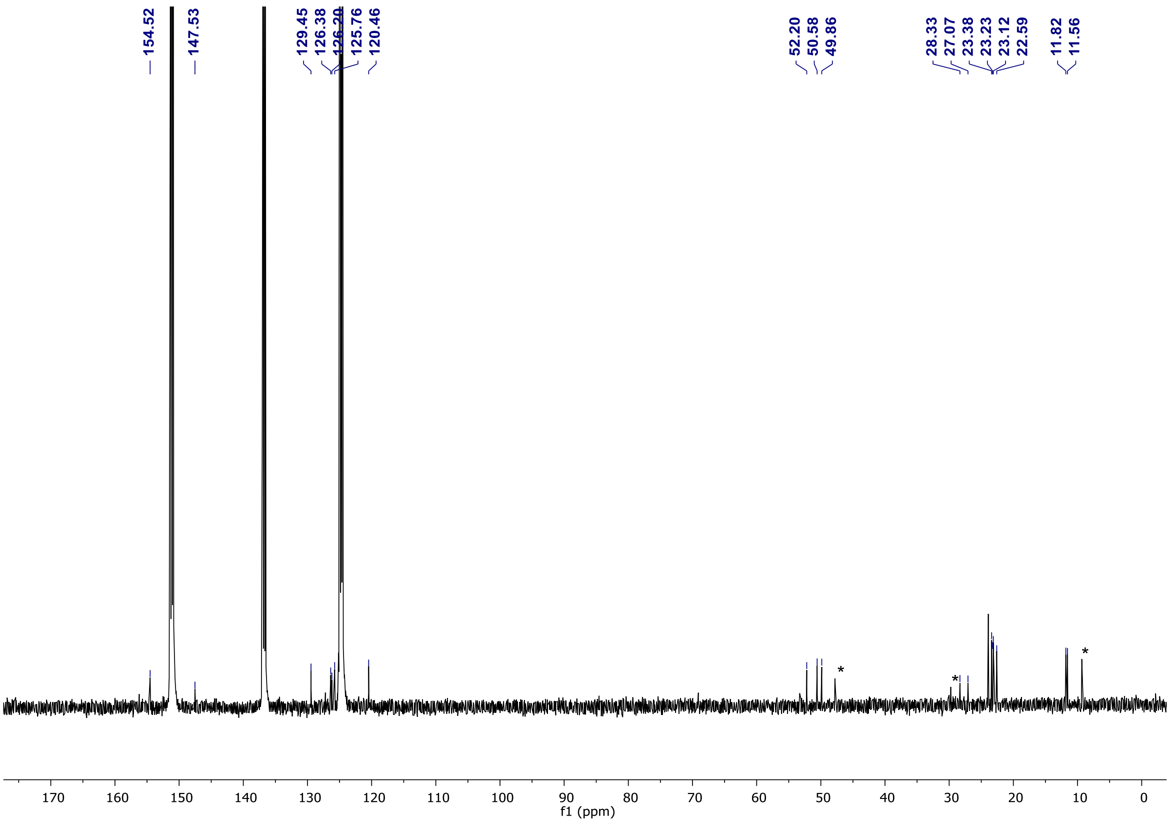

Figure S32. $^{13}C\{^{1}H\}$ NMR spectrum of **9** in pyridine-d_5. * is ^{iPr}NHC=S.

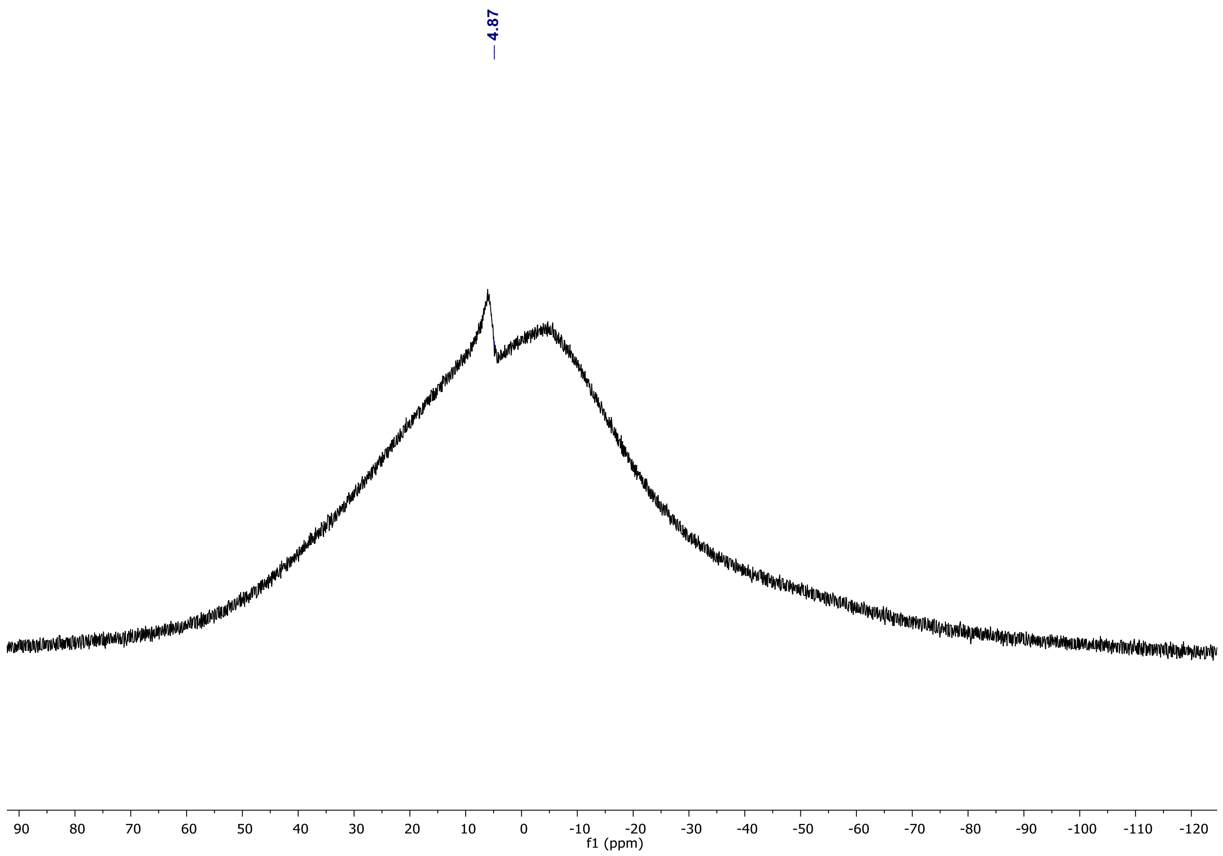

Figure S33. $^{11}B\{^{1}H\}$ NMR spectrum of **9** in pyridine-d_5.

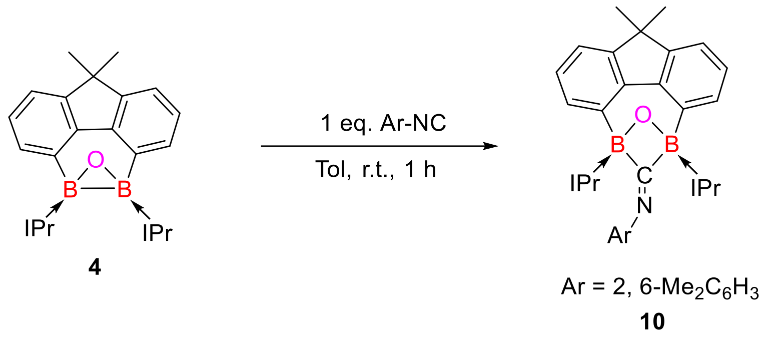

Synthesis of compound 10. Toluene (50 mL) was added into a 100 mL Schlenk flask containing **4** (177 mg, 0.3 mmol) and 2, 6-dimethylphenyl isocyanide (40 mg, 0.3 mmol) at room temperature. The reaction mixture was stirred at room temperature for 1 hour. The color of the reaction solution changed from orange-red to green. The resulting green solution was filtered. The filtrate was dried under vacuum to remove all volatiles and afford a green solid, which was washed with hexane (10 mL) to give product **10** as a yellow solid in 88% yield (190 mg). Colorless crystals suitable for X-ray diffraction analysis were obtained from a DME solution at 4 ºC.

M.p.: 242.6 ºC.

^{1}H NMR (400 MHz, THF-*d*$_8$) *δ*/ppm 7.16 (br, 2H, C*H*(CH$_3$)$_2$), 6.96 – 6.87 (m, 2H, Ar-*H*), 6.68 (m, 3H, Ar-*H*), 6.53 (d, *J* = 7.4 Hz, 1H, Ar-*H*), 6.37 (m, 1H, Ar-*H*), 5.89 (d, *J* = 7.2 Hz, 2H, Ar-*H*), 5.34 (br, 2H, C*H*(CH$_3$)$_2$), 2.29 (s, 6H, C=CC*H*$_3$), 2.27 (s, 6H, C=CC*H*$_3$), 2.03 (s, 3H, Ar-C*H*$_3$), 1.45 (d, *J* = 6.9 Hz, 6H, CH(C*H*$_3$)$_2$), 1.40 (s, 6H, C(C*H*$_3$)$_2$), 1.38 – 1.27 (d, *J* = 7.0 Hz, 6H, CH(C*H*$_3$)$_2$), 1.10 (d, *J* = 7.1 Hz, 6H, CH(C*H*$_3$)$_2$), 0.98 (s, 3H, Ar-C*H*$_3$), 0.93 (d, *J* = 6.8 Hz, 6H, CH(C*H*$_3$)$_2$).

^{13}C{^{1}H} NMR (101 MHz, THF-*d*$_8$) *δ*/ppm 152.07, 147.29, 129.77, 129.06, 126.83, 124.63, 124.44, 124.20, 124.07 (s, Ar-*C*), 118.84 (s, CH$_3$*C*=*C*CH$_3$), 118.49 (s, CH$_3$*C*=*C*CH$_3$), 51.01 (s, *C*H(CH$_3$)$_2$), 48.86 (s, *C*H(CH$_3$)$_2$),, 45.93 (s, *C*(CH$_3$)$_2$), 28.86 (s, C(*C*H$_3$)$_2$), 27.34 (s, C(*C*H$_3$)$_2$), 22.97 (s, CH(*C*H$_3$)$_2$), 22.51 (s, CH(*C*H$_3$)$_2$), 21.40 (s, CH(*C*H$_3$)$_2$), 21.09 (s, *C*H$_3$-Ar), 20.89 (s, CH(*C*H$_3$)$_2$), 17.30 (s, *C*H$_3$-Ar), 10.78 (s, C=C*C*H$_3$), 10.68 (s, C=C*C*H$_3$).

^{11}B{^{1}H} NMR (128 MHz, THF-*d*$_8$) *δ*/ppm 3.64.

HR-MS (ESI): (m/z) calcd for $[M+H]^+$ ($C_{46}H_{62}B_2N_5O_1^+$) 722.5135; Found: 722.5066.

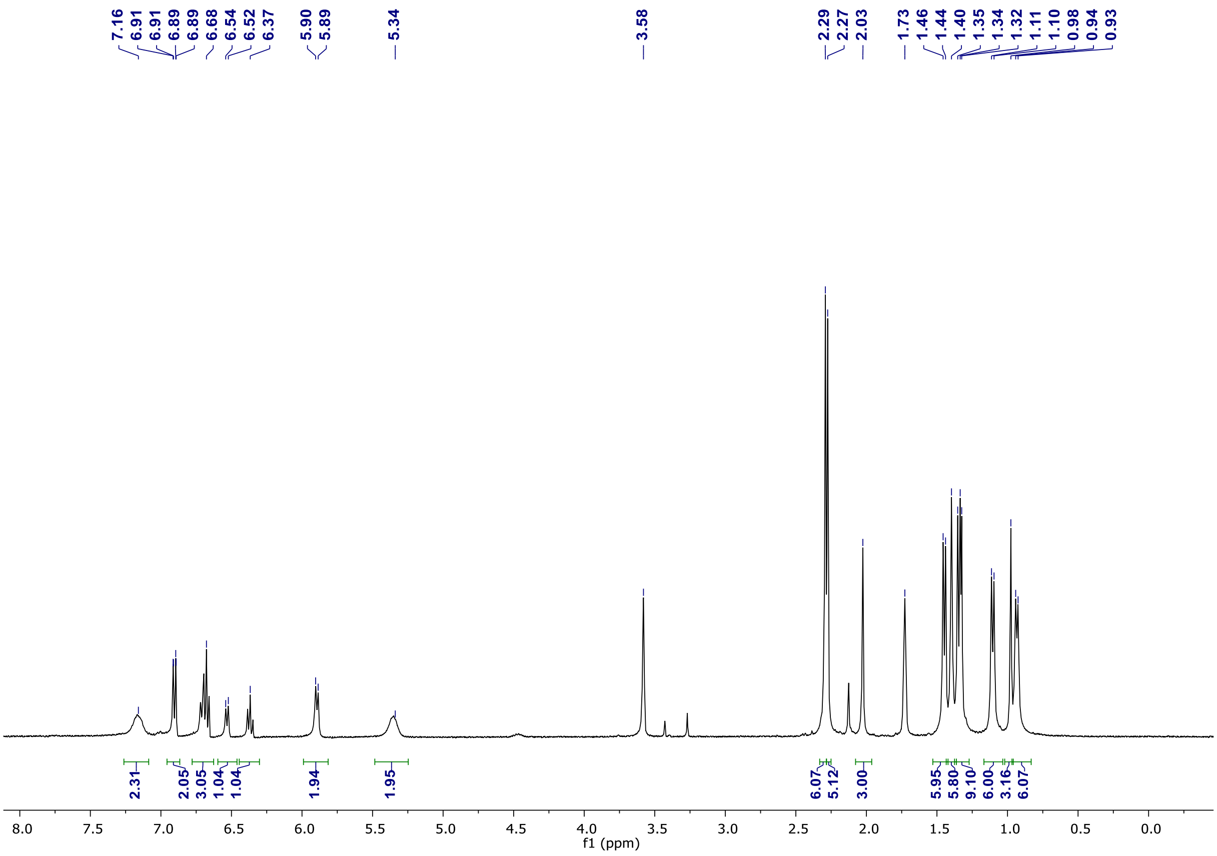

Figure S34. ^{1}H NMR spectrum of **10** in THF-d_8.

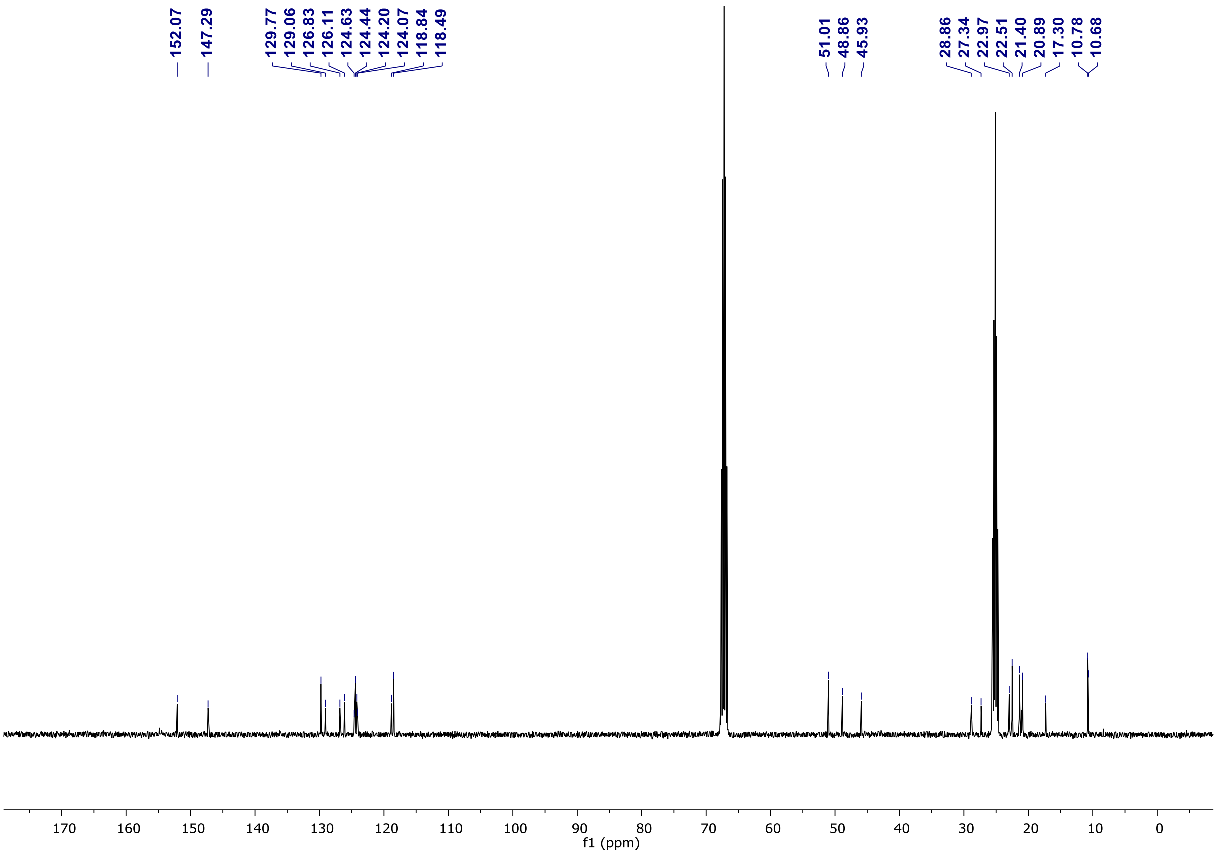

Figure S35. $^{13}C\{^{1}H\}$ NMR spectrum of **10** in THF-d_8.

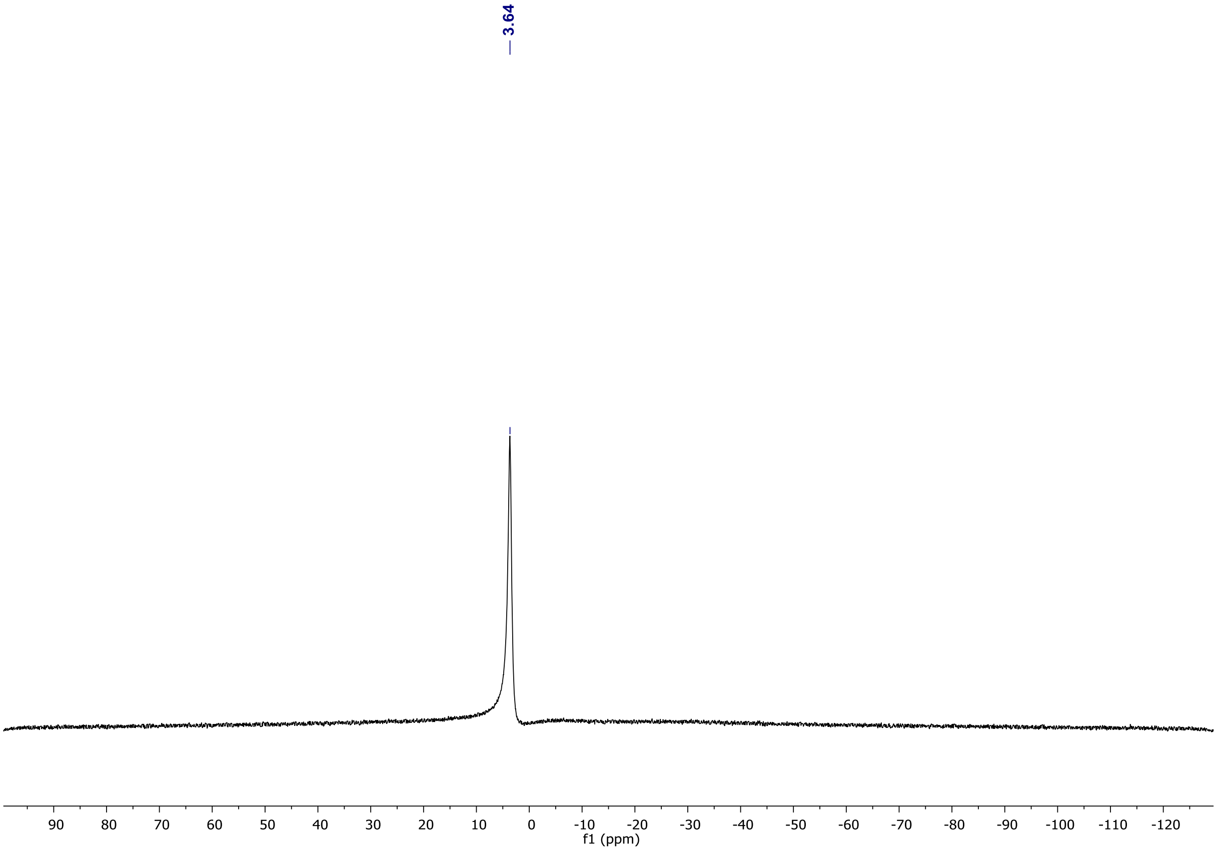

Figure S36. $^{11}B\{^{1}H\}$ NMR spectrum of **10** in THF-d_8.

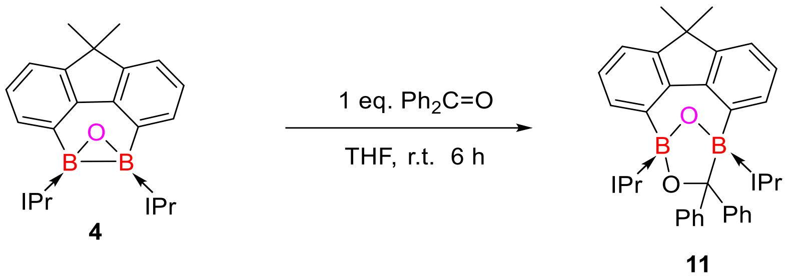

Synthesis of compound 11. THF (50 mL) was added into a 100 mL Schlenk flask containing **4** (177 mg, 0.3 mmol) and benzophenone (55 mg, 0.3 mmol) at room temperature. The reaction mixture was stirred for 6 hours. The resulting colorless solution was filtered. The filtrate was concentrated to afford compound **11** as colorless crystals in 96% yield (290 mg).

M.p.: 185.8 ºC.

^{1}H NMR (400 MHz, Methylene Chloride-d_2) δ/ppm 7.13 (m, 4H, Ar-*H*), 7.00 (m, 3H, Ar-*H*), 6.96 (sept, J = 7.0 Hz, 1H, C*H*(CH_3)$_2$), 6.86 (m, 5H, Ar-*H*), 6.73 (m, 2H, Ar-*H*), 6.53 (sept, J = 7.0 Hz, 1H, C*H*(CH_3)$_2$), 6.37 (m, 1H, Ar-*H*), 6.25 (d, J = 7.0 Hz, 1H, Ar-*H*), 6.12 (sept, J = 7.0 Hz, 1H, C*H*(CH_3)$_2$), 5.65 (d, J = 7.5 Hz, 1H, Ar-*H*), 5.22 (sept, J = 7.0 Hz, 1H, C*H*(CH_3)$_2$), 2.24 (s, 3H, C=CCH_3), 2.20 (s, 3H, C=CCH_3), 2.14 (s, 3H, C=CCH_3), 2.09 (s, 3H, C=CCH_3), 1.51 (d, J = 7.0 Hz, 3H, CH(CH_3)$_2$), 1.48 (s, 3H, C(CH_3)$_2$), 1.38 (s, 3H, C(CH_3)$_2$), 1.36 (d, J = 7.0 Hz, 3H, CH(CH_3)$_2$), 1.32 (d, J = 7.0 Hz, 3H, CH(CH_3)$_2$), 1.26 (d, J = 7.2 Hz, 6H, CH(CH_3)$_2$), 0.74 (d, J = 6.9 Hz, 3H, CH(CH_3)$_2$), 0.59 (d, J = 6.8 Hz, 3H, CH(CH_3)$_2$), 0.16 (d, J = 7.0 Hz, 3H, CH(CH_3)$_2$).

^{13}C{^{1}H} NMR (101 MHz, Methylene Chloride-d_2) δ/ppm 152.88, 152.57, 152.52, 151.73, 146.83, 146.05, 131.99, 128.84, 126.70, 124.48, 124.23, 124.01, 123.53, 123.07, 122.30 (s, Ar-*C*), 118.73 (s, CH_3*C*=*C*CH_3), 116.80 (s, CH_3*C*=*C*CH_3), 50.59 (s, *C*H(CH_3)$_2$), 49.83 (s, *C*H(CH_3)$_2$), 48.79 (s, *C*H(CH_3)$_2$), 47.89 (s, *C*H(CH_3)$_2$), 45.70 (s, Ph_2*C*O), 29.27 (s, *C*(CH_3)$_2$), 27.92 (s, C(*C*H_3)$_2$), 22.87 (s, CH(*C*H_3)$_2$), 22.07 (s, CH(*C*H_3)$_2$), 21.85 (s, CH(*C*H_3)$_2$), 21.79 (s, CH(*C*H_3)$_2$), 21.54 (s, CH(*C*H_3)$_2$), 21.22 (s, CH(*C*H_3)$_2$), 18.88 (s, CH(*C*H_3)$_2$), 11.01 (s, C=C*C*H_3), 10.90 (s, C=C*C*H_3), 10.74 (s, C=C*C*H_3).

^{11}B{^{1}H} NMR (128 MHz, Methylene Chloride-d_2) δ/ppm 5.91, 3.04.

HR-MS (ESI): (m/z) calcd for $[M+H]^+$ ($C_{50}H_{63}B_2N_4O_2^+$) 773.5132; Found: 773.5152.

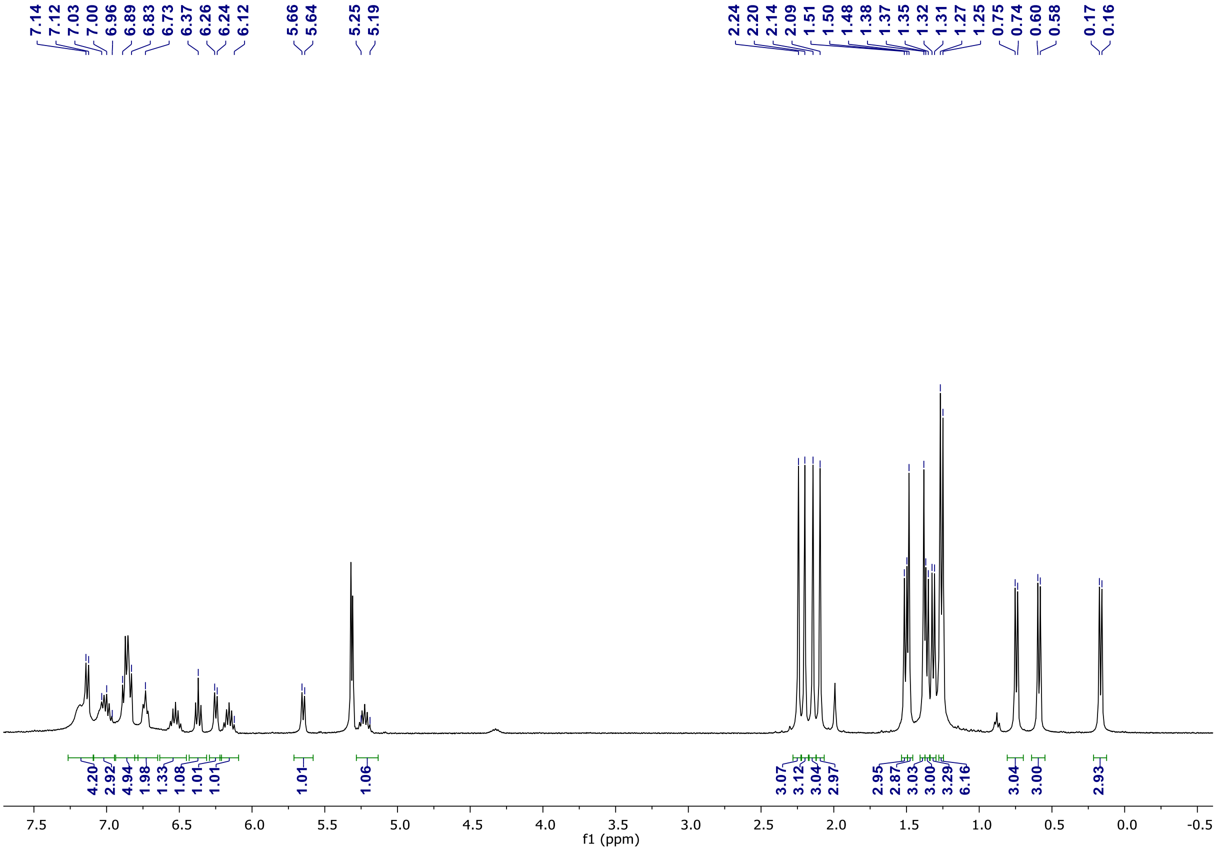

Figure S37. ^{1}H NMR spectrum of **11** in methylene chloride-d_2.

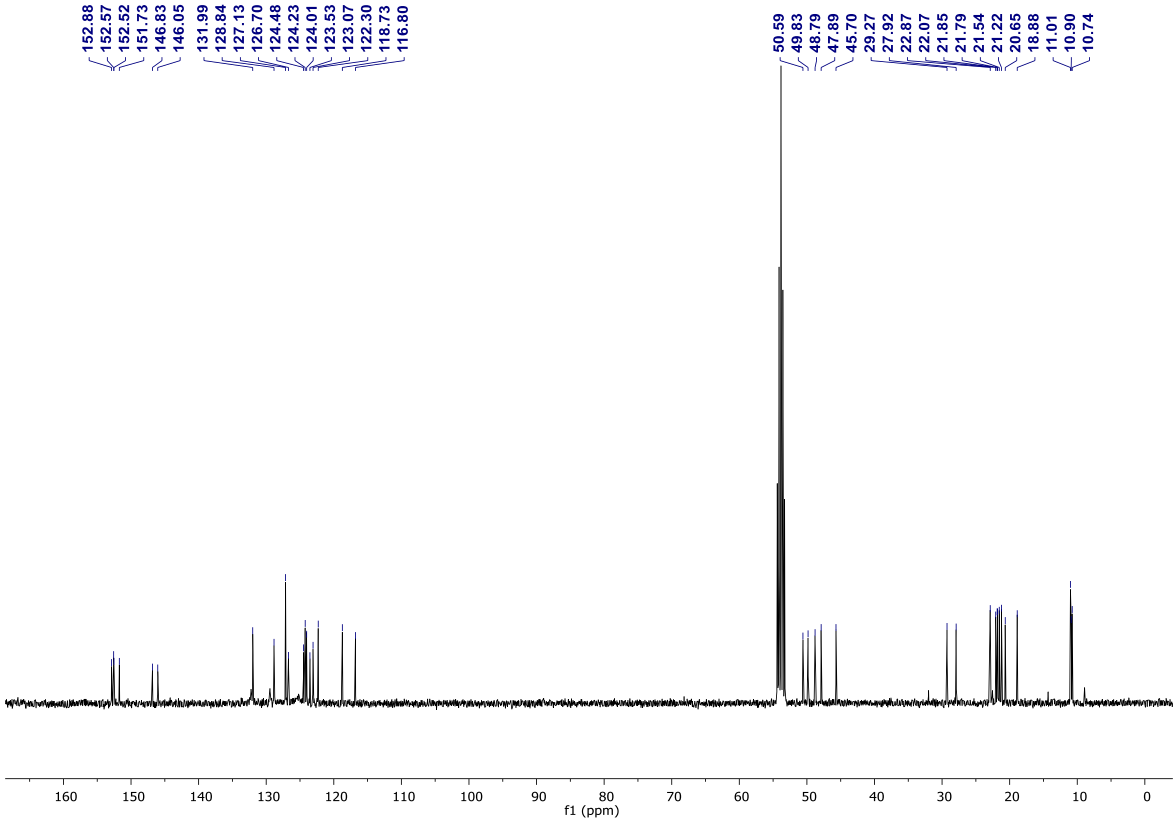

Figure S38. ^{13}C{^{1}H} NMR spectrum of **11** in methylene chloride-d_2.

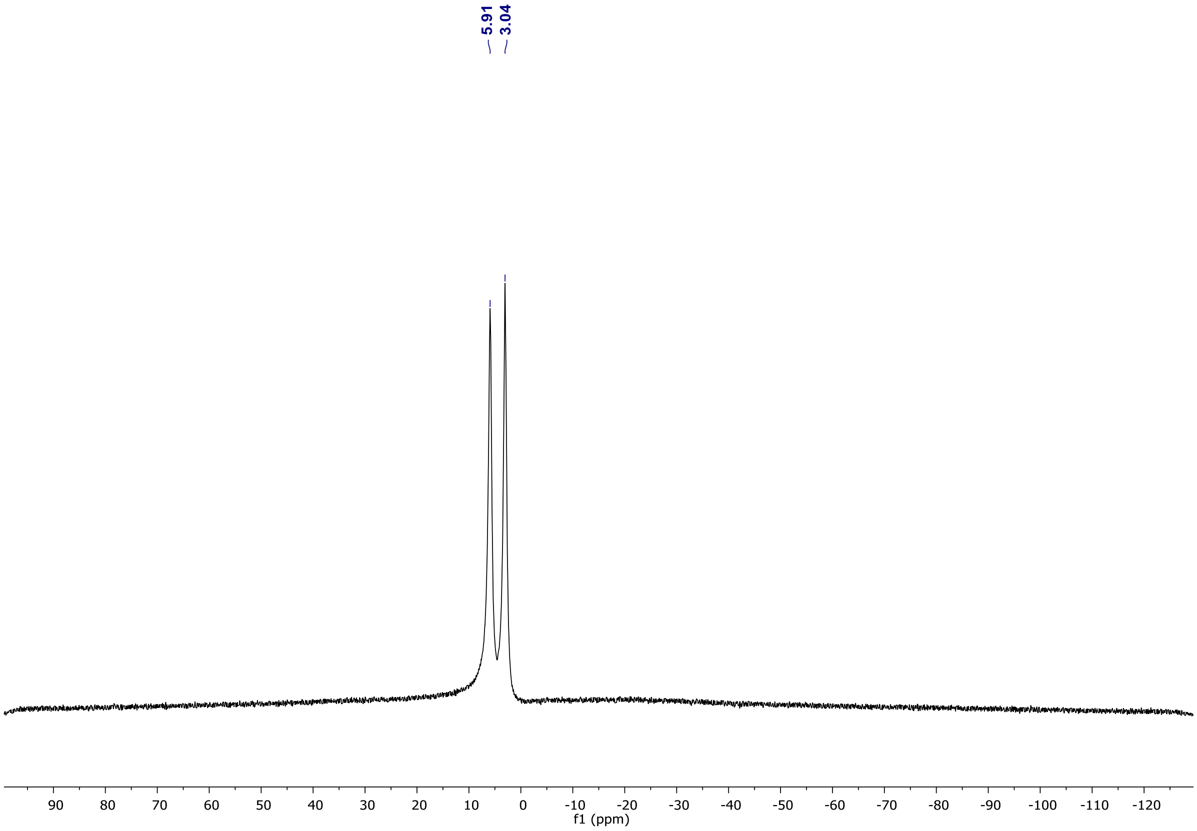

Figure S39. $^{11}B\{^{1}H\}$ NMR spectrum of **11** in methylene chloride-d_2.

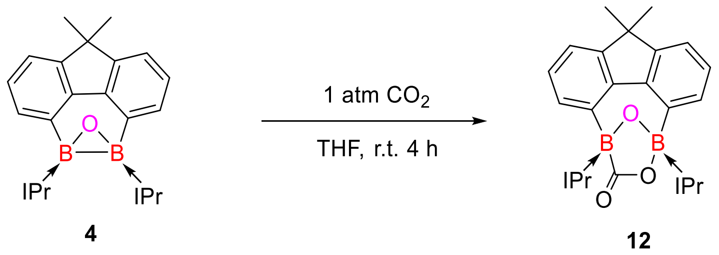

Synthesis of compound 12. After three freeze-pump thaw cycles, the THF solution of compound **4** (177 mg, 0.3 mmol) in a 100 mL Schlenk flask was subjected to 1 atm CO_2 at room temperature under stirring. After 4 hours, the color of the mixture changed from orange-red to yellow. The resulting yellow solution was filtered and concentrated to afford compound **12** as colorless crystals in 74% yield (141 mg).

M.p.: 271.5 °C.

^{1}H NMR (400 MHz, Methylene Chloride-d_2) δ/ppm 7.20 (d, J = 7.4 Hz, 1H, Ar-H), 7.10 (d, J = 7.4 Hz, 1H, Ar-H), 6.94 (m, 2H, Ar-H), 6.68 (sept, J = 6.8 Hz, 1H, CH(CH$_3$)$_2$), 6.55 (d, J = 7.1 Hz, 1H, Ar-H), 6.45 (d, J = 7.2 Hz, 1H, Ar-H), 6.38 (sept, J = 7.0 Hz, 1H, CH(CH$_3$)$_2$), 5.86 (sept, J = 7.0 Hz, 1H, CH(CH$_3$)$_2$), 5.78 (sept, J = 6.8 Hz, 1H, CH(CH$_3$)$_2$), 2.34 (s, 3H, C=CCH_3), 2.33 (s, 3H, C=CCH_3), 2.28 (s, 3H, C=CCH_3), 2.23 (s, 3H, C=CCH_3), 1.66 (d, J = 6.9 Hz, 3H, CH(CH_3)$_2$), 1.52 (6H, CH(CH_3)$_2$, overlapping), 1.45 (s, 3H, C(CH_3)$_2$), 1.41 (6H, C(CH_3)$_2$ and CH(CH_3)$_2$, overlapping), 1.31 (6H, CH(CH_3)$_2$, overlapping), 1.24 (d, J = 7.2 Hz, 3H, CH(CH_3)$_2$), 0.78 (d, J = 7.1 Hz, 3H, CH(CH_3)$_2$).

^{13}C{^{1}H} NMR (101 MHz, Methylene Chloride-d_2) δ/ppm 153.28, 145.60, 144.51, 129.93, 129.62, 126.29, 125.61, 125.51, 124.68, 124.13, 123.79 (s, Ar-C), 119.78 (s, CH$_3$$C$=$CCH_3$), 118.71 (s, CH$_3$$C$=$CCH_3$), 51.58 (s, CH(CH$_3$)$_2$), 50.73 (s, CH(CH$_3$)$_2$), 50.08 (s, CH(CH$_3$)$_2$), 49.82 (s, CH(CH$_3$)$_2$), 46.15 (s, C(CH$_3$)$_2$), 28.33 (s, C(CH$_3$)$_2$), 28.04 (s, C(CH$_3$)$_2$), 22.48 (s, CH(CH$_3$)$_2$), 22.38 (s, CH(CH$_3$)$_2$), 22.26 (s, CH(CH$_3$)$_2$), 22.09 (s, CH(CH$_3$)$_2$), 21.68 (s, CH(CH$_3$)$_2$), 21.65 (s, CH(CH$_3$)$_2$), 21.44 (s, CH(CH$_3$)$_2$), 20.12 (s, CH(CH$_3$)$_2$), 11.11 (s, C=CCH$_3$), 11.04 (s, C=CCH$_3$), 11.08 (s, C=CCH$_3$), 10.88 (s, C=CCH$_3$).

^{11}B{^{1}H} NMR (128 MHz, Methylene Chloride-d_2) δ/ppm 6.77, -3.95.

IR (cm^{-1}): 1638.96 (s, OC=O).

HR-MS (ESI): (m/z) calcd for [M+H]$^+$ ($C_{38}H_{53}B_2N_4O_3^+$) 635.4298; Found: 635.4310.

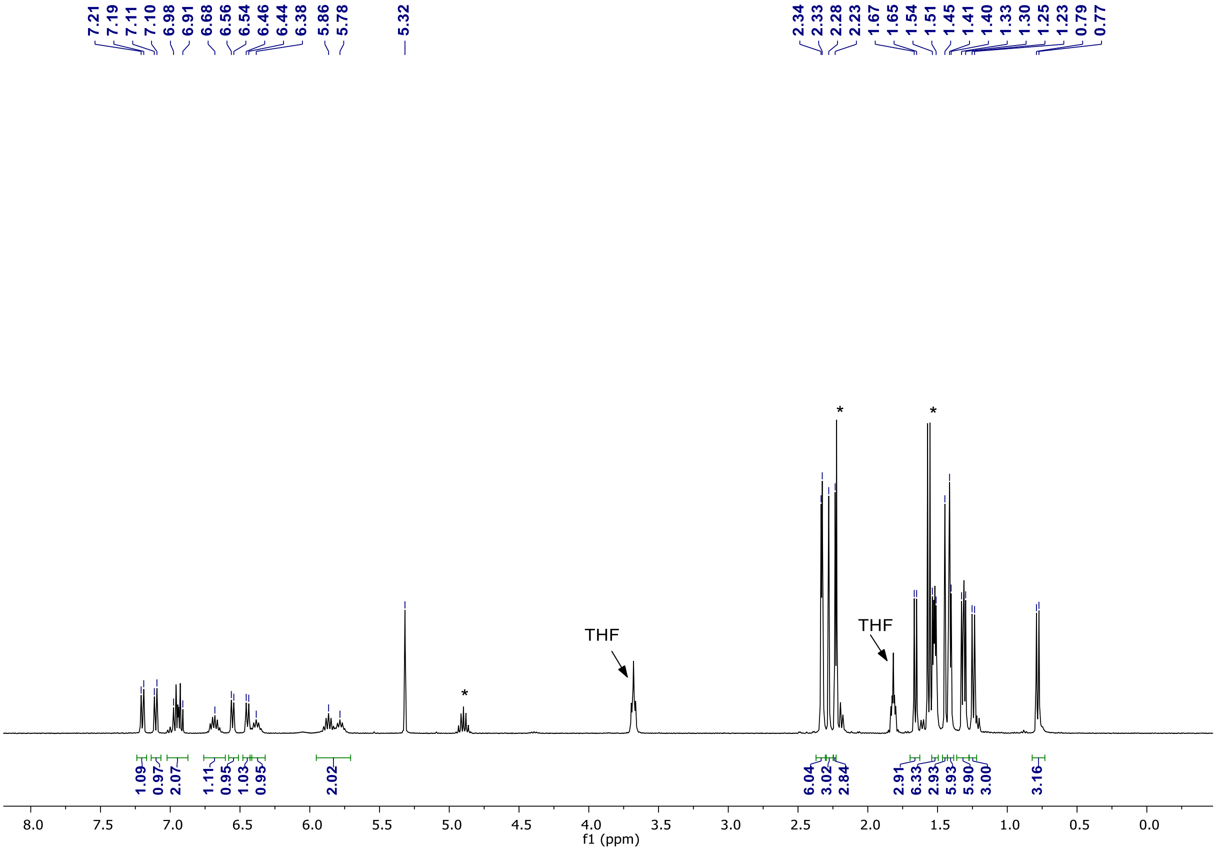

Figure S40. ^{1}H NMR spectrum of **12** in methylene chloride-d_2. * is ^{iPr}NHC-CO_2.

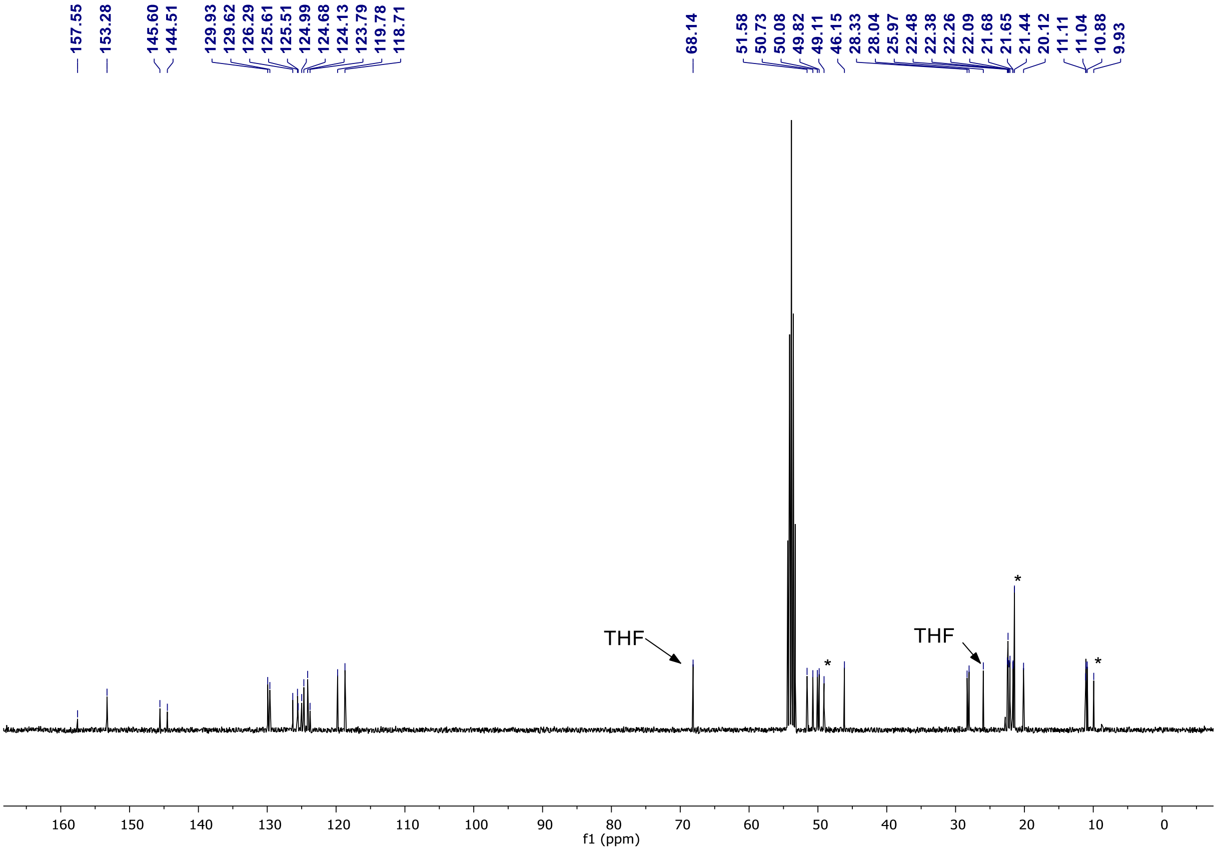

Figure S41. $^{13}C\{^{1}H\}$ NMR spectrum of **12** in methylene chloride-d_2. * is ^{iPr}NHC-CO_2.

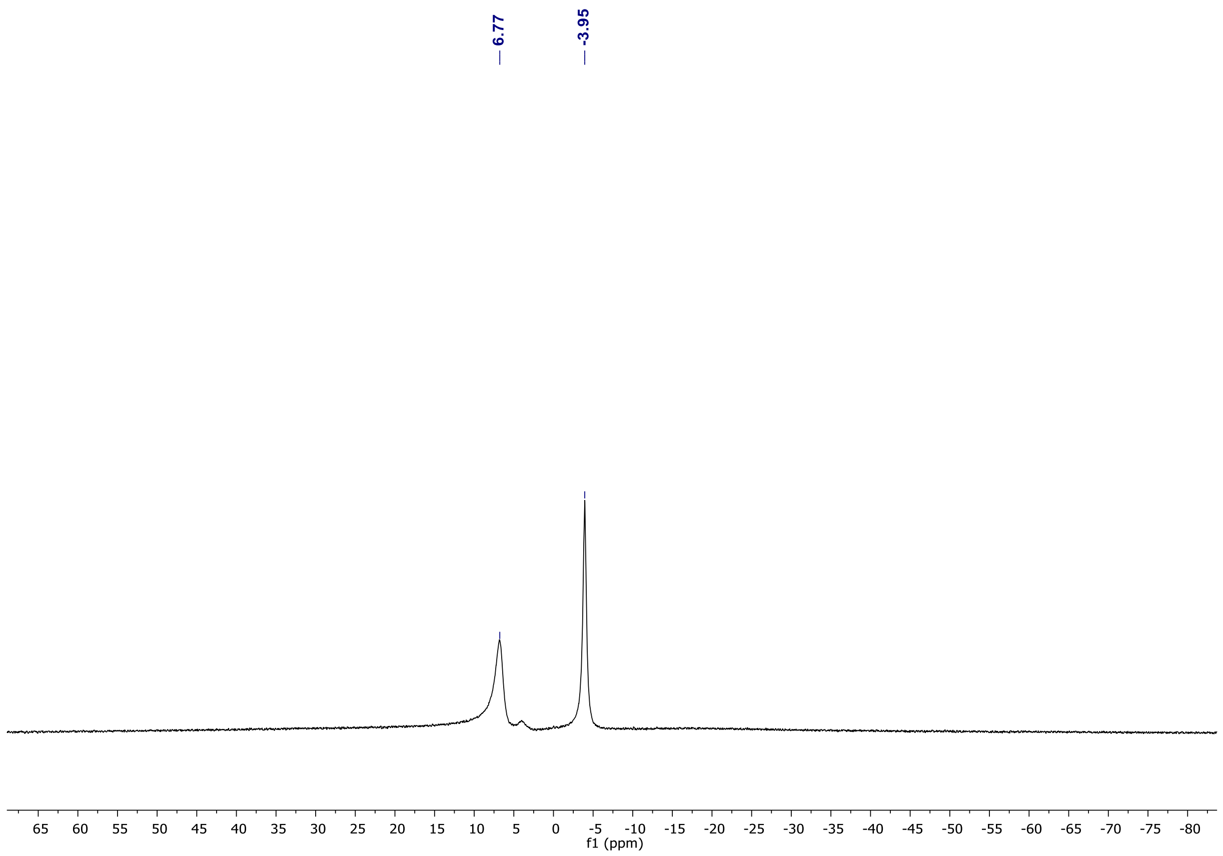

Figure S42. $^{11}B\{^{1}H\}$ NMR spectrum of **12** in methylene chloride-d_2.

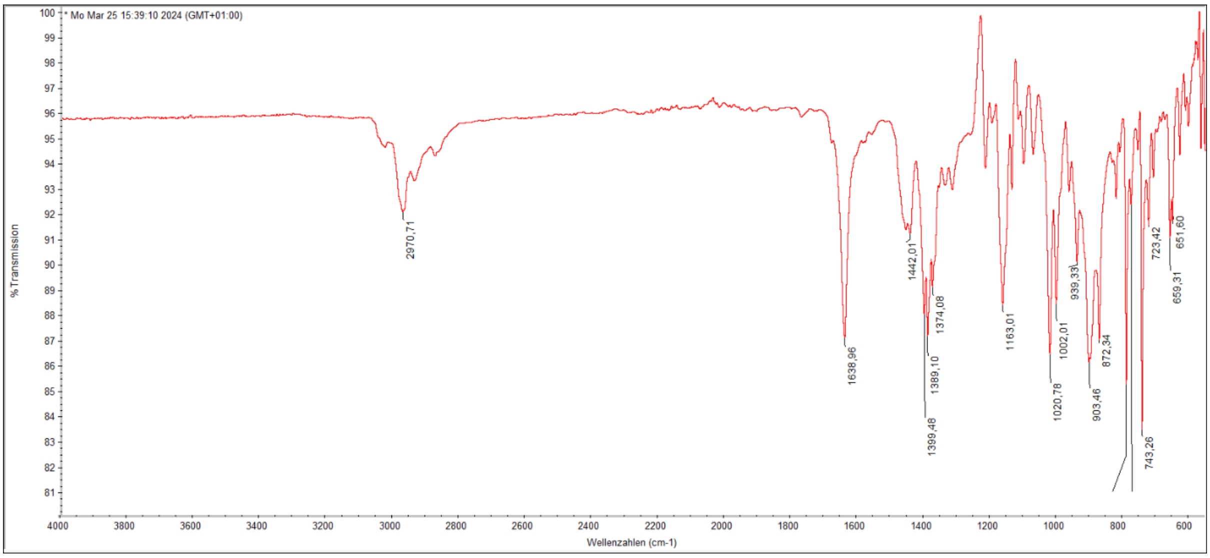

Figure S43. IR spectrum of **12**.

C. X-ray Crystallographic Data

Table S1. Crystal data and structure refinement for **1**.

Empirical formula	C83 H112 B2 Cl4 N4 O4.5	
Formula weight	1401.18	
Temperature	150.00(10) K	
Wavelength	1.54184 Å	
Crystal system	Orthorhombic	
Space group	Pbca	
Unit cell dimensions	a = 17.0596(4) Å	a= 90°.
	b = 29.4425(7) Å	b= 90°.
	c = 31.2643(7) Å	g = 90°.
Volume	15703.3(6) $Å^3$	
Z	8	
Density (calculated)	1.185 Mg/m^3	
Absorption coefficient	1.765 mm^{-1}	
F(000)	6016	
Crystal size	0.09 x 0.04 x 0.02 mm^3	
Theta range for data collection	2.827 to 72.439°.	
Index ranges	-20<=h<=20, -36<=k<=20, -38<=l<=37	
Reflections collected	60683	
Independent reflections	15268 [R(int) = 0.1145]	
Completeness to theta = 67.684°	100.0 %	
Absorption correction	Semi-empirical from equivalents	
Max. and min. transmission	1.00000 and 0.19037	
Refinement method	Full-matrix least-squares on F^2	
Data / restraints / parameters	15268 / 0 / 739	
Goodness-of-fit on F^2	1.057	
Final R indices [I>2sigma(I)]	R1 = 0.1056, wR2 = 0.2706	
R indices (all data)	R1 = 0.1395, wR2 = 0.2931	
Extinction coefficient	n/a	
Largest diff. peak and hole	0.370 and -0.435 e.$Å^{-3}$	

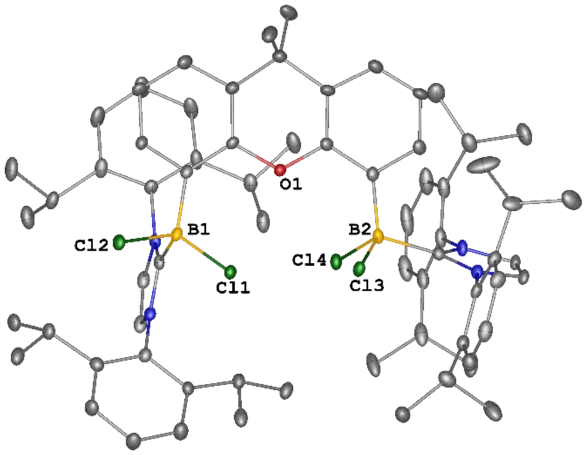

Figure S44. Molecular structure of compound **1**. Thermal ellipsoids are drawn at the 50% probability level. H atoms are omitted for clarity.

Table S2. Selected interatomic distances and angles of compound **1.**

Bond lengths [Å]		Angles [°]	
Cl(1)-B(1)	1.839(6)	Cl(1)-B(1)-Cl(2)	106.1(3)
Cl(2)-B(1)	1.917(6)	C(1)-B(1)-Cl(1)	115.6(4)
Cl(3)-B(2)	1.879(6)		
Cl(4)-B(2)	1.870(6)		

Table S3. Crystal data and structure refinement for **2**.

Empirical formula	C89 H124 B2 N4 O6	
Formula weight	1367.53	
Temperature	150.01(10) K	
Wavelength	1.54184 Å	
Crystal system	Triclinic	
Space group	P-1	
Unit cell dimensions	a = 14.3525(5) Å	a= 67.760(3)°.
	b = 16.6482(6) Å	b= 72.450(3)°.
	c = 20.0433(7) Å	g = 83.415(3)°.
Volume	4226.6(3) $Å^3$	
Z	2	
Density (calculated)	0.791 Mg/m^3	
Absorption coefficient	0.347 mm^{-1}	
F(000)	1088	
Crystal size	0.2 x 0.12 x 0.04 mm^3	
Theta range for data collection	2.481 to 72.422°.	
Index ranges	-17<=h<=16, -20<=k<=20, -24<=l<=24	
Reflections collected	32727	
Independent reflections	16263 [R(int) = 0.0316]	
Completeness to theta = 67.684°	99.7 %	
Absorption correction	Semi-empirical from equivalents	
Max. and min. transmission	1.00000 and 0.83625	
Refinement method	Full-matrix least-squares on F^2	
Data / restraints / parameters	16263 / 0 / 703	
Goodness-of-fit on F^2	1.290	
Final R indices [I>2sigma(I)]	R1 = 0.0954, wR2 = 0.3040	
R indices (all data)	R1 = 0.1112, wR2 = 0.3261	
Extinction coefficient	n/a	
Largest diff. peak and hole	0.476 and -0.543 e.$Å^{-3}$	

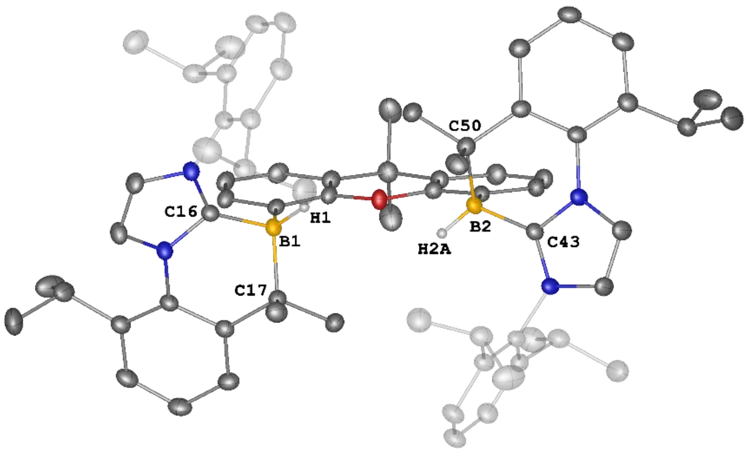

Figure S45. Molecular structure of compound **2**. Thermal ellipsoids are drawn at the 50% probability level. H atoms are omitted for clarity.

Table S4. Selected interatomic distances and angles of compound **2.**

Bond lengths [Å]		Angles [°]	
B(1)-H(1)	1.0000	C(16)-B(1)-C(17)	101.51(19)
B(2)-H(2)	1.0000	C(16)-B(1)-H(1)	110.2
C(16)-B(1)	1.605(3)	C(17)-B(1)-H(1)	110.2
C(50)-B(2)	1.668(4)		

Table S5. Crystal data and structure refinement for **3**.

Empirical formula	C44 H60 B2 Cl4 N4 O	
Formula weight	824.38	
Temperature	149.99(16) K	
Wavelength	1.54184 Å	
Crystal system	Orthorhombic	
Space group	Pbca	
Unit cell dimensions	a = 16.72550(10) Å	a= 90°.
	b = 17.13830(10) Å	b= 90°.
	c = 30.5566(2) Å	g = 90°.
Volume	8758.95(9) $Å^3$	
Z	8	
Density (calculated)	1.250 Mg/m^3	
Absorption coefficient	2.743 mm^{-1}	
F(000)	3504	
Crystal size	0.14 x 0.13 x 0.07 mm^3	
Theta range for data collection	2.892 to 72.556°.	
Index ranges	-19<=h<=20, -15<=k<=21, -37<=l<=37	
Reflections collected	63606	
Independent reflections	8637 [R(int) = 0.0229]	
Completeness to theta = 67.684°	100.0 %	
Absorption correction	Semi-empirical from equivalents	
Max. and min. transmission	1.00000 and 0.56922	
Refinement method	Full-matrix least-squares on F^2	
Data / restraints / parameters	8637 / 0 / 511	
Goodness-of-fit on F^2	1.037	
Final R indices [I>2sigma(I)]	R1 = 0.0283, wR2 = 0.0745	
R indices (all data)	R1 = 0.0307, wR2 = 0.0764	
Extinction coefficient	n/a	
Largest diff. peak and hole	0.355 and -0.322 e.$Å^{-3}$	

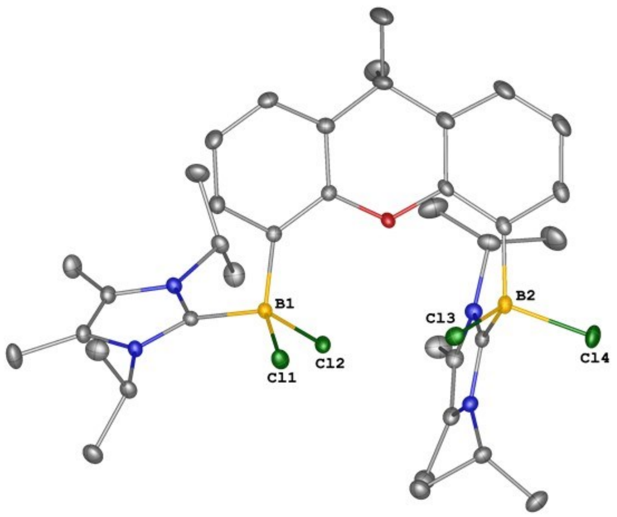

Figure S46. Molecular structure of compound **3**. Thermal ellipsoids are drawn at the 50% probability level. H atoms and solvent toluene are omitted for clarity.

Table S6. Selected interatomic distances and angles of compound **3.**

Bond lengths [Å]		Angles [°]	
Cl(1)-B(1)	1.8829(13)	Cl(1)-B(1)-Cl(2)	105.95(6)
Cl(2)-B(1)	1.8864(13)		
Cl(4)-B(2)	1.9214(13)		
Cl(3)-B(2)	1.8809(14)		

Table S7. Crystal data and structure refinement for **4**.

Empirical formula	C37 H52 B2 N4 O	
Formula weight	590.44	
Temperature	150.00 K	
Wavelength	1.54184 Å	
Crystal system	Monoclinic	
Space group	P 1 21/n 1	
Unit cell dimensions	a = 10.8079(13) Å	a= 90°.
	b = 21.795(2) Å	b= 95.652(12)°.
	c = 14.4606(17) Å	g = 90°.
Volume	3389.8(7) Å^3	
Z	4	
Density (calculated)	1.157 Mg/m^3	
Absorption coefficient	0.524 mm^{-1}	
F(000)	1280	
Crystal size	0.14 x 0.12 x 0.015 mm^3	
Theta range for data collection	3.681 to 67.488°.	
Index ranges	-12<=h<=12, -23<=k<=26, -17<=l<=11	
Reflections collected	13143	
Independent reflections	6106 [R(int) = 0.0852]	
Completeness to theta = 67.488°	99.9 %	
Absorption correction	Semi-empirical from equivalents	
Max. and min. transmission	1.00000 and 0.58939	
Refinement method	Full-matrix least-squares on F^2	
Data / restraints / parameters	6106 / 0 / 421	
Goodness-of-fit on F^2	1.049	
Final R indices [I>2sigma(I)]	R1 = 0.1156, wR2 = 0.3074	
R indices (all data)	R1 = 0.1581, wR2 = 0.3481	
Extinction coefficient	n/a	
Largest diff. peak and hole	0.345 and -0.392 e.Å^{-3}	

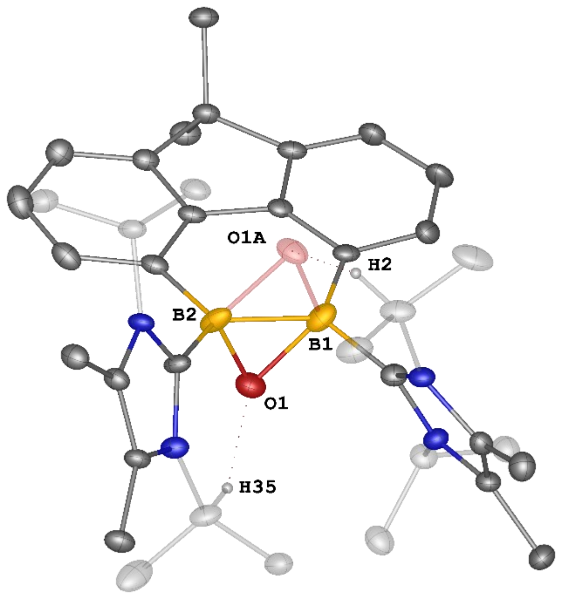

Figure S47. Molecular structure of compound **4**. Thermal ellipsoids are drawn at the 50% probability level. H atoms are omitted for clarity. The entire molecule is disordered over two orientations with a ratio of 32:68%. Despite multiple recrystallizations of this compound, significant molecular disorder persists. As a result, the data is sufficient solely for structure confirmation, and the selected bond lengths and bond angles should be considered for reference purposes only.

Table S8. Selected interatomic distances and angles of compound **4.**

Bond lengths [Å]		Angles [°]	
B(1)-B(2)	1.725(10)	B(2)-O(1)-B(1)	64.5(4)
B(1)-O(1)	1.662(10)	B(1)-O(1A)-B(2)	61.5(5)
B(2)-O(1)	1.566(10)	O(1)-B(2)-B(1)	60.5(4)
B(1)-O(1A)	1.599(14)	O(1)-B(1)-B(2)	55.0(4)
B(2)-O(1A)	1.765(15)	O(1A)-B(1)-B(2)	64.0(6)
		B(1)-B(2)-O(1A)	54.5(6)

Table S9. Crystal data and structure refinement for **5**.

Empirical formula	C36 H44 B2 Cl4 N4 O	
Formula weight	712.17	
Temperature	150.00(10) K	
Wavelength	1.54184 Å	
Crystal system	Triclinic	
Space group	P-1	
Unit cell dimensions	a = 11.0879(13) Å	a= 110.927(13)°.
	b = 13.0235(18) Å	b= 110.426(13)°.
	c = 15.314(2) Å	g = 94.139(11)°.
Volume	1886.1(5) $Å^3$	
Z	2	
Density (calculated)	1.254 Mg/m^3	
Absorption coefficient	3.107 mm^{-1}	
F(000)	748	
Crystal size	0.11 x 0.05 x 0.02 mm^3	
Theta range for data collection	3.375 to 72.288°.	
Index ranges	-13<=h<=13, -15<=k<=16, -18<=l<=17	
Reflections collected	13351	
Independent reflections	7240 [R(int) = 0.1763]	
Completeness to theta = 67.684°	99.7 %	
Absorption correction	Semi-empirical from equivalents	
Max. and min. transmission	1.00000 and 0.29682	
Refinement method	Full-matrix least-squares on F^2	
Data / restraints / parameters	7240 / 0 / 435	
Goodness-of-fit on F^2	0.934	
Final R indices [I>2sigma(I)]	R1 = 0.0877, wR2 = 0.1414	
R indices (all data)	R1 = 0.2176, wR2 = 0.2184	
Extinction coefficient	n/a	
Largest diff. peak and hole	0.410 and -0.391 e.$Å^{-3}$	

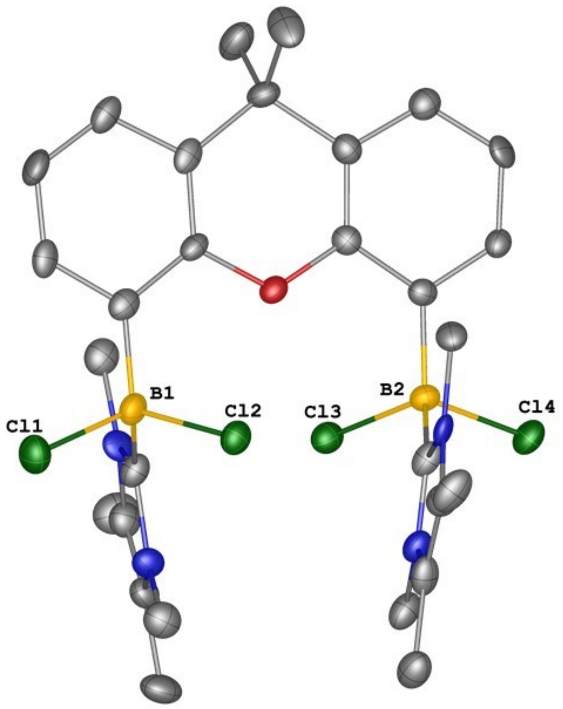

Figure S48. Molecular structure of compound **5**. Thermal ellipsoids are drawn at the 50% probability level. H atoms and solvent toluene are omitted for clarity. Despite multiple recrystallizations of this compound, the data is sufficient solely for structure confirmation, and the selected bond lengths and bond angles should be considered for reference purposes only.

Table S10. Selected interatomic distances and angles of compound **5.**

Bond lengths [Å]		Angles [°]	
Cl(1)-B(1)	1.896(9)	Cl(3)-B(2)-Cl(4)	104.6(4)
Cl(2)-B(1)	1.891(9)	Cl(2)-B(1)-Cl(1)	105.3(5)
Cl(3)-B(2)	1.901(9)		
Cl(4)-B(2)	1.905(9)		

Table S11. Crystal data and structure refinement for **6**.

Empirical formula	C57 H76 B2 N8 O	
Formula weight	910.941	
Temperature	150(10) K	
Wavelength	1.54184 Å	
Crystal system	Orthorhombic	
Space group	Pccn	
Unit cell dimensions	a = 10.4605(2) Å	a= 90°.
	b = 19.3937(4) Å	b= 90°.
	c = 25.5988(4) Å	g = 90°.
Volume	5193.17(17) $Å^3$	
Z	4	
Density (calculated)	1.165 Mg/m^3	
Absorption coefficient	0.535 mm^{-1}	
F(000)	1973.705	
Crystal size	0.04 x 0.03 x 0.02 mm^3	
Theta range for data collection	3.45 to 67.50°.	
Index ranges	-12<=h<=12, -23<=k<=23, -31<=l<=28	
Reflections collected	20524	
Independent reflections	4685 [R(int) = 0.0266]	
Completeness to theta = 67.4975°	99.89 %	
Absorption correction	Semi-empirical from equivalents	
Max. and min. transmission	1.00000 and 0.40739	
Refinement method	Full-matrix least-squares on F^2	
Data / restraints / parameters	4685 / 0 / 318	
Goodness-of-fit on F^2	1.0455	
Final R indices [I>2sigma(I)]	R1 = 0.0388, wR2 = 0.0973	
R indices (all data)	R1 = 0.0471, wR2 = 0.1046	
Largest diff. peak and hole	0.1808 and -0.2609 e.$Å^{-3}$	

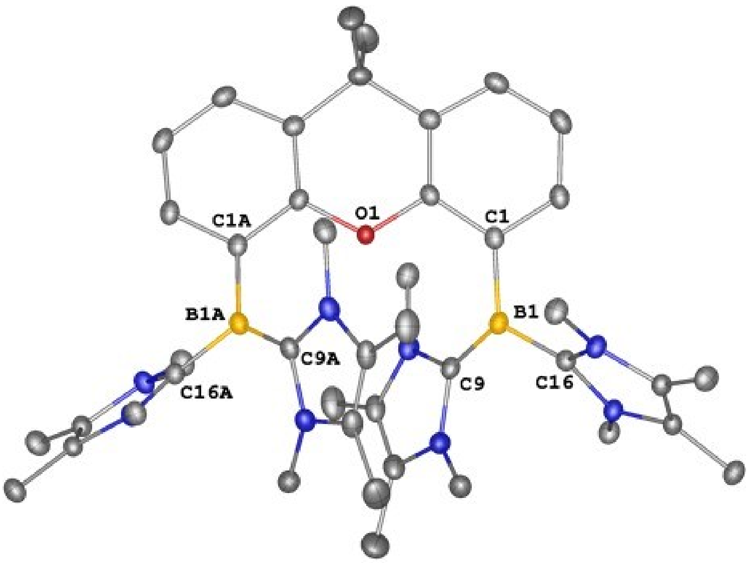

Figure S49. Molecular structure of compound **6**. Thermal ellipsoids are drawn at the 50% probability level. H atoms and solvent toluene are omitted for clarity.

Table S12. Selected interatomic distances and angles of compound **6.**

Bond lengths [Å]		Angles [°]	
C(1)-B(1)	1.5773(19)	C(9)-B(1)-C(16)	117.38(11)
C(9)-B(1)	1.5114(19)	C(1)-B(1)-C(16)	116.02(11)
C(16)-B(1)	1.5327(19)	C(1)-B(1)-C(9)	125.78(11)

Table S13. Crystal data and structure refinement for **7.**

Empirical formula	C58 H76 B2 N4 O2	
Formula weight	882.84	
Temperature	150.00(1) K	
Wavelength	1.54184 Å	
Crystal system	Tetragonal	
Space group	$I4_1md$	
Unit cell dimensions	a = 14.8723(4) Å	a= 90°.
	b = 14.8723(4) Å	b= 90°.
	c = 24.3596(10) Å	g = 90°.
Volume	5388.0(4) $Å^3$	
Z	4	
Density (calculated)	1.088 Mg/m^3	
Absorption coefficient	0.493 mm^{-1}	
F(000)	1912	
Crystal size	0.17 x 0.12 x 0.02 mm^3	
Theta range for data collection	3.482 to 72.390°.	
Index ranges	-18<=h<=17, -18<=k<=16, -29<=l<=28	
Reflections collected	10637	
Independent reflections	2667 [R(int) = 0.0559]	
Completeness to theta = 67.684°	99.6 %	
Absorption correction	Semi-empirical from equivalents	
Max. and min. transmission	1.00000 and 0.34560	
Refinement method	Full-matrix least-squares on F^2	
Data / restraints / parameters	2667 / 1 / 125	
Goodness-of-fit on F^2	1.002	
Final R indices [I>2sigma(I)]	R1 = 0.0493, wR2 = 0.1234	
R indices (all data)	R1 = 0.0606, wR2 = 0.1312	
Absolute structure parameter	0.5(3)	
Extinction coefficient	n/a	
Largest diff. peak and hole	0.133 and -0.187 e.$Å^{-3}$	

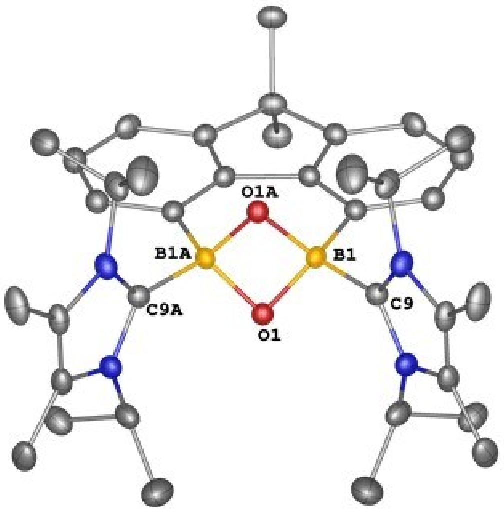

Figure S50. Molecular structure of compound **7**. Thermal ellipsoids are drawn at the 50% probability level. H atoms are omitted for clarity.

Table S14. Selected interatomic distances and angles of compound **7.**

Bond lengths [Å]		Angles [°]	
O(1)-B(1)	1.484(3)	B(1)-O(1)-B(1A)	83.2(3)
O(1)-B(1A)	1.484(3)	O(1)-B(1)-O(1A)	95.3(3)
C(9)-B(1)	1.648(5)		

Table S15. Crystal data and structure refinement for **8**.

Empirical formula	C37 H52 B2 N4 O3	
Formula weight	622.44	
Temperature	149.97(10) K	
Wavelength	1.54184 Å	
Crystal system	Monoclinic	
Space group	P 1 21/n 1	
Unit cell dimensions	a = 10.2759(11) Å	a= 90°.
	b = 25.442(3) Å	b= 91.060(10)°.
	c = 13.1324(16) Å	g = 90°.
Volume	3432.7(7) Å^3	
Z	4	
Density (calculated)	1.204 Mg/m^3	
Absorption coefficient	0.588 mm^{-1}	
F(000)	1344	
Crystal size	0.08 x 0.07 x 0.05 mm^3	
Theta range for data collection	3.474 to 72.788°.	
Index ranges	-12<=h<=12, -30<=k<=21, -15<=l<=11	
Reflections collected	13185	
Independent reflections	6623 [R(int) = 0.0621]	
Completeness to theta = 67.684°	99.9 %	
Absorption correction	Semi-empirical from equivalents	
Max. and min. transmission	1.00000 and 0.48586	
Refinement method	Full-matrix least-squares on F^2	
Data / restraints / parameters	6623 / 0 / 429	
Goodness-of-fit on F^2	1.019	
Final R indices [I>2sigma(I)]	R1 = 0.0648, wR2 = 0.1441	
R indices (all data)	R1 = 0.1178, wR2 = 0.1765	
Extinction coefficient	n/a	
Largest diff. peak and hole	0.313 and -0.268 e.Å^{-3}	

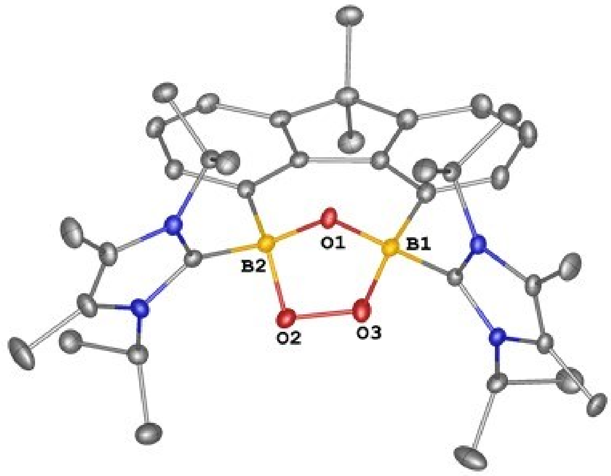

Figure S51. Molecular structure of compound **8**. Thermal ellipsoids are drawn at the 50% probability level. H atoms are omitted for clarity.

Table S16. Selected interatomic distances and angles of compound **8.**

Bond lengths [Å]		Angles [°]	
B(2)-O(1)	1.446(4)	O(1)-B(1)-O(3)	106.2(2)
B(2)-O(2)	1.491(4)	O(1)-B(2)-O(2)	105.2(2)
O(2)-O(3)	1.437(3)	B(2)-O(1)-B(1)	103.9(2)
B(1)-O(1)	1.451(4)	O(3)-O(2)-B(2)	102.1(2)
B(1)-O(3)	1.504(4)	O(2)-O(3)-B(1)	107.1(2)

Table S17. Crystal data and structure refinement for **9**.

Empirical formula	C37 H52 B2 N4 O S2	
Formula weight	654.56	
Temperature	129(30) K	
Wavelength	1.54184 Å	
Crystal system	Monoclinic	
Space group	P 1 21/c 1	
Unit cell dimensions	a = 19.8515(5) Å	a= 90°.
	b = 10.2266(2) Å	b= 99.414(2)°.
	c = 18.2895(4) Å	g = 90°.
Volume	3663.01(14) Å^3	
Z	4	
Density (calculated)	1.187 Mg/m^3	
Absorption coefficient	1.570 mm^{-1}	
F(000)	1408	
Crystal size	0.15 x 0.05 x 0.02 mm^3	
Theta range for data collection	4.516 to 67.498°.	
Index ranges	-23<=h<=23, 0<=k<=12, 0<=l<=21	
Reflections collected	6597	
Independent reflections	6597 [R(int) = ?]	
Completeness to theta = 67.498°	99.9 %	
Absorption correction	Semi-empirical from equivalents	
Max. and min. transmission	1.00000 and 0.68083	
Refinement method	Full-matrix least-squares on F^2	
Data / restraints / parameters	6597 / 0 / 430	
Goodness-of-fit on F^2	1.640	
Final R indices [I>2sigma(I)]	R1 = 0.1058, wR2 = 0.3410	
R indices (all data)	R1 = 0.1100, wR2 = 0.3511	
Extinction coefficient	n/a	
Largest diff. peak and hole	1.141 and -0.592 e.Å^{-3}	

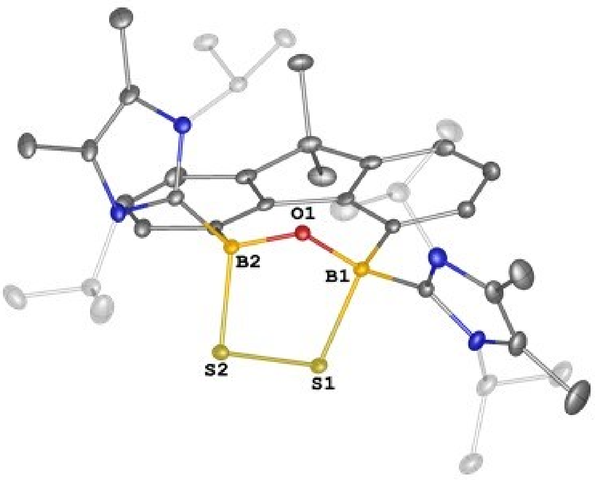

Figure S52. Molecular structure of compound **9**. Thermal ellipsoids are drawn at the 50% probability level. H atoms are omitted for clarity.

Table S18. Selected interatomic distances and angles of compound **9.**

Bond lengths [Å]		Angles [°]	
S(1)-S(2)	2.0688(14)	B(1)-S(1)-S(2)	96.17(14)
S(1)-B(1)	2.049(5)	B(2)-S(2)-S(1)	88.92(14)
S(2)-B(2)	1.990(5)	B(2)-O(1)-B(1)	118.2(3)
O(1)-B(1)	1.437(5)		
O(1)-B(2)	1.428(5)		

Table S19. Crystal data and structure refinement for **10**.

Empirical formula	C46 H61 B2 N5 O	
Formula weight	721.61	
Temperature	149.98(11) K	
Wavelength	1.54184 Å	
Crystal system	Triclinic	
Space group	P-1	
Unit cell dimensions	a = 10.5066(13) Å	a= 79.749(12)°.
	b = 14.0435(19) Å	b= 85.024(11)°.
	c = 14.450(2) Å	g = 89.071(10)°.
Volume	2090.2(5) Å^3	
Z	2	
Density (calculated)	1.147 Mg/m^3	
Absorption coefficient	0.519 mm^{-1}	
F(000)	780	
Crystal size	0.06 x 0.05 x 0.02 mm^3	
Theta range for data collection	3.119 to 72.532°.	
Index ranges	-12<=h<=11, -16<=k<=17, -17<=l<=15	
Reflections collected	14826	
Independent reflections	8015 [R(int) = 0.1126]	
Completeness to theta = 67.684°	99.8 %	
Absorption correction	Semi-empirical from equivalents	
Max. and min. transmission	1.00000 and 0.49261	
Refinement method	Full-matrix least-squares on F^2	
Data / restraints / parameters	8015 / 3 / 505	
Goodness-of-fit on F^2	1.052	
Final R indices [I>2sigma(I)]	R1 = 0.1111, wR2 = 0.2637	
R indices (all data)	R1 = 0.2040, wR2 = 0.3527	
Extinction coefficient	n/a	
Largest diff. peak and hole	0.661 and -0.361 e.Å^{-3}	

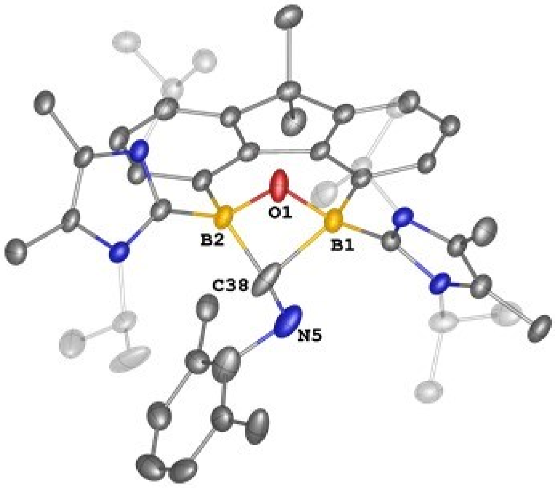

Figure S53. Molecular structure of compound **10**. Thermal ellipsoids are drawn at the 50% probability level. H atoms are omitted for clarity.

Table S20. Selected interatomic distances and angles of compound **10.**

Bond lengths [Å]		Angles [°]	
O(1)-B(1)	1.485(7)	B(2)-C(38)-B(1)	79.6(5)
O(1)-B(2)	1.488(8)	O(1)-B(1)-C(38)	82.3(4)
B(1)-C(38)	1.910(12)	B(1)-O(1)-B(2)	99.7(4)
B(2)-C(38)	1.621(9)	O(1)-B(2)-C(38)	92.9(5)

Table S21. Crystal data and structure refinement for **11**.

Empirical formula	C51 H64 B2 Cl2 N4 O2	
Formula weight	857.58	
Temperature	149.95(10) K	
Wavelength	1.54184 Å	
Crystal system	Orthorhombic	
Space group	Pbca	
Unit cell dimensions	a = 20.8586(2) Å	a= 90°.
	b = 20.4189(2) Å	b= 90°.
	c = 21.4397(2) Å	g = 90°.
Volume	9131.38(15) $Å^3$	
Z	8	
Density (calculated)	1.248 Mg/m^3	
Absorption coefficient	1.620 mm^{-1}	
F(000)	3664	
Crystal size	0.24 x 0.12 x 0.04 mm^3	
Theta range for data collection	3.664 to 72.528°.	
Index ranges	-25<=h<=20, -25<=k<=24, -26<=l<=25	
Reflections collected	66415	
Independent reflections	9016 [R(int) = 0.0388]	
Completeness to theta = 67.684°	100.0 %	
Absorption correction	Semi-empirical from equivalents	
Max. and min. transmission	1.00000 and 0.65271	
Refinement method	Full-matrix least-squares on F^2	
Data / restraints / parameters	9016 / 0 / 564	
Goodness-of-fit on F^2	1.039	
Final R indices [I>2sigma(I)]	R1 = 0.0452, wR2 = 0.1215	
R indices (all data)	R1 = 0.0517, wR2 = 0.1284	
Extinction coefficient	n/a	
Largest diff. peak and hole	0.550 and -0.894 e.$Å^{-3}$	

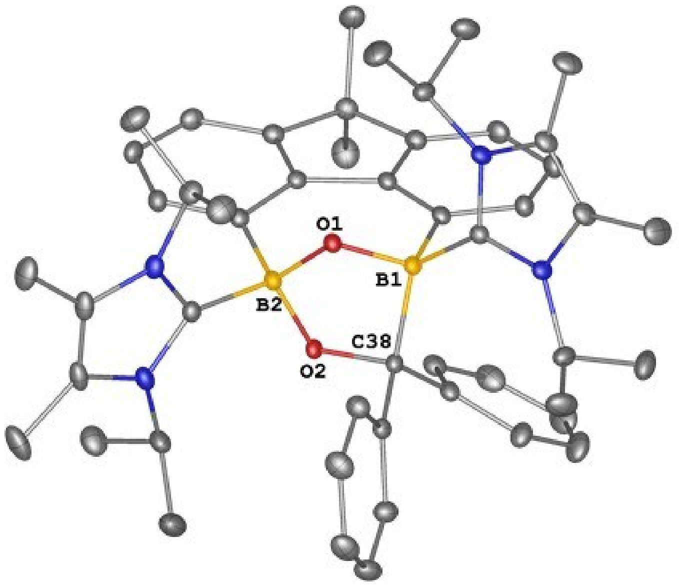

Figure S54. Molecular structure of compound **11**. Thermal ellipsoids are drawn at the 50% probability level. H atoms and solvent CH_2Cl_2 are omitted for clarity.

Table S22. Selected interatomic distances and angles of compound **11.**

Bond lengths [Å]		Angles [°]	
O(1)-B(1)	1.4607(18)	B(2)-O(1)-B(1)	107.38(11)
O(1)-B(2)	1.4496(18)	C(38)-O(2)-B(2)	111.21(10)
O(2)-C(38)	1.4566(16)	O(2)-C(38)-B(1)	96.67(10)
O(2)-B(2)	1.4869(18)	O(1)-B(1)-C(38)	100.39(11)
C(38)-B(1)	1.715(2)	O(1)-B(2)-O(2)	106.93(11)

Table S23. Crystal data and structure refinement for **12**.

Empirical formula	C38 H52 B2 N4 O3	
Formula weight	634.45	
Temperature	149.98(10) K	
Wavelength	1.54184 Å	
Crystal system	Monoclinic	
Space group	P 1 21/c 1	
Unit cell dimensions	a = 22.3826(10) Å	a= 90°.
	b = 10.1666(2) Å	b= 111.796(5)°.
	c = 20.2642(9) Å	g = 90°.
Volume	4281.6(3) $Å^3$	
Z	4	
Density (calculated)	0.984 Mg/m^3	
Absorption coefficient	0.480 mm^{-1}	
F(000)	1368	
Crystal size	0.05 x 0.02 x 0.01 mm^3	
Theta range for data collection	4.255 to 72.719°.	
Index ranges	-26<=h<=27, -10<=k<=12, -25<=l<=23	
Reflections collected	31725	
Independent reflections	8388 [R(int) = 0.0624]	
Completeness to theta = 67.684°	100.0 %	
Absorption correction	Semi-empirical from equivalents	
Max. and min. transmission	1.00000 and 0.44449	
Refinement method	Full-matrix least-squares on F^2	
Data / restraints / parameters	8388 / 6 / 438	
Goodness-of-fit on F^2	1.010	
Final R indices [I>2sigma(I)]	R1 = 0.0547, wR2 = 0.1366	
R indices (all data)	R1 = 0.0885, wR2 = 0.1533	
Extinction coefficient	n/a	
Largest diff. peak and hole	0.724 and -0.265 e.$Å^{-3}$	

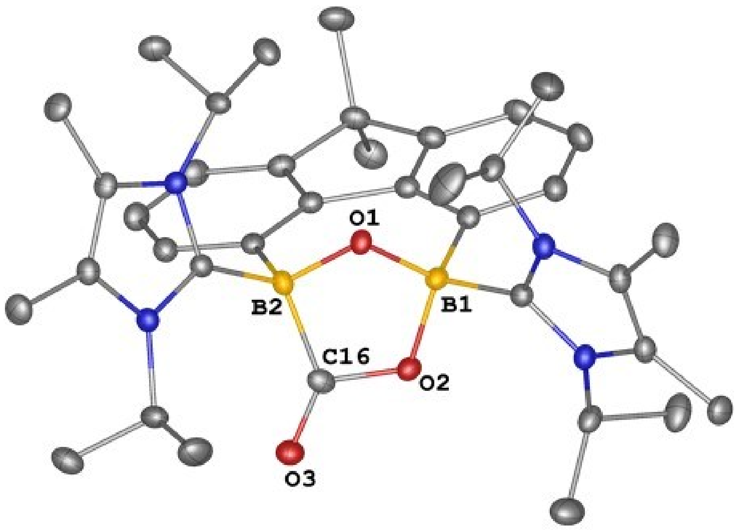

Figure S55. Molecular structure of compound **12**. Thermal ellipsoids are drawn at the 50% probability level. H atoms are omitted for clarity.

Table S24. Selected interatomic distances and angles of compound **12.**

Bond lengths [Å]		Angles [°]	
O(1)-B(1)	1.444(2)	B(1)-O(1)-B(2)	107.90(15)
O(1)-B(2)	1.461(3)	C(16)-O(2)-B(1)	110.62(15)
O(2)-C(16)	1.364(3)	O(2)-C(16)-B(2)	104.78(17)
O(2)-B(1)	1.521(3)	O(1)-B(1)-O(2)	105.66(17)
O(3)-C(16)	1.216(3)	O(1)-B(2)-C(16)	100.82(16)
C(16)-B(2)	1.650(3)		

D. Theoretical Calculations

Computational details

The geometry optimizations followed by the harmonic vibrational frequency calculations of the studied complexes were carried out at the BP86-D3(BJ)/def2TZVPP level.[3] For the sake of reducing the computational cost, the steps involving transition states, the basis set is reduced to def2SVP. Gaussian 16 program[4] was used for these calculations. Natural charges were computed using NBO7 program.[5] The quantum theory of atoms-in-molecules (QTAIM)[6] study was performed at the BP86-D3(BJ)/def2TZVPP level using Multiwfn program.[7]

The energy decomposition analysis (EDA)[8] together with the natural orbitals for chemical valence (NOCV)[9] method were carried out by using the ADF 2019.303 program package.[10] The EDA-NOCV calculations were carried out at the BP86-D3(BJ)/TZ2P-ZORA//BP86-D3(BJ)/def2TZVPP level. This calculation divides the intrinsic interaction energy (ΔE_{int}) between two fragments into three energy components as follows:

$$\Delta E_{\mathrm{int}} = \Delta E_{\mathrm{elstat}} + \Delta E_{\mathrm{Pauli}} + \Delta E_{\mathrm{orb}} + \Delta E_{\mathrm{disp}} \qquad (1)$$

While the electrostatic $\Delta E_{\mathrm{elstat}}$ term accounts for the quasiclassical electrostatic interaction between the unperturbed charge distributions of the prepared fragments, the Pauli repulsion $\Delta E_{\mathrm{Pauli}}$ represents the energy change associated with the transformation from the superposition of the unperturbed electron densities of the isolated fragments to the wavefunction, that properly obeys the Pauli principle through explicit antisymmetrization and renormalization of the production wavefunction. Since we used D3(BJ), it provides us with the dispersion interactions between the fragments. Finally, the mixing of orbitals, charge transfer and polarization between the isolated fragments provide the orbital term ΔE_{orb}, which can be further decomposed into contributions from each irreducible representation of the point group of the interacting system as follows:

$$\Delta E_{orb} = \sum_{r} \Delta E_{r} \qquad (2)$$

The combination of the EDA with NOCV makes the partition of the total ΔE_{orb} into pairwise contributions of the orbital interactions which give very important information about the bonding situation. The charge deformation $\Delta\rho_k(r)$, resulting from the mixing of the orbital pairs $\psi_{\mathrm{k}}(\mathrm{r})$ and $\psi_{\mathrm{-k}}(\mathrm{r})$ of the interacting fragments provides the amount and the shape of the charge flow due to the orbital interactions (see Equation 3), and the associated energy term ΔE_{orb} shows the size of stabilizing orbital energy originated from such interaction (Equation 4).

$$\Delta\rho_{orb}(r) = \sum_{k} \Delta\rho_{k}(r) = \sum_{k=1}^{N/2} \nu_{k}[-\psi_{-k}^{2}(r) + \psi_{k}^{2}(r)] \qquad (3)$$

$$\Delta E_{orb} = \sum_{k} \Delta E_{k}^{orb} = \sum_{k=1}^{N/2} \nu_{k}[-F_{-k,-k}^{TS} + F_{k,k}^{TS}] \qquad (4)$$

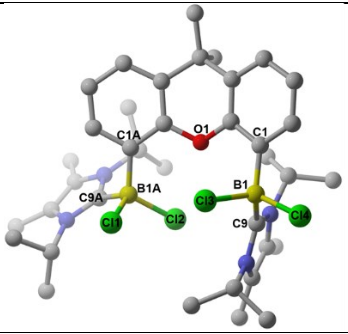

B1A-Cl1=1.887
B1A-Cl2=1.901
B1A-C9A=1.653
B1A-C1A=1.619
B1-Cl3=1.895
B1-Cl4=1.925
B1-C9=1.630
B1-C1=1.618
∠C1A-B1A-C9A=107.0°
∠C1-B1-C9=119.4°
∠C1-O1-C1A=153.8°

3

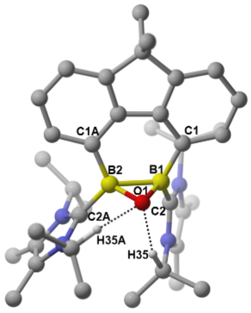

O1-B1=1.466 (1.566)
O1-B2=1.466 (1.663)
B1-C2=1.563 (1.595)
B2-C2A=1.587 (1.614)
B1-C1=1.586 (1.560)
B2-C1A=1.573 (1.570)
B1-B2=1.812 (1.725)
O1-H35=2.009 (2.450)
O1-H35A=2.081 (1.935)
∠B1-O1-B2=76.3°(64.5°)

4

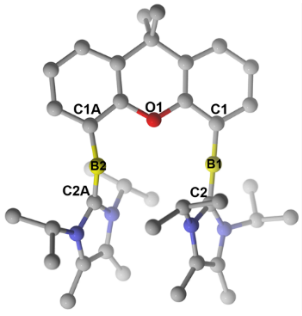

C1A-B2= 1.494
C1-B1=1.506
B2-C2A= 1.455
B1-C2=1.487
∠C1A-B2-C2A=178.1°
∠C1-B1-C2=143.5°

4’

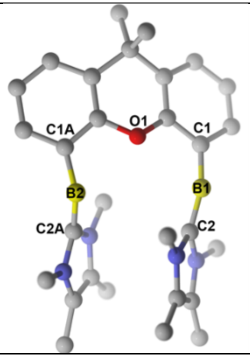

C1A-B2 = 1.497
C1-B1 = 1.521
B2-C2A = 1.471
B1-C2 = 1.450
∠C1A-B2-C2A=156.9°
∠C1-B1-C2=133.2°

6’

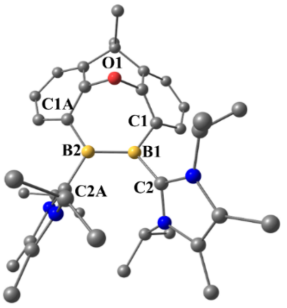

B1-B2 = 1.660
B1-C1 = 1.599
B1-C2 = 1.566
B2-C1A = 1.595
B2-C2A = 1.573
<C1A-B2-C2A = 118.1
<C1-B1-C2 = 114.4

4’’

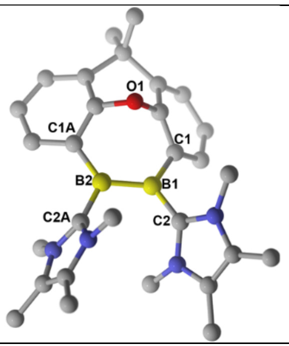

B1-B2 = 1.664
C1A-B2 = 1.598
C1-B1 = 1.593
B2-C2A = 1.565
B1-C2= 1.553
∠C1A-B2-C2A=120.2°
∠C1-B1-C2=116.9°

6’’

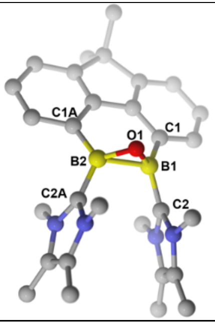

C1A-B2/ C1-B1= 1.577
B1-C2/B2-C2A = 1.564
B1-O1/ B2-O1=1.460
B1-B2=1.858
∠B1-O1-B2=79.1°
∠C1A-B2-C2A/∠C1-B1-C2=121.2°

6(B-O-B)

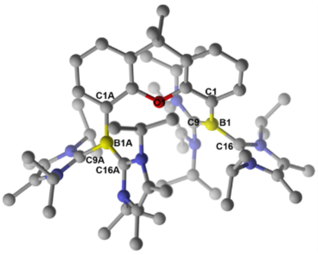

C1A-B1A /C1-B1=1.579
B1A-C9A/ B1-C9= 1.558
B1A-C16A/B1-C16= 1.519
∠C1A-B1A-C9A/∠C1-B1-C9= 114.1°
∠C1A-B1A-C16A/∠C1-B1-C16= 127.6°

4(4-IPr)

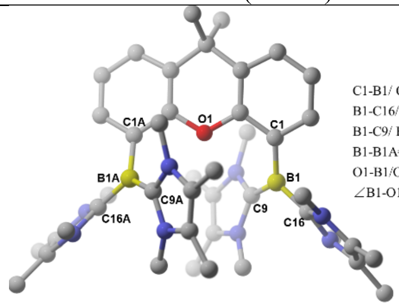

C1-B1/ C1A-B1 A=1.569 (1.577)
B1-C16/ B1A-C16A =1.518 (1.511)
B1-C9/ B1A-C9A =1.525 (1.533)
B1-B1A=4.880 (4.873)
O1-B1/O1-B1A=2.903 (2.941)
∠B1-O1-B1A=114.4°(111.9°)

6

Figure S56. The minimum energy geometries at the BP86-D3(BJ)/def2-TZVPP level. Selected bond lengths in Å, experimental values are given in parentheses. Hydrogen atoms are omitted for clarity (color code, C: gray, H: white, O: red, N: blue, B: yellow).

Table S25. Calculated Wiberg bond order (P) and natural charge (q) for **4** at the BP86-D3(BJ)/def2-TZVPP Level with the NBO 7.0 Program.

		**4**	
	**q**		**P**
B1	0.371	B1-B2	0.72
B2	0.364	B1-O1	0.87
O1	-0.814	B2-O1	0.87
C1	-0.253	B1-C1	0.96
C1A	-0.257	B1-C2	1.00
C2	0.207	B2-C1A	0.97
C2A	0.272	B2-C2A	0.94
H35	0.285	O1-H35	0.156
H35A	0.283	O1-H35A	0.096
IPr	0.319		
IPr	0.499		

Table S26. Calculated Wiberg bond order (P) and natural charge (q) for **6** at the BP86-D3(BJ)/def2-TZVPP Level with the NBO 7.0 Program.

	**6**		
	**q**		**P**
B1	0.022	B1-C16	1.18
B1A	0.022	B1-C9	1.15
O1	-0.436	B1-C1	0.99
C16	0.156	BA1-C9A	1.15
C9	0.188	BA1-C16A	1.18
C9A	0.188	BA1-C1A	0.99
C16A	0.156		
C1	-0.277		
C1A	-0.277		
IMe1	0.162		
IMe2	0.249		
IMe3	0.162		
IMe4	0.249		

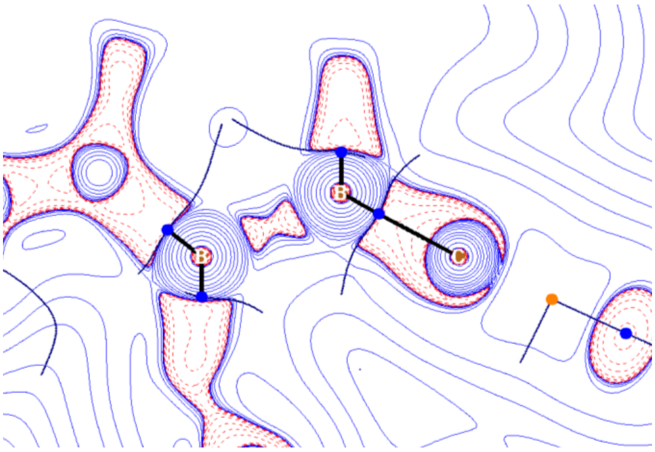

Figure S57. The contour plot of Laplacian of electron density for complex **4** in the plane **B1-B2-C2(IPr)** at the BP86-D3(BJ)/def2-TZVPP level.

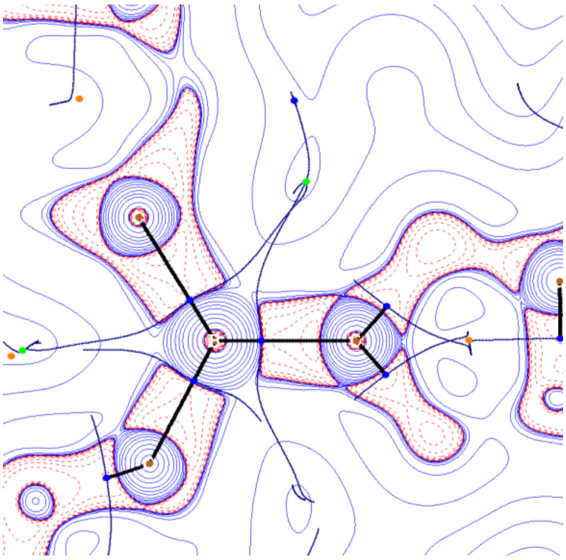

Figure S58. The contour plot of Laplacian of electron density for complex **6** in the plane **C9(IMe)-B-C1(Ph)** at the BP86-D3(BJ)/def2-TZVPP level.

Table S27. The EDA-NOCV results of complexes **4** and **6** at the BP86-D3(BJ)/TZ2P//BP86-D3(BJ)/def2-TZVPP Level. Energy Values are Given in kcal/mol.

Energy	Interaction	**4**	Interaction	**6**
ΔE_{int}		-176.9		-599.7
ΔE_{Pauli}		548.1		1144.5
ΔE_{elstat}[a]		-343.1 (47.2%)		-684.1 (39.2%)
ΔE_{disp}[a]		-25.8 (3.6%)		-37.3 (2.2%)
ΔE_{orb}[a]		-357.8 (49.2%)		-1022.5 (58.6%)
$\Delta E_{orb(1)}$[b]	$(IPr)_2 \rightarrow$diboraoxirane (+,+) σ-donation/polarization	-165.5 (46.3%)	$(IMe)_4 \rightarrow$bis(borylene) σ-donation	-318.8 (31.2%)
$\Delta E_{orb(2)}$[b]	$(IPr)_2 \rightarrow$diboraoxirane (+,-) σ-donation	-101.5 (28.4%)	$(IMe)_4 \leftarrow$bis(borylene) π-backdonation	-204.2 (20.0%)
$\Delta E_{orb(3)}$[b]	$(IPr)_2 \rightarrow$diboraoxirane (+,+) σ-donation	-38.1 (10.6%)	$(IMe)_4 \rightarrow$bis(borylene) σ-donation	-121.1 (11.9%)
$\Delta E_{orb(4)}$[b]			$(IMe)_4 \rightarrow$bis(borylene) σ-donation	-106.3 (10.4%)
$\Delta E_{orb(5)}$[b]			$(IMe)_4 \leftarrow$bis(borylene) π-backdonation	-94.5 (9.3%)
$\Delta E_{orb(6)}$[b]			$(IMe)_4 \rightarrow$bis(borylene) σ-donation	-75.9 (7.4%)
$\Delta E_{orb(7)}$[b]			$(IMe)_4 \rightarrow$bis(borylene) σ-donation	-31.0 (3.0%)
$\Delta E_{orb(rest)}$[b]		-52.7 (14.7%)		-70.7 (6.9%)

[a]The percentage contribution with respect to total attraction is given in parentheses; [b]The percentage contribution in parentheses is given with respect to total orbital interaction.

	Deformation density	Diboraoxirane		$(IPr)_2$
$\Delta\rho_{(1)}$	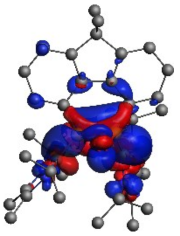<br>$\Delta E_{orb(1)}$ = -165.5kcal/mol<br>$\lvert\nu_1\rvert$ = 1.14	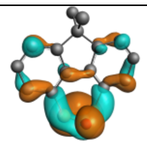<br>HOMO<br>ν = -0.46<br>↓<br>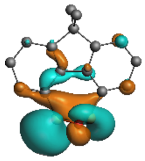<br>LUMO<br>ν = 0.51<br>+<br>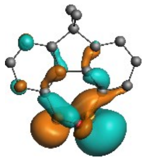<br>LUMO+1<br>ν = 0.31	←	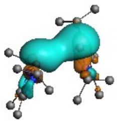<br>HOMO-1<br>ν =-0.51
$\Delta\rho_{(2)}$	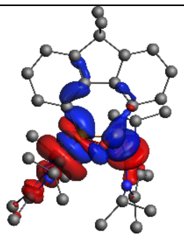<br>$\Delta E_{orb(2)}$ = -101.5 kcal/mol<br>$\lvert\nu_2\rvert$ = 0.71	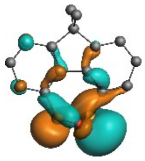<br>LUMO+1<br>ν = 0.39<br>+<br>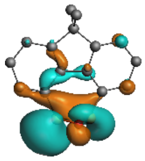<br>LUMO<br>ν = 0.29	←	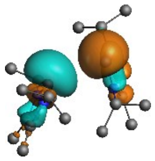<br>HOMO<br>ν = -0.63

$\Delta\rho_{(3)}$	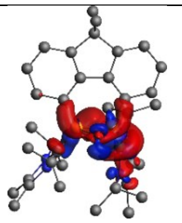<br>$\Delta E_{orb(3)}$ = -38.1 kcal/mol<br>$\lvert\nu_3\rvert$ = 0.54	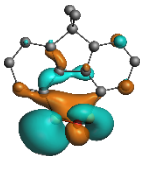<br>LUMO+1<br>ν = 0.07	←	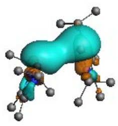<br>HOMO-1<br>ν = -0.21

Figure S59. Plot of the deformation densities, $\Delta\rho_{(1)\text{-}(3)}$ shown as the sum of α and β electronic charge corresponding to $\Delta E_{orb(1)\text{-}(3)}$ and the related interacting orbitals of the fragments in the complex **4** at the BP86-D3(BJ)/TZ2P//BP86-D3(BJ)/def2-TZVPP level. The direction of the charge flow of the deformation densities is red→blue. The isovalue for $\Delta\rho_{(1)\text{-}(2)}$ is 0.001 au. The isovalue for $\Delta\rho_{(3)}$ is 0.0005 au.

	Deformation density	bis(borylene)		$(IMe)_4$
$\Delta\rho_{(1)}$	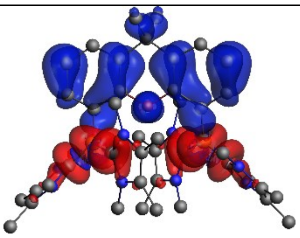<br>$\Delta E_{orb(2)}$ = -318.8 kcal/mol<br>$\lvert\nu_1\rvert$ = 1.42	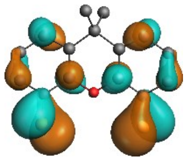<br>LUMO<br>ν=1.64	←	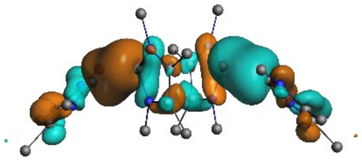<br>HOMO-2<br>ν=-0.52
$\Delta\rho_{(2)}$	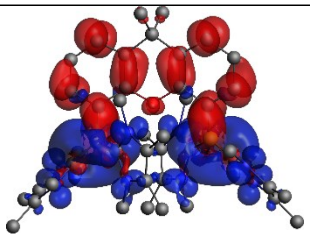<br>$\Delta E_{orb(1)}$ = -204.2 kcal/mol<br>$\lvert\nu_2\rvert$ = 1.60	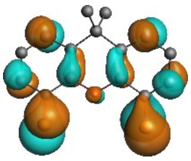<br>HOMO<br>ν= -1.2	→	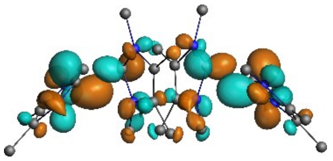<br>LUMO<br>ν=0.58
$\Delta\rho_{(3)}$	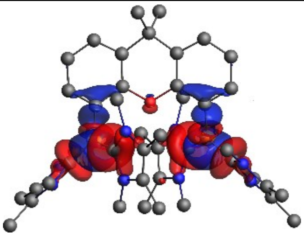<br>$\Delta E_{orb(4)}$ = -121.1 kcal/mol<br>$\lvert\nu_3\rvert$ = 0.74	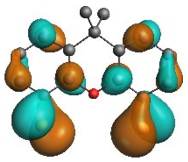<br>LUMO<br>ν=0.90	←	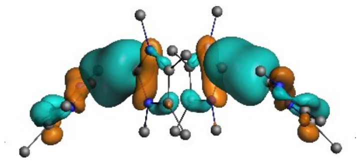<br>HOMO-3<br>ν=-0.68

$\Delta\rho_{(4)}$	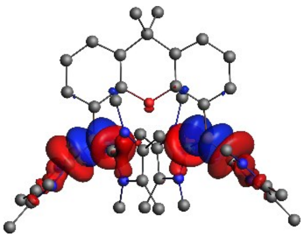<br>$\Delta E_{orb(5)}$ = -106.3 kcal/mol<br>$\lvert\nu_4\rvert$ = 0.66	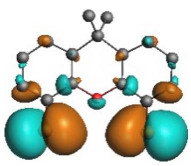<br>LUMO<br>ν=0.52	←	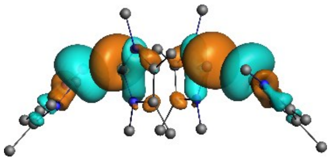<br>HOMO<br>ν=-0.66
$\Delta\rho_{(5)}$	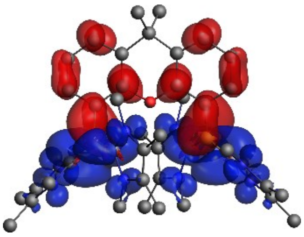<br>$\Delta E_{orb(3)}$ = -94.5 kcal/mol<br>$\lvert\nu_1\rvert$ = 1.26	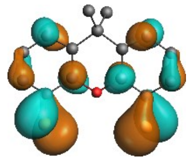<br>HOMO<br>ν=-1.04	→	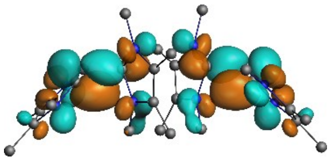<br>LUMO+1<br>ν=0.86
$\Delta\rho_{(6)}$	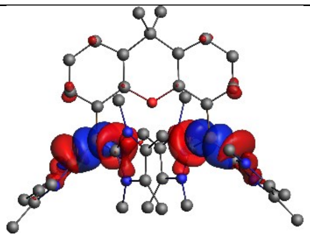<br>$\Delta E_{orb(6)}$ = -75.9 kcal/mol<br>$\lvert\nu_1\rvert$ = 0.58	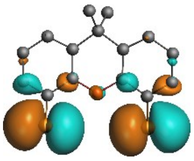<br>LUMO<br>ν=0.18	←	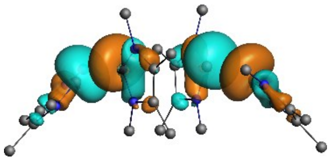<br>HOMO<br>ν=-0.40
$\Delta\rho_{(7)}$	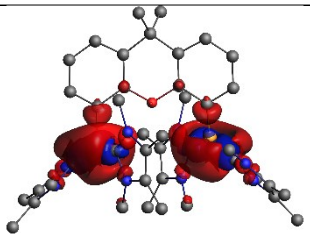<br>$\Delta E_{orb(6)}$ = -31.0 kcal/mol<br>$\lvert\nu_1\rvert$ = 0.34	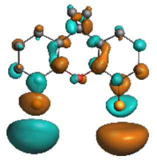<br>LUMO+8<br>ν=0.02	←	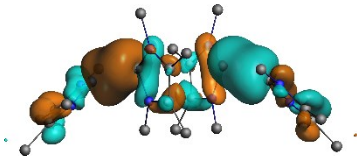<br>HOMO-2<br>ν=-0.04

Figure S60. Plot of the deformation densities, $\Delta\rho_{(1)\text{-}(7)}$ shown as the sum of α and β electronic charge corresponding to $\Delta E_{orb(1)\text{-}(7)}$ and the related interacting orbitals of the fragments in the complex **6** at the BP86-D3(BJ)/TZ2P//BP86-D3(BJ)/def2-TZVPP level. The direction of the charge flow of the deformation densities is red→blue. The isovalue for $\Delta\rho_{(1)\text{-}(2)}$ is 0.001 au. The isovalue for $\Delta\rho_{(3)\text{-}(7)}$ is 0.0005 au.

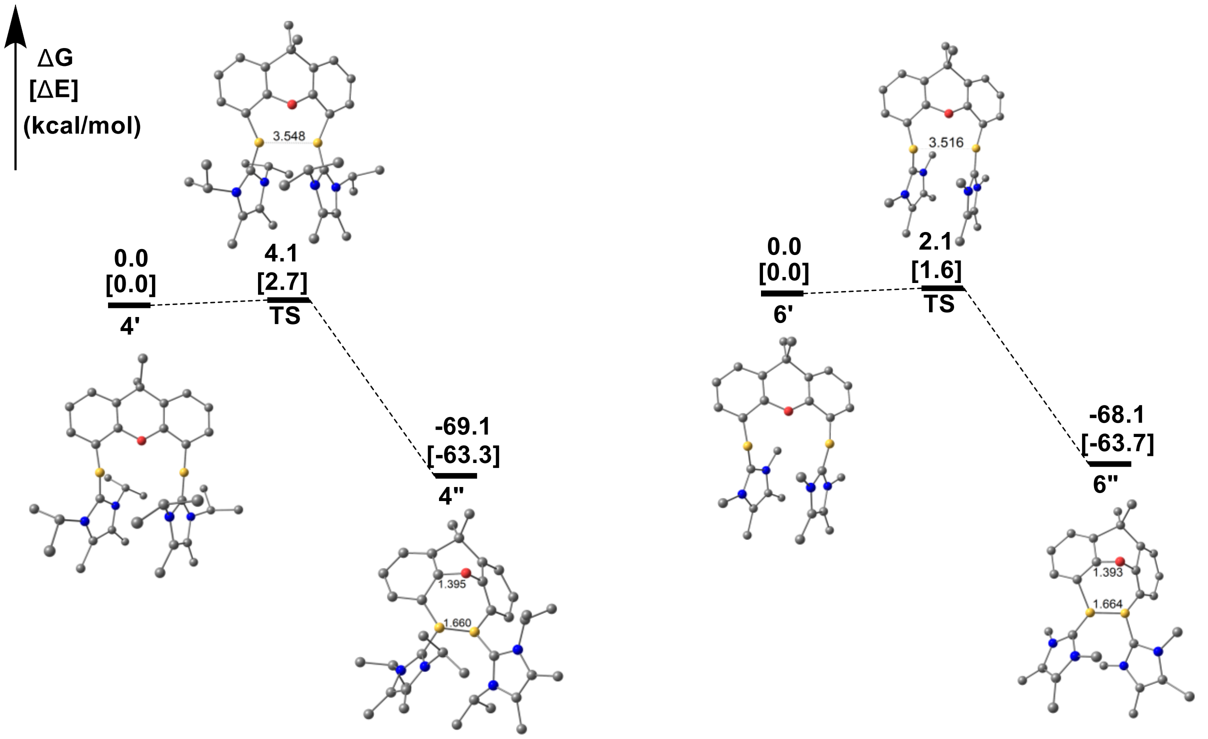

Figure S61. The activation energy barrier for **4′→4″** and **6′→6″** conversion at the BP86-D3(BJ)/def2-SVP level. Key bond distances are given in Å. Hydrogen atoms have been omitted for clarity.

Table S28. The Cartesian coordinates of complexes at the BP86-D3(BJ)/def2-TZVPP level.

**3**			
E = -3627.3299866 au			
0 1			
Cl	0.659523000	-1.154265000	0.605001000
Cl	-2.128128000	-0.726643000	-2.464429000
Cl	1.484998000	-1.203288000	-2.353807000
Cl	-4.890658000	-1.401794000	-1.190654000
O	-0.983320000	1.304605000	-0.337907000
N	-2.150922000	-2.793957000	0.183064000
N	4.326670000	-0.943367000	-0.389157000
N	3.523355000	0.550102000	0.970999000
N	-2.429261000	-1.191008000	1.622342000
C	3.186988000	-0.267521000	-0.072919000
C	0.123828000	2.003319000	-0.787723000
C	1.306975000	1.286199000	-1.055827000
C	-3.340319000	1.016677000	-0.661867000
C	-2.565984000	-1.500626000	0.301625000
C	0.052230000	3.406391000	-0.926913000
C	4.402806000	-2.006233000	-1.424495000
H	3.355112000	-2.201943000	-1.671394000
C	-2.239003000	1.889815000	-0.507830000
C	4.876853000	0.418061000	1.279441000
C	2.247018000	3.375185000	-1.957861000
H	3.062215000	3.894532000	-2.463538000
C	5.387886000	-0.520967000	0.419332000
C	-1.705288000	-3.277147000	1.410824000
C	2.344971000	2.017076000	-1.668004000
H	3.263676000	1.487411000	-1.932376000
C	-1.886412000	-2.268591000	2.323117000
C	2.533949000	1.345869000	1.745722000
H	1.600728000	1.218327000	1.192643000
C	-2.278611000	-3.598137000	-1.059883000

H	-2.661040000	-2.888203000	-1.797395000
C	1.111453000	4.074282000	-1.546553000
H	1.057953000	5.151453000	-1.698508000
C	-2.367412000	3.285894000	-0.490714000
C	-4.601838000	1.628034000	-0.754707000
H	-5.472328000	0.984374000	-0.875873000
C	-2.897678000	0.071262000	2.232522000
H	-3.194776000	0.683583000	1.377796000
C	4.997578000	-3.305925000	-0.879456000
H	6.089045000	-3.277811000	-0.775167000
H	4.755771000	-4.114906000	-1.581989000
H	4.554166000	-3.562754000	0.092067000
C	-1.119618000	4.132482000	-0.274912000
C	5.608019000	1.202113000	2.316010000
H	5.719732000	2.257137000	2.027434000
H	6.612375000	0.783922000	2.451069000
H	5.109516000	1.176722000	3.292739000
C	2.314730000	0.739129000	3.132856000
H	2.055083000	-0.322869000	3.040249000
H	1.472750000	1.255822000	3.613527000
H	3.187607000	0.842699000	3.790706000
C	-0.923173000	-4.084926000	-1.568660000
H	-0.209172000	-3.252792000	-1.608605000
H	-1.052163000	-4.468958000	-2.589994000
H	-0.502031000	-4.894734000	-0.958644000
C	-4.763866000	3.013689000	-0.732054000
H	-5.760215000	3.449643000	-0.820415000
C	5.077128000	-1.491396000	-2.695108000
H	4.542318000	-0.612072000	-3.076396000
H	5.035133000	-2.270166000	-3.468684000
H	6.132358000	-1.227289000	-2.539177000
C	-3.648596000	3.838454000	-0.614321000
H	-3.775392000	4.920478000	-0.610628000
C	-3.333085000	-4.693466000	-0.890607000

H	-3.031106000	-5.482254000	-0.188296000
H	-3.507738000	-5.166820000	-1.866641000
H	-4.280394000	-4.254933000	-0.552234000
C	6.794452000	-1.009887000	0.326982000
H	6.928978000	-1.994775000	0.796990000
H	7.459665000	-0.304347000	0.839221000
H	7.138611000	-1.086554000	-0.711733000
C	-1.032365000	-4.588488000	1.627486000
H	-1.560297000	-5.422935000	1.152088000
H	-0.973959000	-4.800029000	2.702292000
H	-0.004979000	-4.570965000	1.233751000
C	2.854340000	2.838553000	1.752691000
H	3.721915000	3.088319000	2.376011000
H	1.987265000	3.375490000	2.158993000
H	3.020338000	3.200161000	0.730059000
C	-1.483428000	-2.247946000	3.757335000
H	-0.562871000	-1.664093000	3.909044000
H	-1.285173000	-3.272070000	4.096352000
H	-2.258360000	-1.828529000	4.410787000
B	1.671071000	-0.257750000	-0.731521000
C	-0.834391000	4.206339000	1.251265000
H	-0.682044000	3.201775000	1.666538000
H	-1.683341000	4.670994000	1.774026000
H	0.070084000	4.803654000	1.440313000
B	-3.177269000	-0.576666000	-0.893806000
C	-1.778122000	0.819057000	2.957220000
H	-1.536135000	0.387979000	3.936911000
H	-2.096624000	1.857596000	3.118631000
H	-0.874129000	0.825921000	2.334750000
C	-4.157535000	-0.161256000	3.068251000
H	-4.928182000	-0.653060000	2.460125000
H	-4.552298000	0.808144000	3.402343000
H	-3.971003000	-0.774476000	3.960596000
C	-1.289524000	5.562804000	-0.803056000

H	-0.383428000	6.154778000	-0.617576000
H	-2.109967000	6.072820000	-0.282085000
H	-1.501849000	5.572257000	-1.880698000

4

E = -1784.23.1429 au

0 1

N	-1.884282000	1.899396000	-1.004873000
N	-0.353934000	2.847057000	0.258803000
N	-2.029604000	-1.124892000	1.320584000
N	-2.125616000	-2.319050000	-0.505253000
C	-0.553846000	1.799584000	-0.635949000
C	-2.459952000	1.040761000	-2.059011000
H	-1.713198000	0.221974000	-2.149440000
C	-3.803747000	0.426448000	-1.645516000
H	-3.753160000	0.007713000	-0.620598000
H	-4.052045000	-0.406735000	-2.332977000
H	-4.646115000	1.143791000	-1.692907000
C	-2.512466000	1.777633000	-3.403648000
H	-3.218011000	2.635287000	-3.389925000
H	-2.840100000	1.082987000	-4.204457000
H	-1.506313000	2.156636000	-3.672346000
C	-2.497281000	2.992433000	-0.369106000
C	-3.927239000	3.392849000	-0.537712000
H	-4.222645000	3.465023000	-1.604469000
H	-4.091918000	4.388222000	-0.082150000
H	-4.631651000	2.685796000	-0.048341000
C	-1.537515000	3.581734000	0.427552000
C	-1.695862000	4.755456000	1.338669000
H	-1.661373000	4.470468000	2.412371000
H	-2.675936000	5.238100000	1.161351000
H	-0.913114000	5.523256000	1.178413000
C	0.925266000	3.053896000	0.970694000
H	1.567727000	2.241207000	0.582150000

C	0.807398000	2.844762000	2.485200000
H	1.818332000	2.901793000	2.936719000
H	0.401635000	1.840711000	2.712431000
H	0.177010000	3.607825000	2.984710000
C	1.599574000	4.380116000	0.597225000
H	1.114089000	5.257107000	1.072639000
H	1.598567000	4.524477000	-0.499780000
H	2.657109000	4.360695000	0.928664000
C	2.051129000	1.032419000	-1.147956000
C	2.659455000	2.166897000	-1.748478000
H	2.017871000	2.955793000	-2.178815000
C	4.057607000	2.310130000	-1.833850000
H	4.483179000	3.201695000	-2.323743000
C	4.929385000	1.328465000	-1.301743000
H	6.021825000	1.468324000	-1.356504000
C	4.366662000	0.196616000	-0.704222000
C	2.956931000	0.057531000	-0.660546000
C	5.035405000	-0.989034000	0.012171000
C	5.917957000	-0.508028000	1.183891000
H	6.761558000	0.113033000	0.815251000
H	6.345492000	-1.370605000	1.737467000
H	5.325833000	0.100783000	1.896882000
C	5.864467000	-1.839954000	-0.977127000
H	5.232255000	-2.184013000	-1.820182000
H	6.285530000	-2.734483000	-0.471126000
H	6.707626000	-1.249420000	-1.394070000
C	2.620661000	-1.159356000	0.063045000
C	3.811906000	-1.774331000	0.516250000
C	3.740261000	-2.908409000	1.334040000
H	4.648323000	-3.410256000	1.706665000
C	2.458529000	-3.392240000	1.692501000
H	2.378920000	-4.279858000	2.342266000
C	1.285000000	-2.774035000	1.218938000
H	0.310334000	-3.209311000	1.506778000

C	1.317325000	-1.639907000	0.357893000
C	-1.354065000	-1.408956000	0.167856000
C	-1.602732000	-2.997002000	-1.722512000
H	-0.780618000	-2.327918000	-2.045527000
C	-2.616766000	-3.052596000	-2.869262000
H	-3.431429000	-3.784885000	-2.704827000
H	-2.085248000	-3.354170000	-3.793848000
H	-3.065500000	-2.057233000	-3.055509000
C	-1.014803000	-4.365071000	-1.357820000
H	-0.227936000	-4.254690000	-0.585833000
H	-0.548170000	-4.820130000	-2.254766000
H	-1.788536000	-5.068710000	-0.983904000
C	-3.279150000	-2.618243000	0.229045000
C	-4.352176000	-3.561783000	-0.207155000
H	-4.943300000	-3.159846000	-1.056813000
H	-5.054607000	-3.744169000	0.628593000
H	-3.943507000	-4.543611000	-0.520588000
C	-3.220072000	-1.860894000	1.388531000
C	-4.210487000	-1.784760000	2.504968000
H	-3.721316000	-1.845516000	3.497610000
H	-4.924208000	-2.627879000	2.433457000
H	-4.804073000	-0.845969000	2.483332000
C	-1.480062000	-0.154962000	2.291462000
H	-0.630377000	0.277775000	1.714145000
C	-0.906491000	-0.852820000	3.528623000
H	-1.693577000	-1.338673000	4.142569000
H	-0.395514000	-0.106712000	4.170879000
H	-0.158212000	-1.613505000	3.232164000
C	-2.470969000	0.967382000	2.611147000
H	-2.881046000	1.407067000	1.680915000
H	-1.938461000	1.775128000	3.150745000
H	-3.306980000	0.635369000	3.257996000
B	0.498281000	0.705730000	-1.032351000
B	0.082410000	-0.914538000	-0.305063000

O	0.098771000	-0.524957000	-1.718749000

6

E = -2238.7137770 au

0 1

O	-0.000034000	1.607730000	-0.000228000
N	-4.174102000	-1.083515000	-0.716937000
N	-4.062525000	-1.836020000	1.342900000
N	-0.522140000	0.388252000	2.643517000
N	-0.774080000	-1.680471000	1.986471000
C	-1.198212000	2.295040000	-0.018065000
C	-5.174085000	-2.049901000	-0.578380000
C	-3.456983000	-0.928436000	0.467152000
C	-5.108537000	-2.521545000	0.703298000
C	-1.243018000	3.686695000	-0.178479000
C	-1.265115000	-0.393962000	1.769702000
C	-3.586172000	2.150112000	-0.075015000
C	-2.359231000	1.491283000	0.170807000
C	-3.888121000	-1.760788000	2.778052000
C	0.416363000	-0.375564000	3.336845000
C	-2.499014000	4.281033000	-0.392568000
C	-3.661167000	3.506791000	-0.392614000
C	-0.000003000	4.556938000	-0.000390000
C	-5.925319000	-3.557409000	1.391143000
C	-3.756479000	-0.473588000	-1.957345000
C	-6.072638000	-2.435775000	-1.700223000
C	-0.814801000	1.773137000	2.946552000
C	0.262426000	-1.671872000	2.929668000
C	-1.151396000	-2.817455000	1.183743000
C	1.353233000	0.226972000	4.323056000
B	-2.320091000	0.035445000	0.755084000
C	0.208396000	5.459518000	-1.242439000
C	1.025072000	-2.893566000	3.298369000
H	-4.511147000	1.584199000	0.063387000

H	-4.630673000	3.977203000	-0.569172000
H	-2.566950000	5.360730000	-0.534945000
H	-3.060691000	-2.390546000	3.142753000
H	-3.641245000	-0.716505000	3.029338000
H	-4.811851000	-2.067264000	3.284377000
H	-5.296827000	-4.363278000	1.804746000
H	-6.515813000	-3.146401000	2.226590000
H	-6.632450000	-4.012129000	0.686217000
H	-4.075999000	-1.093029000	-2.804473000
H	-4.158030000	0.545674000	-2.075056000
H	-2.658483000	-0.397268000	-1.938327000
H	-6.636169000	-1.571719000	-2.086222000
H	-5.519325000	-2.868960000	-2.549959000
H	-6.798808000	-3.185925000	-1.364126000
H	-0.151730000	2.460337000	2.400775000
H	-1.850071000	1.978811000	2.650079000
H	-0.699550000	1.947776000	4.024515000
H	-2.075734000	-3.300549000	1.539086000
H	-1.346651000	-2.452917000	0.162066000
H	-0.343567000	-3.556367000	1.183643000
H	1.950483000	1.032831000	3.868647000
H	0.825336000	0.659902000	5.188179000
H	2.044823000	-0.533296000	4.705976000
H	0.374634000	4.846881000	-2.139020000
H	-0.671346000	6.094479000	-1.418239000
H	1.082297000	6.111984000	-1.108417000
H	0.369937000	-3.695788000	3.674686000
H	1.590446000	-3.303672000	2.444089000
H	1.750903000	-2.658121000	4.086449000
N	4.174240000	-1.083265000	0.716984000
N	4.062581000	-1.836251000	-1.342662000
N	0.522058000	0.387468000	-2.643675000
N	0.774098000	-1.681038000	-1.985983000
C	1.198158000	2.295019000	0.017555000

C	5.174185000	-2.049714000	0.578629000
C	3.457034000	-0.928513000	-0.467102000
C	5.108577000	-2.521664000	-0.702937000
C	1.242996000	3.686692000	0.177794000
C	1.265056000	-0.394426000	-1.769601000
C	3.586115000	2.150034000	0.074735000
C	2.359172000	1.491210000	-0.171138000
C	3.887931000	-1.761549000	-2.777790000
C	-0.416418000	-0.376613000	-3.336759000
C	2.498987000	4.281031000	0.391892000
C	3.661123000	3.506753000	0.392142000
C	5.925498000	-3.557510000	-1.390637000
C	3.756542000	-0.473162000	1.957285000
C	6.072896000	-2.435183000	1.700484000
C	0.814536000	1.772326000	-2.947036000
C	-0.262394000	-1.672792000	-2.929203000
C	1.151354000	-2.817724000	-1.182782000
C	-1.353303000	0.225555000	-4.323185000
B	2.320088000	0.035273000	-0.755164000
C	-0.208394000	5.459621000	1.241588000
C	-1.024937000	-2.894644000	-3.297576000
H	4.511085000	1.584059000	-0.063457000
H	4.630628000	3.977152000	0.568738000
H	2.566925000	5.360748000	0.534128000
H	3.060487000	-2.391490000	-3.142159000
H	3.640938000	-0.717370000	-3.029411000
H	4.811595000	-2.068128000	-3.284176000
H	5.297159000	-4.363582000	-1.804074000
H	6.515870000	-3.146521000	-2.226180000
H	6.632745000	-4.011966000	-0.685658000
H	4.076142000	-1.092402000	2.804528000
H	4.157978000	0.546166000	2.074800000
H	2.658528000	-0.396970000	1.938250000
H	6.636392000	-1.570948000	2.086138000

H	5.519729000	-2.868143000	2.550429000
H	6.799091000	-3.185383000	1.364549000
H	0.151318000	2.459549000	-2.401460000
H	1.849757000	1.978230000	-2.650564000
H	0.699326000	1.946681000	-4.025049000
H	2.075685000	-3.300980000	-1.537904000
H	1.346534000	-2.452793000	-0.161229000
H	0.343514000	-3.556624000	-1.182462000
H	-1.950647000	1.031494000	-3.869033000
H	-0.825407000	0.658276000	-5.188413000
H	-2.044809000	-0.534886000	-4.705913000
H	-0.374646000	4.847053000	2.138212000
H	0.671361000	6.094577000	1.417343000
H	-1.082286000	6.112089000	1.107508000
H	-0.369755000	-3.696916000	-3.673712000
H	-1.590257000	-3.304574000	-2.443177000
H	-1.750812000	-2.659446000	-4.085690000

Table S29. The Cartesian coordinates of complexes at the BP86-D3(BJ)/def2-SVP level.

4'

E = -1783.9803246 au

0 1

O	2.020562000	-0.171436000	-0.298825000
N	-1.699667000	1.279786000	-1.106092000
N	-1.793474000	2.998622000	0.361966000
C	2.799038000	0.958752000	-0.302588000
C	-3.053931000	1.641306000	-0.939619000
C	-0.894264000	2.158964000	-0.346856000
C	-3.112387000	2.696196000	-0.063028000
C	4.195392000	0.911194000	-0.195281000
C	2.820229000	3.389623000	-0.473787000
C	2.053587000	2.182559000	-0.410149000
C	-1.396694000	4.357467000	0.760641000

C	4.892732000	2.145032000	-0.211805000
C	4.212866000	3.367810000	-0.341202000
C	4.958061000	-0.413500000	-0.056715000
C	-4.291729000	3.380176000	0.544840000
C	-1.059652000	0.599514000	-2.254105000
C	-4.200893000	0.860360000	-1.495235000
B	0.560216000	2.183031000	-0.356497000
C	5.866017000	-0.604186000	-1.301514000
H	2.295327000	4.347958000	-0.608892000
H	4.781591000	4.310603000	-0.368415000
H	5.991104000	2.141728000	-0.140320000
H	-2.245164000	4.756229000	1.356194000
H	-4.277659000	3.319511000	1.657318000
H	-4.346947000	4.459247000	0.279977000
H	-5.231762000	2.912515000	0.194622000
H	-0.107095000	0.202539000	-1.830568000
H	-4.241507000	0.881918000	-2.603627000
H	-4.150393000	-0.204276000	-1.183523000
H	-5.154117000	1.280817000	-1.121211000
H	5.254727000	-0.635084000	-2.225618000
H	6.592417000	0.229426000	-1.394546000
H	6.436728000	-1.552924000	-1.233691000
N	-2.289821000	-2.699245000	-0.035277000
N	-1.524556000	-1.075112000	1.288536000
C	2.600910000	-1.416671000	-0.105294000
C	3.981182000	-1.592382000	0.030908000
C	-1.129105000	-2.020585000	0.343262000
C	2.174667000	-3.787023000	0.175498000
C	1.646840000	-2.481147000	-0.055558000
C	-3.386178000	-2.187963000	0.672356000
C	4.451735000	-2.915104000	0.226096000
C	3.554735000	-3.994854000	0.293290000
C	-2.135544000	-3.830377000	-0.947448000
C	-2.915166000	-1.153647000	1.465713000

C	-0.476510000	-0.455280000	2.137288000
C	-4.781670000	-2.702594000	0.537917000
B	0.196934000	-2.166840000	-0.313620000
C	5.828766000	-0.372198000	1.226288000
C	-3.725675000	-0.216498000	2.300762000
H	1.486693000	-4.645474000	0.235943000
H	3.939666000	-5.014269000	0.456139000
H	5.531751000	-3.096088000	0.334465000
H	-1.212601000	-3.519945000	-1.524757000
H	0.319716000	-0.166877000	1.419713000
H	-5.210989000	-2.535573000	-0.473755000
H	-4.838051000	-3.793064000	0.741858000
H	-5.442017000	-2.197732000	1.268032000
H	5.191959000	-0.238130000	2.123726000
H	6.403168000	-1.312630000	1.347362000
H	6.556650000	0.463679000	1.190370000
H	-3.466754000	-0.261007000	3.378610000
H	-3.591548000	0.832000000	1.964813000
H	-4.799603000	-0.465152000	2.204570000
C	-0.749696000	1.596827000	-3.381968000
H	-0.163400000	2.454658000	-2.993546000
H	-0.156245000	1.108135000	-4.181323000
H	-1.690575000	1.980366000	-3.831307000
C	-1.827253000	-0.618627000	-2.766930000
H	-2.211396000	-1.223063000	-1.927367000
H	-2.667200000	-0.337183000	-3.433596000
H	-1.131227000	-1.257641000	-3.344703000
C	-0.168875000	4.307377000	1.671305000
H	-0.371149000	3.723798000	2.589691000
H	0.699670000	3.834021000	1.160133000
H	0.147048000	5.330311000	1.957563000
C	-1.188754000	5.259147000	-0.468530000
H	-2.107403000	5.292259000	-1.089554000
H	-0.929124000	6.294507000	-0.167744000

H	-0.367646000	4.857562000	-1.099243000
C	-3.286591000	-4.002664000	-1.942545000
H	-2.963122000	-4.686733000	-2.752615000
H	-4.187254000	-4.449767000	-1.477051000
H	-3.567195000	-3.037451000	-2.407810000
C	-1.801774000	-5.129589000	-0.197571000
H	-2.678055000	-5.493349000	0.379971000
H	-1.503135000	-5.927369000	-0.908708000
H	-0.965474000	-4.967646000	0.511691000
C	0.079520000	-1.496003000	3.121512000
H	-0.699571000	-1.802476000	3.851741000
H	0.440524000	-2.393135000	2.581016000
H	0.935202000	-1.069264000	3.683581000
C	-0.899138000	0.832573000	2.841569000
H	-1.550413000	0.642830000	3.718073000
H	0.018280000	1.330602000	3.213656000
H	-1.402837000	1.534350000	2.150826000

4''

E = -1784.0907428 au

0 1

O	-2.643946000	-0.584990000	-1.241018000
N	1.400405000	2.750787000	0.189524000
N	2.146486000	-1.425264000	1.083261000
N	1.082046000	-2.835161000	-0.209859000
N	2.115669000	1.308256000	-1.293141000
C	0.962611000	-1.597827000	0.396915000
C	-2.829115000	-1.099264000	0.039159000
C	-1.660324000	-1.362517000	0.778948000
C	-1.676590000	1.579164000	-0.815023000
C	1.024086000	1.622036000	-0.513097000
C	-4.132449000	-0.972078000	0.559835000
C	2.295096000	-0.322810000	2.048790000
H	1.450190000	0.340818000	1.764298000

C	-2.825710000	0.778788000	-1.013332000
C	2.304946000	-3.432972000	0.114430000
C	-3.230846000	-2.032478000	2.559128000
H	-3.397833000	-2.456186000	3.563377000
C	2.979054000	-2.545611000	0.933115000
C	2.703320000	3.138433000	-0.155856000
C	-1.918842000	-1.930309000	2.060692000
H	-1.073211000	-2.215830000	2.712158000
C	3.151092000	2.233834000	-1.100193000
C	0.019067000	-3.349253000	-1.103051000
H	-0.644436000	-2.464135000	-1.217533000
C	0.532053000	3.349788000	1.230915000
H	-0.435608000	2.832308000	1.076278000
C	-4.335279000	-1.495211000	1.848676000
H	-5.323405000	-1.449485000	2.332440000
C	-4.147616000	1.148537000	-0.684534000
C	-1.996075000	2.975399000	-0.799941000
H	-1.187539000	3.722310000	-0.814305000
C	2.022315000	0.259637000	-2.332281000
H	1.079813000	-0.260712000	-2.041586000
C	3.606133000	0.450760000	1.890406000
H	4.480270000	-0.097534000	2.293841000
H	3.534324000	1.407432000	2.444707000
H	3.794664000	0.696212000	0.828241000
C	-5.069426000	-0.041035000	-0.268372000
C	2.764965000	-4.764417000	-0.385516000
H	1.972379000	-5.534028000	-0.298456000
H	3.633268000	-5.111704000	0.207038000
H	3.081349000	-4.733646000	-1.449991000
C	0.539373000	-3.715309000	-2.497549000
H	1.157997000	-2.897082000	-2.917121000
H	-0.326593000	-3.860481000	-3.174092000
H	1.132269000	-4.651345000	-2.514812000
C	1.002750000	3.015856000	2.651742000

H	0.986995000	1.923847000	2.824602000
H	0.308172000	3.476691000	3.382618000
H	2.020096000	3.402837000	2.866684000
C	-3.321411000	3.431081000	-0.703944000
H	-3.519275000	4.515396000	-0.668546000
C	2.040869000	-0.811489000	3.480535000
H	1.044858000	-1.293201000	3.548239000
H	2.056348000	0.047108000	4.182860000
H	2.808756000	-1.537063000	3.819937000
C	-4.399814000	2.523968000	-0.548262000
H	-5.404176000	2.899766000	-0.299342000
C	0.318282000	4.856257000	1.029116000
H	1.165883000	5.461002000	1.408834000
H	-0.589112000	5.170373000	1.582530000
H	0.162199000	5.104829000	-0.039092000
C	4.344766000	-2.680157000	1.524648000
H	5.076080000	-1.983594000	1.061491000
H	4.722534000	-3.708055000	1.363607000
H	4.355984000	-2.490824000	2.617883000
C	3.432076000	4.307391000	0.424159000
H	3.001085000	5.277946000	0.101291000
H	4.489593000	4.288709000	0.098653000
H	3.428864000	4.298122000	1.533389000
C	-0.791651000	-4.463447000	-0.434330000
H	-0.190553000	-5.382957000	-0.271620000
H	-1.654325000	-4.732687000	-1.076901000
H	-1.191811000	-4.114769000	0.537595000
C	4.483147000	2.178049000	-1.775867000
H	5.089133000	1.308632000	-1.442403000
H	5.060490000	3.093668000	-1.545900000
H	4.394357000	2.113051000	-2.879662000
B	-0.307802000	-0.693814000	0.248977000
C	-5.608486000	-0.782116000	-1.520381000
H	-4.773525000	-1.143862000	-2.148811000

H	-6.238122000	-0.099337000	-2.128586000
H	-6.227690000	-1.651989000	-1.215882000
B	-0.327646000	0.821079000	-0.428769000
C	3.171860000	-0.749115000	-2.271165000
H	4.130856000	-0.340752000	-2.647703000
H	2.921326000	-1.622886000	-2.905989000
H	3.321759000	-1.121027000	-1.238894000
C	1.811272000	0.884137000	-3.716160000
H	0.923550000	1.547930000	-3.702939000
H	1.630000000	0.085846000	-4.464455000
H	2.688788000	1.472771000	-4.056971000
C	-6.252062000	0.448818000	0.579272000
H	-6.880264000	-0.406343000	0.903373000
H	-6.901279000	1.129239000	-0.009080000
H	-5.909511000	0.990832000	1.483554000

4'-**TS**-4"

E = -1783.9763255 au

0 1

O	2.273431000	-0.046274000	0.720653000
N	-1.721858000	1.018066000	-1.207854000
N	-2.004486000	2.687196000	0.309182000
C	2.820114000	1.078906000	0.139715000
C	-3.099893000	1.307750000	-1.119462000
C	-1.016569000	1.881594000	-0.331427000
C	-3.273989000	2.322706000	-0.214520000
C	4.208821000	1.118491000	-0.071186000
C	2.500681000	3.275849000	-0.806752000
C	1.903120000	2.098886000	-0.253327000
C	-1.711962000	4.092346000	0.648128000
C	4.736189000	2.300797000	-0.633778000
C	3.887303000	3.378954000	-0.965318000
C	5.034231000	-0.110213000	0.363164000
C	-4.529084000	2.918018000	0.332760000

C	-0.993264000	0.574360000	-2.415568000
C	-4.149213000	0.489367000	-1.799421000
B	0.422238000	1.842731000	-0.105233000
C	6.403222000	-0.157473000	-0.331866000
H	1.847162000	4.099277000	-1.135300000
H	4.321294000	4.294969000	-1.396582000
H	5.816795000	2.385892000	-0.820423000
H	-2.606804000	4.460992000	1.194592000
H	-4.567220000	2.849023000	1.444184000
H	-4.646056000	3.993617000	0.072858000
H	-5.415173000	2.390469000	-0.070188000
H	0.019735000	0.328624000	-2.024145000
H	-4.137397000	0.605706000	-2.903715000
H	-4.016489000	-0.590452000	-1.578915000
H	-5.154558000	0.791517000	-1.447431000
H	6.299643000	-0.198852000	-1.434906000
H	7.012097000	0.730847000	-0.068982000
H	6.975667000	-1.047609000	-0.002418000
N	-1.991330000	-2.530108000	-0.133337000
N	-1.637534000	-0.962222000	1.423573000
C	2.811937000	-1.236737000	0.242223000
C	4.184441000	-1.350291000	0.030673000
C	-1.004063000	-1.723039000	0.447613000
C	2.347590000	-3.457979000	-0.541339000
C	1.828703000	-2.210020000	-0.072126000
C	-3.215264000	-2.308474000	0.536867000
C	4.650503000	-2.589035000	-0.471424000
C	3.727886000	-3.623387000	-0.735670000
C	-1.533883000	-3.713066000	-0.882824000
C	-2.996594000	-1.325662000	1.478161000
C	-0.800699000	-0.357885000	2.483908000
C	-4.499876000	-2.969616000	0.159123000
B	0.411826000	-1.702909000	0.013801000
C	5.240360000	-0.045592000	1.903389000

C	-4.003751000	-0.627260000	2.331574000
H	1.660721000	-4.277944000	-0.802420000
H	4.100031000	-4.588080000	-1.116950000
H	5.722532000	-2.751047000	-0.653041000
H	-0.609418000	-3.343261000	-1.394064000
H	0.108818000	-0.032083000	1.933157000
H	-4.778027000	-2.786998000	-0.901633000
H	-4.470116000	-4.070613000	0.305412000
H	-5.320151000	-2.579673000	0.791393000
H	4.266133000	-0.009953000	2.429512000
H	5.791911000	-0.941795000	2.255640000
H	5.818248000	0.861180000	2.178957000
H	-3.801013000	-0.743767000	3.416436000
H	-4.029905000	0.460123000	2.111201000
H	-5.012794000	-1.038198000	2.137466000
C	-0.881870000	1.712798000	-3.439913000
H	-0.428683000	2.608200000	-2.968196000
H	-0.244583000	1.414136000	-4.297759000
H	-1.883756000	1.989878000	-3.832578000
C	-1.533768000	-0.720661000	-3.022792000
H	-1.806138000	-1.427011000	-2.218609000
H	-2.420889000	-0.553457000	-3.666126000
H	-0.750385000	-1.188568000	-3.652439000
C	-0.514356000	4.191827000	1.594904000
H	-0.692621000	3.634933000	2.533933000
H	0.406531000	3.779743000	1.126404000
H	-0.306573000	5.252015000	1.843756000
C	-1.526663000	4.939687000	-0.622342000
H	-2.413467000	4.854833000	-1.283607000
H	-1.371520000	6.009963000	-0.375304000
H	-0.644482000	4.577981000	-1.190822000
C	-2.497251000	-4.175642000	-1.980822000
H	-1.966811000	-4.876975000	-2.655776000
H	-3.372634000	-4.715082000	-1.570426000

H	-2.859277000	-3.324473000	-2.589802000
C	-1.135953000	-4.851423000	0.069618000
H	-2.028275000	-5.248142000	0.598114000
H	-0.665829000	-5.687810000	-0.487377000
H	-0.412684000	-4.488111000	0.826902000
C	-0.418370000	-1.420137000	3.525269000
H	-1.313122000	-1.776223000	4.079526000
H	0.062201000	-2.291332000	3.034863000
H	0.298715000	-1.001978000	4.261054000
C	-1.395396000	0.904673000	3.108005000
H	-2.178414000	0.683768000	3.860559000
H	-0.584783000	1.451709000	3.630585000
H	-1.809725000	1.570139000	2.325094000

6'

E = -1469.6643676 au

0 1

O	1.783088000	-0.010833000	-0.718667000
N	-1.418465000	-1.045744000	1.299626000
N	-2.498539000	-2.411024000	-0.055972000
C	2.380876000	-1.201747000	-0.312096000
C	-2.775610000	-0.926046000	1.588108000
C	-1.219323000	-1.933797000	0.241138000
C	-3.451251000	-1.817587000	0.770602000
C	3.738489000	-1.252146000	0.030788000
C	2.066735000	-3.543751000	0.193273000
C	1.487685000	-2.309844000	-0.226688000
C	-2.706196000	-3.339491000	-1.138722000
C	4.260962000	-2.501443000	0.443881000
C	3.429742000	-3.634187000	0.504918000
C	4.596112000	0.009433000	-0.139655000
C	-4.898718000	-2.173343000	0.723884000
C	-0.338419000	-0.309505000	1.929007000
C	-3.287196000	-0.024527000	2.657448000

B	0.024139000	-2.095885000	-0.581447000
C	5.809585000	0.005364000	0.807483000
H	1.427044000	-4.436657000	0.282358000
H	3.851011000	-4.600441000	0.826212000
H	5.321564000	-2.589053000	0.717971000
H	-1.897608000	-3.120250000	-1.886209000
H	-2.597612000	-4.400169000	-0.825979000
H	-3.708951000	-3.200937000	-1.589023000
H	-5.363581000	-1.933528000	-0.256662000
H	-5.061234000	-3.258210000	0.906469000
H	-5.457228000	-1.614504000	1.498573000
H	-0.607606000	-0.065055000	2.973601000
H	0.568037000	-0.945453000	1.920321000
H	-0.110983000	0.622715000	1.364320000
H	-2.900285000	-0.303218000	3.662136000
H	-2.986896000	1.026182000	2.464067000
H	-4.391841000	-0.061782000	2.701910000
H	5.494874000	-0.023594000	1.870202000
H	6.461356000	-0.868658000	0.611475000
H	6.435717000	0.906086000	0.651030000
N	-2.305953000	2.231699000	0.186807000
N	-2.066632000	0.901454000	-1.579824000
C	2.358007000	1.171476000	-0.298950000
C	3.704234000	1.238986000	0.085590000
C	-1.344754000	1.705871000	-0.696736000
C	2.034747000	3.545892000	0.096129000
C	1.477196000	2.297690000	-0.323028000
C	-3.596224000	1.864666000	-0.249021000
C	4.186894000	2.485550000	0.550343000
C	3.350245000	3.618134000	0.568925000
C	-1.960808000	3.300301000	1.093122000
C	-3.433676000	0.994356000	-1.306551000
C	-1.404935000	0.220194000	-2.666657000
C	-4.836958000	2.367492000	0.405849000

B	0.066003000	2.112234000	-0.786275000
C	5.099316000	0.054611000	-1.612039000
C	-4.445206000	0.227840000	-2.087017000
H	1.408958000	4.451760000	0.061710000
H	3.749825000	4.582823000	0.920320000
H	5.235667000	2.582575000	0.864706000
H	-1.057438000	3.010046000	1.672346000
H	-1.720822000	4.248837000	0.552328000
H	-2.789853000	3.491645000	1.800025000
H	-0.639496000	0.918730000	-3.089226000
H	-0.868120000	-0.693955000	-2.295954000
H	-2.133539000	-0.054224000	-3.452862000
H	-4.858144000	2.162387000	1.499041000
H	-4.965433000	3.465853000	0.279420000
H	-5.725859000	1.880260000	-0.038830000
H	4.244398000	0.059930000	-2.317315000
H	5.700388000	0.970947000	-1.789677000
H	5.724013000	-0.835725000	-1.834027000
H	-4.477324000	0.535519000	-3.155224000
H	-4.223049000	-0.861287000	-2.069807000
H	-5.457562000	0.377975000	-1.666213000

6''

E = -1469.7669032 au

0 1

O	-2.310881000	-0.412088000	-1.353752000
N	1.772237000	2.598820000	0.602623000
N	2.497099000	-1.318326000	0.938602000
N	1.644518000	-2.637397000	-0.567830000
N	2.373745000	1.414400000	-1.116519000
C	1.346469000	-1.530253000	0.203593000
C	-2.489676000	-1.187565000	-0.210983000
C	-1.321125000	-1.564442000	0.480768000
C	-1.427299000	1.659044000	-0.506896000

C	1.305482000	1.639096000	-0.273758000
C	-3.801986000	-1.211023000	0.302621000
C	2.560885000	-0.412024000	2.073631000
H	1.613896000	0.158827000	2.070775000
C	-2.546595000	0.870962000	-0.861954000
C	2.944538000	-3.091559000	-0.333225000
C	-2.887523000	-2.635134000	2.055001000
H	-3.052020000	-3.261288000	2.947819000
C	3.490414000	-2.249953000	0.615927000
C	3.088347000	2.967769000	0.306986000
C	-1.574204000	-2.391819000	1.613127000
H	-0.727207000	-2.773333000	2.210987000
C	3.470890000	2.213889000	-0.784471000
C	0.728006000	-3.189034000	-1.554895000
H	-0.101239000	-2.463135000	-1.677937000
C	1.062299000	3.023894000	1.799941000
H	0.042221000	2.601578000	1.767931000
C	-4.000863000	-1.996272000	1.451565000
H	-4.995296000	-2.082062000	1.916831000
C	-3.881505000	1.121181000	-0.480670000
C	-1.794275000	3.013222000	-0.216389000
H	-1.014640000	3.779404000	-0.074953000
C	2.288477000	0.574714000	-2.303926000
H	1.318781000	0.037345000	-2.233807000
C	-4.763562000	-0.157989000	-0.328187000
C	3.538597000	-4.250476000	-1.063763000
H	2.845914000	-5.117888000	-1.082283000
H	4.474816000	-4.579831000	-0.573402000
H	3.784013000	-4.005564000	-2.121290000
C	-3.136649000	3.390321000	-0.039306000
H	-3.375796000	4.437678000	0.209505000
C	-4.181457000	2.432904000	-0.076318000
H	-5.199537000	2.717988000	0.230764000
C	4.849530000	-2.225904000	1.232420000

H	5.438097000	-1.336487000	0.914357000
H	5.422471000	-3.125910000	0.937582000
H	4.805364000	-2.207509000	2.342457000
C	3.840776000	3.976590000	1.109739000
H	3.426102000	5.001579000	0.988283000
H	4.901890000	4.005791000	0.797116000
H	3.819339000	3.742819000	2.195764000
C	4.766526000	2.150044000	-1.522887000
H	5.279951000	1.173548000	-1.379417000
H	5.451586000	2.945540000	-1.172606000
H	4.628773000	2.287995000	-2.616387000
B	0.002754000	-0.739293000	0.139703000
C	-5.269339000	-0.641703000	-1.712846000
H	-4.418688000	-0.839997000	-2.391099000
H	-5.917789000	0.131330000	-2.176119000
H	-5.860735000	-1.574794000	-1.601718000
B	-0.062748000	0.867567000	-0.278098000
C	-5.967600000	0.109594000	0.586085000
H	-6.568902000	-0.813265000	0.719194000
H	-6.635377000	0.875723000	0.141304000
H	-5.649567000	0.464456000	1.587004000
H	1.007764000	4.131313000	1.848462000
H	1.577538000	2.631919000	2.706198000
H	2.298473000	1.201219000	-3.223924000
H	3.123809000	-0.156112000	-2.320932000
H	2.623363000	-0.986658000	3.025937000
H	3.426648000	0.277542000	1.980757000
H	0.300824000	-4.154707000	-1.209527000
H	1.253514000	-3.320653000	-2.525362000

6'-**TS**-6''

E = -1469.6618391 au

0 1

O	2.020590000	-0.017761000	0.981383000

N	-1.589043000	1.095436000	-1.426617000
N	-2.516176000	2.184821000	0.252415000
C	2.479140000	1.156617000	0.400228000
C	-2.970015000	1.031520000	-1.605310000
C	-1.275611000	1.765189000	-0.241773000
C	-3.551898000	1.748060000	-0.573645000
C	3.828813000	1.249791000	0.035459000
C	1.965811000	3.347230000	-0.451706000
C	1.479877000	2.137768000	0.140231000
C	-2.608185000	2.961866000	1.462953000
C	4.250721000	2.464037000	-0.554003000
C	3.322807000	3.507547000	-0.761712000
C	4.722635000	0.021203000	0.304461000
C	-4.983724000	2.075153000	-0.315655000
C	-0.568548000	0.474775000	-2.243996000
C	-3.599151000	0.343059000	-2.766907000
B	0.039801000	1.783806000	0.470933000
C	6.002651000	0.045023000	-0.544366000
H	1.251874000	4.151731000	-0.692007000
H	3.666669000	4.449265000	-1.219690000
H	5.297176000	2.595188000	-0.865385000
H	-1.742342000	2.650950000	2.101133000
H	-2.514757000	4.054860000	1.280877000
H	-3.565969000	2.767799000	1.984827000
H	-5.345246000	1.657700000	0.648694000
H	-5.158082000	3.173216000	-0.282471000
H	-5.624533000	1.657931000	-1.115610000
H	-0.990317000	0.180874000	-3.222435000
H	0.264238000	1.191697000	-2.392349000
H	-0.146226000	-0.412655000	-1.712786000
H	-3.267652000	0.770980000	-3.738351000
H	-3.349724000	-0.739712000	-2.784694000
H	-4.700827000	0.435074000	-2.722009000
H	5.773167000	0.055663000	-1.629030000

H	6.616070000	0.937571000	-0.307755000
H	6.630271000	-0.843244000	-0.329390000
N	-2.317726000	-2.073197000	-0.289405000
N	-2.033372000	-1.047897000	1.658560000
C	2.521674000	-1.155069000	0.378094000
C	3.863480000	-1.222279000	0.004381000
C	-1.327212000	-1.627630000	0.602876000
C	2.032740000	-3.377591000	-0.418602000
C	1.537726000	-2.156280000	0.145044000
C	-3.592692000	-1.787637000	0.225183000
C	4.295806000	-2.426135000	-0.604472000
C	3.380618000	-3.484685000	-0.793319000
C	-1.985921000	-2.923745000	-1.406246000
C	-3.407266000	-1.109218000	1.412444000
C	-1.350713000	-0.406622000	2.758763000
C	-4.849156000	-2.217195000	-0.452375000
B	0.125311000	-1.730632000	0.397506000
C	5.104864000	0.006715000	1.811822000
C	-4.409862000	-0.527231000	2.348672000
H	1.341832000	-4.214486000	-0.607280000
H	3.734753000	-4.422486000	-1.251461000
H	5.342766000	-2.546912000	-0.918001000
H	-1.103795000	-2.497252000	-1.933642000
H	-1.716519000	-3.957924000	-1.087156000
H	-2.834040000	-2.980836000	-2.114443000
H	-0.582821000	-1.103048000	3.166960000
H	-0.808016000	0.500076000	2.377902000
H	-2.071570000	-0.141241000	3.553887000
H	-4.932404000	-1.822128000	-1.487771000
H	-4.933087000	-3.325151000	-0.518096000
H	-5.729343000	-1.852596000	0.110764000
H	4.196834000	-0.010976000	2.446167000
H	5.712088000	-0.891806000	2.048423000
H	5.691279000	0.913094000	2.069962000

H	-4.353414000	-0.982493000	3.361621000
H	-4.265345000	0.567998000	2.476883000
H	-5.435496000	-0.690071000	1.966642000

4-IPr

E = -2864.9171084 au

0 1

O	-0.000260000	0.003273000	1.735550000
N	3.575535000	-2.048065000	-1.003068000
N	4.805167000	-0.225160000	-1.142006000
N	1.760168000	2.613668000	0.402335000
N	2.391736000	1.909192000	-1.631751000
C	1.129203000	-0.386457000	2.424047000
C	4.764351000	-2.404383000	-1.656094000
C	3.583211000	-0.699712000	-0.671281000
C	5.530635000	-1.258344000	-1.751381000
C	1.115154000	-0.573186000	3.824214000
C	2.180852000	1.460500000	-0.292081000
C	3.385113000	-1.194674000	2.286813000
C	2.303452000	-0.548887000	1.624579000
C	5.357418000	1.053811000	-0.643394000
C	1.832358000	3.739089000	-0.441403000
C	2.233463000	-1.195179000	4.418917000
C	3.346281000	-1.555382000	3.640749000
C	-0.000077000	0.008881000	4.699229000
C	6.850298000	-1.077884000	-2.429611000
C	2.389975000	-2.896633000	-0.764632000
C	5.080500000	-3.772514000	-2.169060000
C	1.791497000	2.680672000	1.872009000
C	2.229495000	3.315267000	-1.685764000
C	1.974866000	1.062027000	-2.771603000
C	1.534461000	5.140651000	-0.013591000
B	2.527888000	0.061587000	0.186183000
C	-0.622666000	-1.085624000	5.607903000

C	2.601800000	4.140893000	-2.875574000
H	4.311768000	-1.373443000	1.715198000
H	4.211117000	-2.053220000	4.108382000
H	2.243668000	-1.371127000	5.506048000
H	4.471354000	1.524203000	-0.171824000
H	6.765412000	-0.444593000	-3.338778000
H	7.610343000	-0.606271000	-1.772741000
H	7.250016000	-2.059901000	-2.747321000
H	1.627645000	-2.154096000	-0.434192000
H	4.871984000	-4.554372000	-1.411371000
H	4.502707000	-4.035423000	-3.080979000
H	6.154870000	-3.839769000	-2.427667000
H	1.704605000	1.632413000	2.196408000
H	1.850416000	0.060404000	-2.310884000
H	0.461968000	5.275053000	0.242798000
H	2.122316000	5.461467000	0.871118000
H	1.765255000	5.839953000	-0.839863000
H	-1.134827000	-1.854919000	4.996871000
H	0.153759000	-1.588472000	6.220458000
H	-1.373281000	-0.648054000	6.296979000
H	2.007998000	3.915289000	-3.787808000
N	-3.575900000	2.044045000	-1.010680000
N	-4.805534000	0.220688000	-1.143029000
N	-1.760012000	-2.612100000	0.411980000
N	-2.391353000	-1.915009000	-1.624726000
C	-1.129689000	0.395550000	2.422697000
C	-4.764589000	2.397964000	-1.665227000
C	-3.583675000	0.696962000	-0.673853000
C	-5.530863000	1.251581000	-1.756418000
C	-1.115501000	0.587537000	3.822159000
C	-2.180964000	-1.461554000	-0.286564000
C	-3.385707000	1.202993000	2.282663000
C	-2.304049000	0.554821000	1.622741000
C	-5.357860000	-1.056474000	-0.639887000

C	-1.831615000	-3.740521000	-0.427807000
C	-2.233794000	1.211692000	4.414624000
C	-3.346738000	1.568828000	3.635224000
C	-6.850524000	1.068660000	-2.433986000
C	-2.390308000	2.893417000	-0.775377000
C	-5.080546000	3.764190000	-2.183351000
C	-1.791674000	-2.673922000	1.881847000
C	-2.228593000	-3.321216000	-1.673743000
C	-1.974329000	-1.071745000	-2.767384000
C	-1.533318000	-5.140451000	0.005002000
B	-2.528464000	-0.061072000	0.186647000
C	0.622720000	1.106858000	5.603538000
C	-2.600233000	-4.151069000	-2.860805000
H	-4.312434000	1.379569000	1.710482000
H	-4.211554000	2.068374000	4.101070000
H	-2.243871000	1.391830000	5.501070000
H	-4.471888000	-1.525295000	-0.166654000
H	-6.765733000	0.431737000	-3.340616000
H	-7.610676000	0.599815000	-1.775248000
H	-7.250038000	2.049480000	-2.755622000
H	-1.628062000	2.152060000	-0.442066000
H	-4.872173000	4.548827000	-1.428504000
H	-4.502508000	4.023703000	-3.096091000
H	-6.154849000	3.830555000	-2.442464000
H	-1.705285000	-1.624481000	2.202547000
H	-1.850406000	-0.068437000	-2.310200000
H	-0.460754000	-5.273674000	0.261757000
H	-2.121004000	-5.458218000	0.890908000
H	-1.764043000	-5.842777000	-0.818725000
H	1.134837000	1.873735000	4.989433000
H	-0.153551000	1.612136000	6.214284000
H	1.373406000	0.671932000	6.294207000
H	-3.668583000	-4.001611000	-3.132699000
H	-2.006064000	-3.928563000	-3.773573000

H	-2.461596000	-5.226688000	-2.637825000
C	-2.602697000	3.899166000	0.362023000
H	-2.902082000	3.386731000	1.295529000
H	-1.651906000	4.434399000	0.558881000
H	-3.368052000	4.661180000	0.104728000
C	-1.883804000	3.571781000	-2.056846000
H	-1.916248000	2.878304000	-2.919794000
H	-2.462454000	4.481546000	-2.314291000
H	-0.827444000	3.874684000	-1.907043000
C	-6.386482000	-0.803919000	0.471495000
H	-6.700743000	-1.770165000	0.916856000
H	-5.936371000	-0.188116000	1.275382000
H	-7.299391000	-0.291818000	0.103010000
C	-5.861813000	-1.994535000	-1.739177000
H	-6.813543000	-1.657504000	-2.197803000
H	-5.101205000	-2.109498000	-2.533178000
H	-6.042797000	-2.997639000	-1.301828000
C	-3.028258000	-0.946390000	-3.873945000
H	-3.976131000	-0.546249000	-3.467420000
H	-2.668103000	-0.239520000	-4.650222000
H	-3.237455000	-1.912432000	-4.376634000
C	-0.602306000	-1.522569000	-3.290122000
H	-0.296967000	-0.921316000	-4.166477000
H	0.157068000	-1.395238000	-2.494253000
H	-0.599171000	-2.582364000	-3.605616000
C	-0.591161000	-3.412632000	2.484876000
H	0.353287000	-3.031546000	2.051158000
H	-0.546035000	-3.213477000	3.573277000
H	-0.635719000	-4.511059000	2.354740000
C	-3.156498000	-3.158338000	2.394447000
H	-3.205283000	-3.061675000	3.498537000
H	-3.961526000	-2.527236000	1.965750000
H	-3.359985000	-4.218260000	2.134190000
C	2.602376000	-3.898002000	0.376617000

H	2.901589000	-3.381979000	1.308198000
H	1.651652000	-4.432634000	0.575410000
H	3.367883000	-4.660858000	0.122298000
C	1.883522000	-3.579913000	-2.043491000
H	1.916129000	-2.889801000	-2.909124000
H	2.462056000	-4.490757000	-2.297351000
H	0.827104000	-3.882091000	-1.892573000
C	6.386066000	0.805371000	0.468886000
H	5.936158000	0.192096000	1.274815000
H	7.299261000	0.292453000	0.102252000
H	6.699851000	1.773253000	0.911015000
C	5.861239000	1.988017000	-1.746007000
H	6.812643000	1.649088000	-2.203909000
H	5.100305000	2.100548000	-2.540030000
H	6.042769000	2.992527000	-1.312121000
C	0.591213000	3.422060000	2.472194000
H	-0.353342000	3.040180000	2.039429000
H	0.545581000	3.226532000	3.561228000
H	0.636548000	4.520024000	2.338456000
C	3.156387000	3.166360000	2.383231000
H	3.204836000	3.073620000	3.487671000
H	3.961320000	2.533464000	1.957011000
H	3.360315000	4.225272000	2.119247000
C	3.029136000	0.932339000	-3.877327000
H	2.668837000	0.223025000	-4.651300000
H	3.238983000	1.896559000	-4.383254000
H	3.976671000	0.533083000	-3.469159000
C	0.603220000	1.511465000	-3.296554000
H	0.600560000	2.570143000	-3.615807000
H	0.298096000	0.907225000	-4.170921000
H	-0.156572000	1.387167000	-2.500625000
H	2.463246000	5.217303000	-2.656396000
H	3.670251000	3.990342000	-3.146491000
**6(B-O-B)**			

E = -1469.9135029 au

0 1

N	-1.032365000	-2.450223000	1.571967000
N	0.936720000	-1.533225000	1.819311000
N	0.936720000	-1.533225000	-1.819311000
N	-1.032365000	-2.450223000	-1.571967000
C	-0.373242000	-1.242125000	1.489308000
C	-2.482358000	-2.545379000	1.637606000
H	-2.902184000	-1.676490000	1.096079000
C	-0.145940000	-3.483053000	1.909150000
C	-0.584044000	-4.900833000	2.055052000
H	-1.359926000	-5.023366000	2.841732000
H	0.273013000	-5.545122000	2.328893000
H	-1.013283000	-5.301441000	1.109800000
C	1.096911000	-2.903046000	2.062025000
C	2.417509000	-3.517458000	2.384793000
H	3.146688000	-3.407959000	1.551612000
H	2.299821000	-4.600527000	2.579189000
H	2.879238000	-3.061703000	3.286896000
C	1.991897000	-0.538585000	1.822698000
H	1.749185000	0.221299000	1.052544000
C	-0.521808000	1.488454000	1.575474000
C	-0.282545000	1.736020000	2.954320000
H	-0.385346000	0.905656000	3.675880000
C	0.047426000	3.017597000	3.436662000
H	0.205324000	3.166907000	4.517806000
C	0.173168000	4.122307000	2.559754000
H	0.441737000	5.116108000	2.954627000
C	-0.023459000	3.908544000	1.190888000
C	-0.366588000	2.615149000	0.728243000
C	0.145459000	4.867903000	0.000000000
C	1.540138000	5.528104000	0.000000000
H	1.675457000	6.168182000	0.897274000

H	1.675457000	6.168182000	-0.897274000
H	2.339444000	4.759438000	0.000000000
C	-0.963883000	5.945842000	0.000000000
H	-1.966564000	5.473344000	0.000000000
H	-0.884488000	6.592277000	-0.899563000
H	-0.884488000	6.592277000	0.899563000
C	-0.366588000	2.615149000	-0.728243000
C	-0.023459000	3.908544000	-1.190888000
C	0.173168000	4.122307000	-2.559754000
H	0.441737000	5.116108000	-2.954627000
C	0.047426000	3.017597000	-3.436662000
H	0.205324000	3.166907000	-4.517806000
C	-0.282545000	1.736020000	-2.954320000
H	-0.385346000	0.905656000	-3.675880000
C	-0.521808000	1.488454000	-1.575474000
C	-0.373242000	-1.242125000	-1.489308000
C	-2.482358000	-2.545379000	-1.637606000
H	-2.902184000	-1.676490000	-1.096079000
C	-0.145940000	-3.483053000	-1.909150000
C	-0.584044000	-4.900833000	-2.055052000
H	-1.013283000	-5.301441000	-1.109800000
H	0.273013000	-5.545122000	-2.328893000
H	-1.359926000	-5.023366000	-2.841732000
C	1.096911000	-2.903046000	-2.062025000
C	2.417509000	-3.517458000	-2.384793000
H	2.879238000	-3.061703000	-3.286896000
H	2.299821000	-4.600527000	-2.579189000
H	3.146688000	-3.407959000	-1.551612000
C	1.991897000	-0.538585000	-1.822698000
H	1.749185000	0.221299000	-1.052544000
B	-0.928129000	0.108351000	0.928929000
B	-0.928129000	0.108351000	-0.928929000
O	-2.051447000	0.033592000	0.000000000
H	-2.830566000	-2.527632000	-2.693312000

H	-2.829977000	-3.483664000	-1.165149000
H	2.964434000	-1.014634000	-1.596016000
H	2.053677000	-0.007350000	-2.795263000
H	2.053677000	-0.007350000	2.795263000
H	2.964434000	-1.014634000	1.596016000
H	-2.829977000	-3.483664000	1.165149000
H	-2.830566000	-2.527632000	2.693312000

References

1 Sheldrick, G. Crystal Structure Refinement with SHELXL. Acta Crystallogr. Sect. C 2015, 71, 3–8.

2 Dolomanov, O. V; Bourhis, L. J.; Gildea, R. J.; Howard, J. A. K.; Puschmann, H. OLEX2: A Complete Structure Solution, Refinement and Analysis Program. J. Appl. Crystallogr. 2009, 42, 339–341.

3 1. a) Becke, A. D. Phys Rev A, 1988, 38, 3098-3101; b) Perdew, J. P. Phys. Rev. B., 1986, 33, 8822-8824; c) Grimme, S.; Antony, J.; Ehrlich, S.; Krieg, H. J. Chem. Phys., 2010, 132, 154104-154123; d) Weigend, F.; Ahlrichs, R. Phys. Chem. Chem. Phys., 2005, 7, 3297-3305; e) Weigend, F. Phys. Chem. Chem. Phys., 2006, 8, 1057-1065.

4 Gaussian 16, Revision A.03, Frisch, M. J.; Trucks, G. W.; Schlegel, H. B.; Scuseria, G. E.; Robb, M. A.; Cheeseman, J. R.; Scalmani, G.; Barone, V.; Petersson, G. A.; Nakatsuji, H.; Li, X.; Caricato, M.; Marenich, A. V.; Bloino, J.; Janesko, B. G.; Gomperts, R.; Mennucci, B.; Hratchian, H. P.; Ortiz, J. V.; Izmaylov, A. F.; Sonnenberg, J. L.; Williams-Young, D.; Ding, F.; Lipparini, F.; Egidi, F.; Goings, J.; Peng, B.; Petrone, A.; Henderson, T.; Ranasinghe, D.; Zakrzewski, V. G.; Gao, J.; Rega, N.; Zheng, G.; Liang, W.; Hada, M.; Ehara, M.; Toyota, K.; Fukuda, R.; Hasegawa, J.; Ishida, M.; Nakajima, T.; Honda, Y.; Kitao, O.; Nakai, H.; Vreven, T.; Throssell, K.; Montgomery, J. A.; Peralta, Jr. J.; Ogliaro, E. F.; Bearpark, M. J.; Heyd, J. J.; Brothers, E. N.; Kudin, K. N.; Staroverov, V. N.; Keith, T. A.; Kobayashi, R.; Normand, J.; Raghavachari, K.; Rendell, A. P.; Burant, J. C.; Iyengar, S. S.; Tomasi, J.; Cossi, M.; Millam, J. M.; Klene, M.; Adamo, C.; Cammi, R.; Ochterski, J. W.; Martin, R. L.; Morokuma, K.; Farkas, O.; Foresman, J. B.; and Fox, D. J. Gaussian, Inc., Wallingford CT, 2016.

5 a) Glendening, E. D.; Landis, C. R.; Weinhold, F. J. Comput. Chem., 2019, 40, 2234-2241; b) Reed, A. E.; Weinstock, R. B.; Weinhold, F. J. Chem. Phys., 1985, 83, 735–746.

6 Bader, R. F. W. Atoms in Molecules, Oxford University PressOxford, 1990.

7 Lu, T.; Chen, F. W. Multiwfn: A multifunctional wavefunction analyzer, J. Comput. Chem., 2012, 33, 580-592.

8 Ziegler, T.; Rauk, A. Theoret. Chim. Acta., 1977, 46, 1-10.

9 Mitoraj, M.; Michalak, A. Organometallics, 2007, 26, 6576-6580.

10 a) Velde, G. te.; Bickelhaupt, F. M.; Baerends, E. J.; Guerra, C. F.; Van Gisbergen, S. J. A.; Snijders. J. G.; Ziegler, T. J. Comput. Chem., 2001, 22, 931-967; b) ADF2019, SCM, Theoretical Chemistry, Vrije Universiteit, Amsterdam, The Netherlands, Http://www.scm.com.
